# Diterpenoid Alkaloids from *Delphinium anthriscifolium* var. majus

**DOI:** 10.1038/s41598-017-05372-3

**Published:** 2017-07-20

**Authors:** Lian-Hai Shan, Ji-Fa Zhang, Feng Gao, Shuai Huang, Xian-Li Zhou

**Affiliations:** 1 School of Life Science and Engineering, Southwest Jiaotong University, Chengdu, 610031 Sichuan P.R. China; 2 Key Laboratory of Advanced Technology of Materials, Ministry of Education, School of Material Science and Engineering, Southwest Jiaotong University, Chengdu, 610031 Sichuan P.R. China

## Abstract

Extensive phytochemical investigation on the whole herbs of *Delphinium anthriscifolium* var. *majus* led to the identification of fourteen diterpenoid alkaloids, including three new C_20_–diterpenoid alkaloids (anthriscifolsines A–C, **1**–**3**), six new C_19_–diterpenoid alkaloids (anthriscifolrines A–F, **4**–**9**), and five know compounds (**10**–**14**). Among them, anthriscifolsine A represents a novel C_20_–diterpenoid alkaloid characterized by a seco C–ring. The structures of the isolated compounds were elucidated by extensive spectroscopic methods, including HR-ESI–MS, X–ray, and 1D and 2D NMR experiments. Bioactivity of compounds **3**–**6** was evaluated for their cytotoxicity against the MCF–7, HepG2 and H460 cancer cell lines.

## Introduction

**Figure 1:**
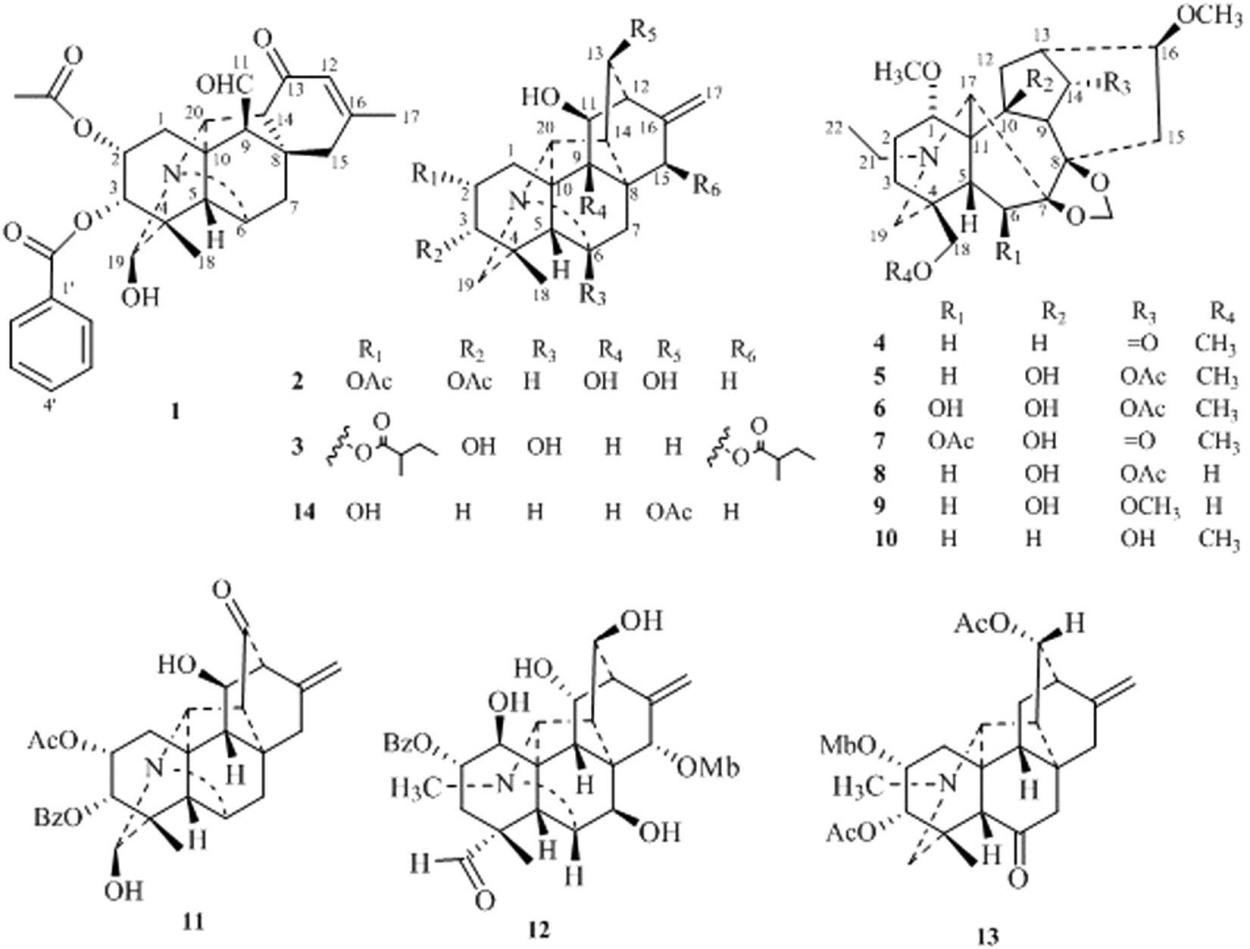
Structures of compounds **1**–**14**.

*Delphinium* is a large genus comprising 350 species and distributed in the temperate regions of the Northern Hemisphere, of which 173 are found in mainland China^1^. In our continuous phytochemical studies on the pharmacologically interesting plants of the genera *Aconitum* and *Delphinium*, we obtained a series of structurally and chemotaxonomically diverse diterpenoid alkaloids^2–5^. *Delphinium anthriscifolium* var. *majus* is an herbaceous plant, belonging to the Sect. *Anthriscifolium* of the genus *Delphinium*, and widely distributed in Guizhou, Sichuan, Hubei and Shanxi provinces in China^6^. Our earlier chemical investigation of this plant led to the discovery of two new C_18_–diterpenoid alkaloids^2^. Further studies on the whole extract of this plant resulted in the isolation and structural determination of three new C_20_–diterpenoid alkaloids, anthriscifolsines A–C (**1**–**3**), six new C_19_–diterpenoid alkaloids, anthriscifolrines A–F (**4**–**9**) (Fig. 1), and five know alkaloids nudicaulamine (**10**)^7^, anthriscifolmine C (**11**)^8^, anthriscifolmine D (**12**)^9^, anthriscifolmine I (**13**)^10^, and hetisine 13–O–acetate (**14**)^11^. Anthriscifolsine A represents a new type of C_20_–diterpenoid alkaloid, featuring a seco C–ring through an unprecedented C11–C12 bond cleavage of hetisine-type skeleton, whose stereostructure has been unambiguous established by an X–ray crystallographic analysis. Cytotoxicity of diterpenoid alkaloids against MCF–7, HepG2 and H460 cancer cell lines was also evaluated by the MTT method. Herein, we report the isolation, structural elucidation and bioactivity of these diterpenoid alkaloids.

## Results

**Figure 2:**
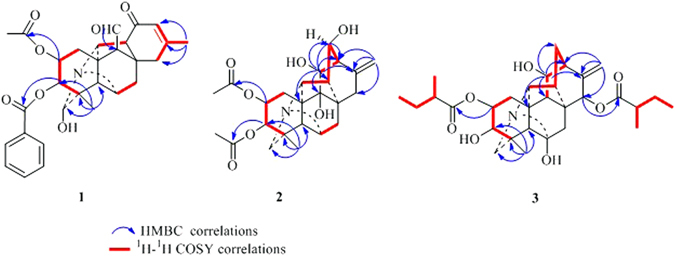
Key HMBC and ^1^H–^1^H COSY interactions of compounds **1**–**3**.

Anthriscifolsine A (**1**) was obtained as needles via crystallization from MeOH. Its molecular formula, C_29_H_31_NO_7_, was deduced from the HR–ESI–MS (*m/z* 506.2182 [M + H]^+^, calcd for C_29_H_32_NO_7_, 506.2179) and ^13^C NMR spectroscopic data. The ^1^H NMR data (Table 1) displayed characteristic resonances of two methyl [*δ*_*H*_ 1.10, 1.97 (each 3 H, s)], an acetyl [*δ*_*H*_ 2.29 (3 H, s)], a benzoyl [*δ*_*H*_ 7.32 (2 H, t, *J* = 7.8 Hz), 7.51 (1 H, t, *J* = 7.8 Hz), 7.89 (2 H, d, *J* = 7.8 Hz)], and an aldehyde [*δ*_*H*_ 9.84 (1 H, br.s)] groups. The ^13^C NMR and DEPT spectra of **1** exhibited the presence of two methyls (*δ*_*C*_ 18.8, 24.6), three methylenes (*δ*_*C*_ 33.2, 33.5, 36.3), eight sp^3^ methines (*δ*_*C*_ 54.9, 58.5, 59.1, 61.1, 63.9, 67.3, 74.3, 88.4), and three sp^3^quaternary carbons (*δ*_*C*_ 44.4, 47.1, 47.6), one trisubstituted double bond (*δ*_*C*_ 124.6, 158.5), one aldehyde (*δ*_*C*_ 201.0), one keto carbonyl (*δ*_*C*_ 196.1). In addition, an acetyl group [*δ*_*C*_ 21.6 (q), 169.8 (s)], a benzoyl group [*δ*_*C*_ 129.8 (s), 129.7 × 2 (d), 128.4 × 2 (d), 133.2 (d), 165.5 (s)] were presented in the structure according to the NMR spectra. These characteristic spectroscopic data suggested that **1** was a typical skeleton of C_20_–diterpenoid alkaloid diester^9^. The proton and corresponding carbon resonances in the 2D NMR spectra of **1** were assigned by the HMQC experiment. The existence of three oxygenated carbons deduced from its ^13^C NMR spectrum suggests that **1** has a hydroxyl group, in addition to two ester groups. The absence of a typical C–19 methylene signals in its NMR spectra suggested that a hydroxyl group might be located at C–19, which was confirmed by the HMBC correlations (Fig. 2) from H–3, H_3_–18 and H–5 to C–19^9^. The acetoxy group could be assigned to C–2 and the benzoyl group at C–3 respectively, on the basis of the HMBC correlations from H–2 (*δ*_*H*_ 5.59, m) to the carbonyl carbon of the acetyl group at *δ*_*C*_ 169.8 and H–3 (*δ*_*H*_ 5.18, d, *J* = 4.8 Hz) to the carbonyl carbon at *δ*_*C*_ 165.5 of the benzoyl group. Compound **1** has the same macular formula and similar NMR spectraoscopic data with those of anthriscifolmine C (**11**)^9^, which also possesses an acetyl group at C–2 and a benzoyl group at C–3. However, compound **1** differs from anthriscifolmine C (**11**) mainly at C–11 where an aldehyde group and a trisubstituted double bond between C–12 and C–16 were deduced. Two methys group were shown to be attached at C–4 and C–16 according to the HMBC correlations from H_3_–18 to C–3, C–4, C–5 and C–19, and H_3_–17 to C–12, C–15 and C–16. The substitution pattern and the assigned planar structure of **1** were confirmed by complete ^1^H−^1^H COSY and HMBC spectroscopic analysis.

**Table 1:** NMR Spectroscopic Data^a^ for Compounds **1–3** (600 MHz for ^1^H, 150 MHz for ^13^C, CDCl_3_, *δ* ppm).

*No*.	1		2		3	
	*δ*_H_	*δ*_C_	*δ*_H_	*δ*_C_	*δ*_H_	*δ*_C_
1	α 1.98 d (13.8) β 2.31 dd (4.8, 15.6)	33.2 t	β 1.99 dd (4.2, 15.0) α 2.98 dd (2.4, 15.0)	31.0 t	β 1.56 dd (4.2, 15.6) α 2.50 dd (1.8, 15.6)	28.8 t
2	5.59 m	67.3 d	5.35 m	68.7 d	5.29 m	71.2 d
3	5.18 d (4.8)	74.3 d	4.93 d (4.8)	74.0 d	3.67 d (5.4)	74.7 d
4	—	47.6 s	—	42.6 s	—	43.0 s
5	1.95 br.s	54.9 d	1.78 br.s	61.6 d	1.68 br.s	61.6 d
6	3.77 br.s	61.1 d	3.13 br.s	62.7 d	—	97.0 s
7	α 1.88 br.d (13.8) β 2.28 br.d (13.8)	33.5 t	β 1.39 dd (1.8,13.8) α 1.87dd (3.0,10.2)	31.8 t	β 1.81 d (12.6) α 1.85 d (13.2)	41.0 t
8	—	44.4 s	—	44.3 s	—	46.7 s
9	2.43 br.s	59.1 d	—	80.2 s	2.43 m	47.1 d
10	—	47.1 s	—	46.3 s	—	48.1 s
11	9.84 br.s	201.0 d	4.10 br.s	80.3 d	4.13 br.d (9.6)	70.2 d
12	5.93 s	124.6 d		52.7 d	2.17 m	42.9 d
13	—	196.1 s	4.26 d (8.4)	76.0 d	β 1.39 m α 2.06 dd (4.2, 9.6)	21.6 t
14	2.56 br.s	58.5 d	1.99 d (9.6)	53.3 d	1.72m	48.0 d
15	β2.47 d (19.8) α 2.76 d (19.8)	36.3 t	α 2.99 dd (2.4, 16.2) β 2.00 overlapped	31.2 t	5.54 br.s	70.6 d
16	—	158.5s	—	144.3s	—	148.5s
17	1.97 s	24.6 q	4.90 br.s 4.72 br.s	108.7 t	4.87 d (1.8) 4.98 d (1.8)	111.2 t
18	1.10s	18.8 q	1.04s	25.8 q	1.54s	27.1 q
19	5.20s	88.4 d	α 2.50 d (12.6) β 3.37 d (12.6)	59.9 t	α 3.00 d (12.0) β 3.16 d (12.0)	57.8 t
20	4.36 br.s	63.9 d	3.71 br.s	68.9 d	3.72 br.s	66.8 d
AcO–2				AcO–2		
	—	169.8 s	—	170.2 s	—	177.3 s
	2.29 s	21.6 q	2.02 s	20.9 q	2.44 m	41.9 d
BzO–3			AcO–3		1.73 m	26.8 t
1′	—	129.8s	—	170.6 s	0.91 t (7.2)	11.9 q
2′, 6′	7.89 d (7.8)	129.7 d	2.09 s	21.5 q	1.20 d (7.2)	16.8 q
3′, 5′	7.32 t (7.8)	128.4 d			MbO–15	
4′	7.51 t (7.8)	133.2 d			—	176.8 s
C = O	—	165.5 s			2.40 m	41.6 d
					1.50 m	26.7 t
					0.93 t (7.2)	11.8 q
					1.16 d (7.2)	17.0 q

^a^Data are based on DEPT, HMQC, and HMBC experiments.

**Figure 3:**
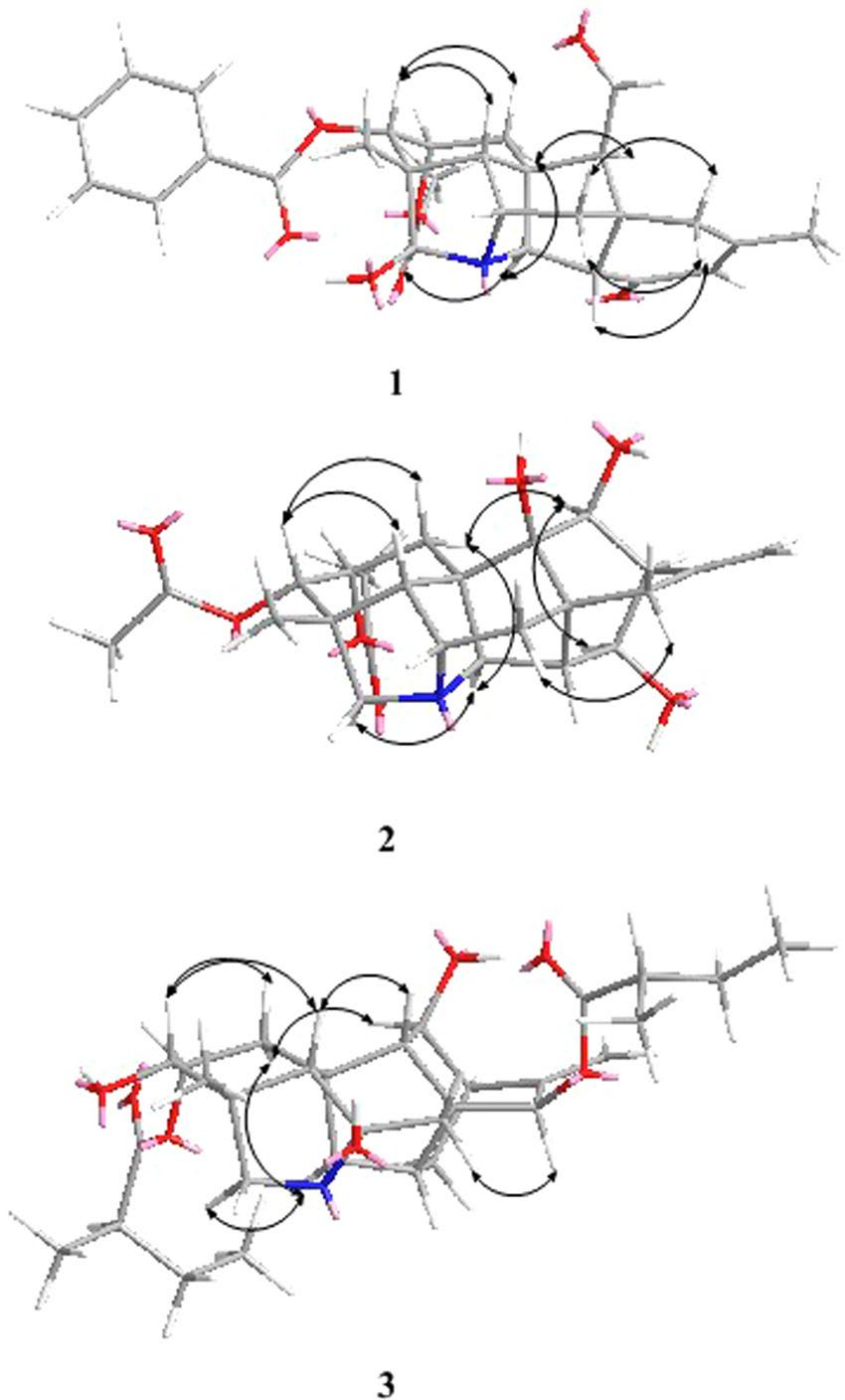
Key NOESY correlations of compounds **1–3**.

**Figure 4:**
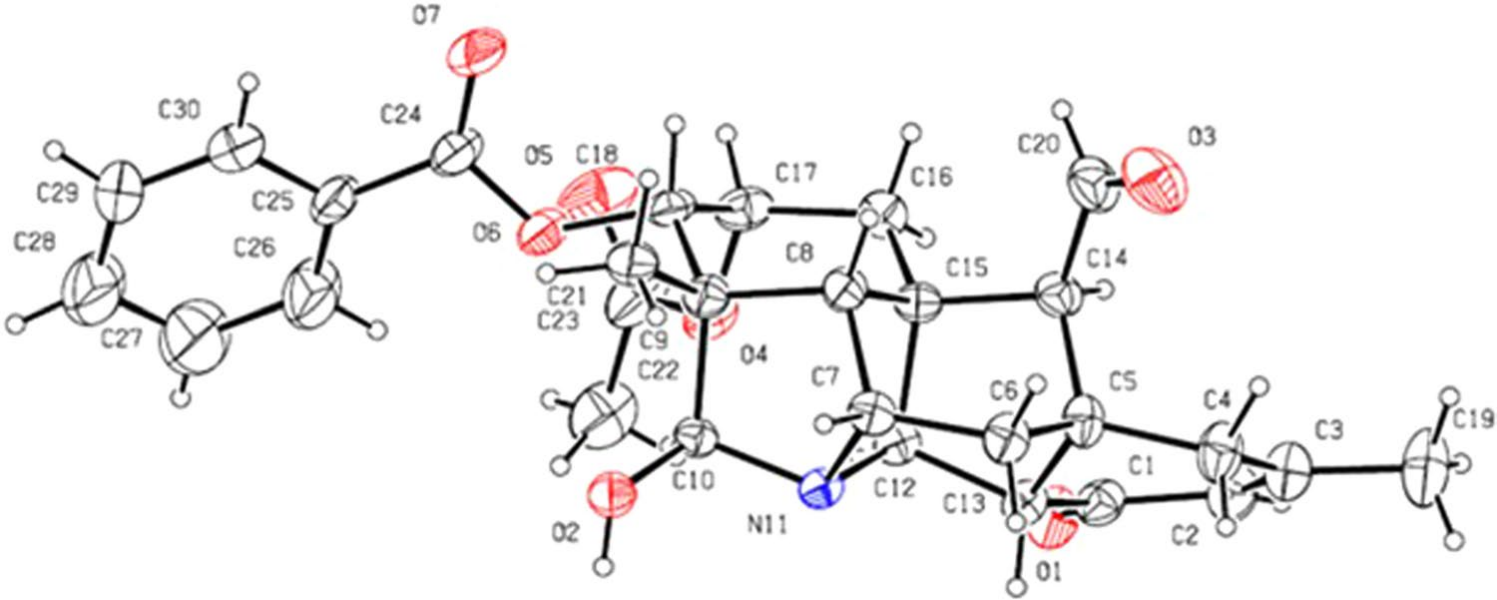
ORTEP projection of compound **1** (crystallographic numbering).

The NOESY correlations of H–1*β* and H–3, H–3 and H–5, H–1*α* and H–11, H–1*α* and H–20, H–19 and H–20, proved that H–3 was *β*–oriented, H–11 and H–19 were in α–orientation (Fig. 3). The NOESY correlations indicated that H–2 was in an equatorial position, which indicated a *β*–orientation. Moreover, an X–ray diffraction experiment with a suitable crystal was conducted and the absolute configuration of **1** was established as H–2*β*, H–3*β*, H–11*α*, H–19*α* (19–s) (Fig. 4), consistent with the absolute configuration determined by NOESY correlations. Thus, the structure of **1** was assigned as shown in Fig. 1.

**Figure 5:**
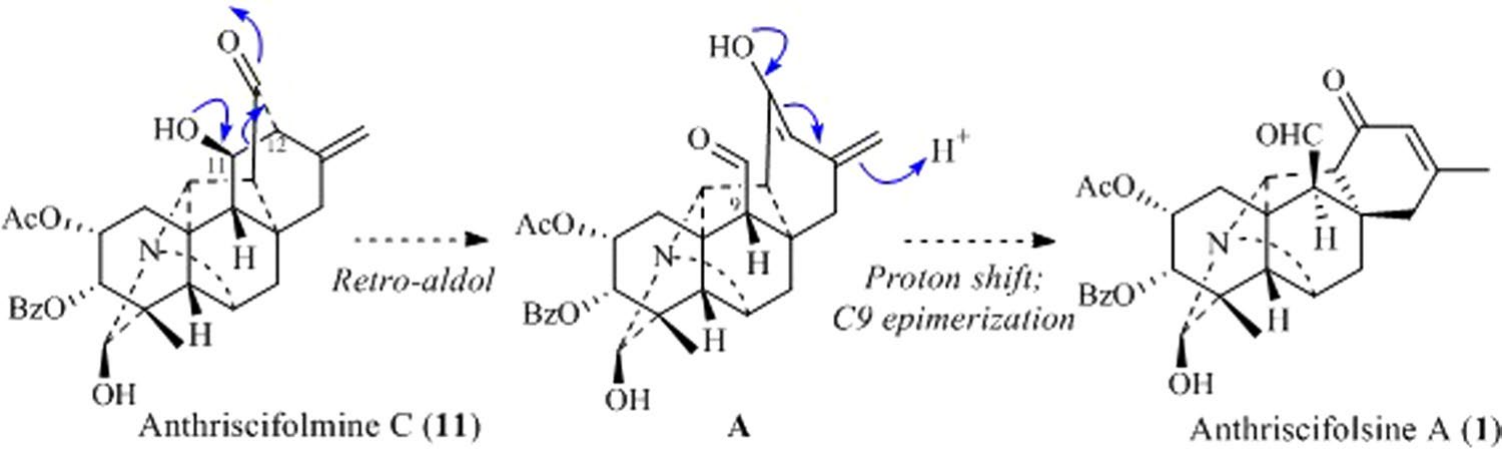
Postulated biogenetic pathway of anthriscifolsine A (**1**).

**Figure 6:**
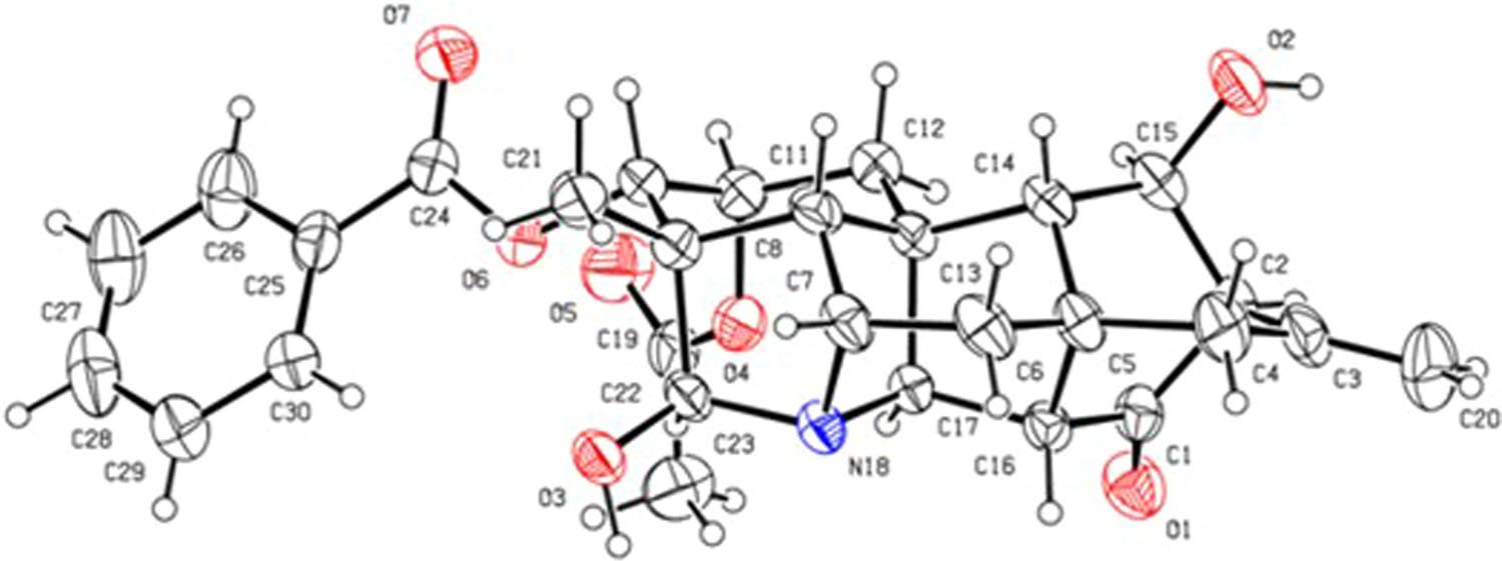
ORTEP projection of compound **11** (crystallographic numbering).

A possible biogenetic pathway of anthriscifolsine A (**1**) was proposed as shown in Fig. 5. Aldehyde **A** could be generated from the known alkaloid anthriscifolmine C (**11**) through a critical retro–aldol process involving the cleavage of C11–C12 bond. The latter has been also isolated from this plant, which was obtained as needles crystal (MeOH), and the structure of which was unambiguously confirmed by an X–ray crystallographic analysis (Fig. 6). The unstable intermediate **A** then underwent proton shift and epimerization of the C9 stereochemistry, thus leading to anthriscifolsine A (**1**). Finally, the artificial possibility of anthriscifolsine A (**1**) had been explicitly excluded used UPLC–HRESI–MS method and the detailed experiments were added the Supporting Information.

Anthriscifolsine B (**2**) was obtained as a white amorphous powder. Its molecular formula C_24_H_31_NO_7_ was derived from a pseudomolecular ion at *m/z* 446.2196 [M + H]^+^ in its HR–ESI–MS. It exhibited characteristic NMR features of a hetisine–type C_20_–diterpenoid alkaloid bearing groups including two acetyl groups, and an exocyclic double bond (Table 1)^12^. Two acetyl groups can be installed at C–2 and C–3, respectively, on the basis of the HMBC correlations from H–2 (*δ*_*H*_ 5.35, m) to the carbonyl carbon of one acetyl group at *δ*_*C*_ 170.2 and H–3 (*δ*_*H*_ 4.93, d, *J* = 4.8 Hz) to the carbonyl carbon of another acetyl group at *δ*_*C*_ 170.6. Along with the abovementioned signals, its ^13^C NMR spectrum displayed five oxygenated carbon signals, suggesting that this compound possessed three additional hydroxyl groups in addition to two ester groups. Two hydroxyl groups were assigned at C–11 and C–13 based on the HMBC correlations from H–11 to C–10, C–13 and C –16, and H–13 to C –11, and COSY correlations of H–11/H–12/H–13/H–14. The observation of HMBC crosspeak between C–9 (*δ*_*C*_ 80.2) and H–1 (*δ*_*H*_ 1.99), H–7(*δ*_*H*_ 1.39), H–12 (*δ*_*H*_ 2.51), and H–20 (*δ*_*H*_ 3.71), facilitated the location of the third hydroxyl group at C–9.

The relative configuration of **2** was deduced from the vicinal coupling constants and a NOESY experiment (Fig. 3). The coupling constant (*J* = 4.8 Hz) of H–2 with H–3, indicated that H–2 was in an equatorial position, which indicated a β–orientation^9^. The large coupling constant of H–13 (*J* = 8.4 Hz) with H–14α revealed that the dihedral angle between these two H–atoms was *ca*. 0 °C, which implied that H–13 was in an *α*–orientation^9^. In the NOESY spectrum of **2**, the cross–peak between H–1*β* and H–3, H–3 and H–5, H–1*α* and H–11, H–1*α* and H–20, proved that H–3 was *β*–oriented and H–11was in *α*–orientation. Therefore, the structure of anthriscifolsine B was determined as shown in Fig. 1, and the full assignment of its spectroscopic data was achieved based on the 1D– and 2D NMR analysis (Table 1, Fig. 2).

Anthriscifolsine C (**3**) was isolated as a white amorphous powder and its molecular formula was deduced to be C_30_H_43_NO_7_ by HR–ESI–MS at *m/z* 530.3118 [M + H]^+^. The ^1^H NMR and ^13^C NMR data (Table 1) of **3** indicated the presence of the signals of two 2–methylbutanoyloxy groups (MbO) at [(*δ*_H_ 2.44 (1 H, m), 1.73 (2 H, m), 0.91(3 H, t, *J* = 7.2 Hz), 1.20 (3 H, d, *J* = 7.2 Hz) and *δ*_C_ 177.3 (s), 41.9 (d), 26.8 (t), 11.9 (q), 16.8 (q)] and [(*δ*_H_ 2.40 (1 H, m), 1.50 (2 H, m), 0.93 (3 H, t, *J* = 7.2 Hz), 1.16 (3 H, d, *J* = 7.2 Hz) and *δ*_C_ 176.8 (s), 41.6 (d), 26.7 (t), 11.8 (q), 17.0 (q)]^2^. The remaining 20 carbons were assigned based on 1D– and 2D–NMR data and exhibited characteristic NMR features of a hetisine–type C_20_–diterpenoid alkaloid^12^ bearing five methylenes, nine methines (four oxygenated) and five quaternary carbons (one ester carbonyl), in addition to one methyl group that was attached to a quaternary carbon (Table 1). The presence of an exocyclic double bond was evidenced by singals in the ^1^H NMR spectrum (*δ*_H_ 4.87, d, *J* = 1.8 Hz, 4.98, d, *J* = 1.8 Hz) and ^13^C NMR spectrum (*δ*_C_ 111.2 and 148.5). The locations of two 2–methylbutanoyloxy groups at C–2 and C–15 were determined by the correlations in the HMBC experiment (Fig. 2). The ^13^C NMR spectrum showed a singlet at *δ*_C_ 97.0, indicative of a carbinolamine carbon (C–6). Besides the two ester groups and the carbinolamine carbon, there were two OH groups in the molecule, which were placed at C–3 and C–11, respectively, according to the HMBC displayed in Fig. 2. The coupling constant (*J* = 5.4 Hz) of H–2 with H–3 indicated that H–2 was in an equatorial position, namely, a *β*–orientation.

The key NOE correlations of H–1*β* with H–3, H–3 with H–5, H–1*α* and H–11, H–1*α* with H–20, H–15 with H–7*α*, H–15 with H–14, indicated the orientation of H–3*β*, H–11*α* and H–15*α*. On the basis of the aforementioned evidence, the structure of **3** was determined, and the trivial name anthriscifolsine C was assigned to this compound.

**Figure 7:**
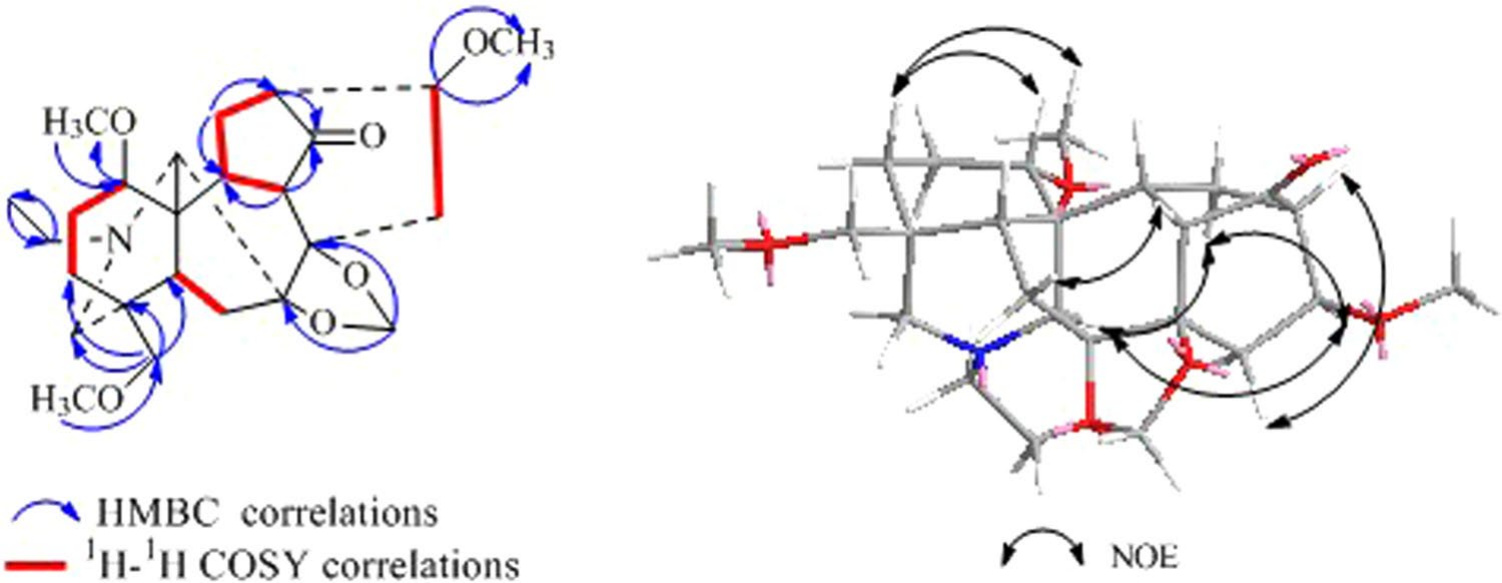
Key ^1^H–^1^HCOSY, HMBC and NOSEY correlations of **4**.

HR–ESI–MS of anthriscifolrine A (**4**) gave a molecular ion at *m/z* 448.2757 [M + H]^+^ (calcd. for C_25_H_38_NO_6_, 448.2699), corresponding to the molecular formula C_25_H_37_NO_6_. Its NMR data indicated seven methylene (one oxymethylene), seven (two oxymethines), and five quaternary carbons (a carbonyl and two oxygen–bearing), in addition to a methylenedioxy group, an *N*–ethyl, and three methoxy substituents, suggesting that **4** was a typical lycoctonine C_19_–diterpenoid alkaloid^13^. The 2D NMR and NOESY experiments confirmed the NMR data and configuration assignments of **4**. In particular, HMBC correlations of C–14 with H–9, H–12, H–13, and H–16 confirmed the 14–keto group, while HMBC correlations of the protons of the methylenedioxy with C–7 and C–8, OCH_3_–1 with C–1, OCH_3_–16 with C–16, OCH_3_–18 with C–18, confirmed the locations of the methylenedioxy and three methoxy groups. The *α*–oriented 1–OCH_3_ and *β*–oriented 16–OCH_3_ in compound **4** were deduced from the vicinal coupling constants (Table 2)and a NOESY experiment (Fig. 7). The structure of anthriscifolrine A was thus established.

**Table 2:** NMR Spectroscopic Data^a^ for Compounds **4**–**6** (600 MHz for ^1^H, 150 MHz for ^13^C, CDCl_3,_ *δ* ppm).

*No*.	4		5		6	
	*δ*_H_	*δ*_C_	*δ*_H_	*δ*_C_	*δ*_H_	*δ*_C_
1	3.85 t (5.4)	84.3 d	3.57 t (8.4)	78.1 d	3.63 m	77.2 d
2	2.27 m	26.1 t	2.40 m	26.6 t	2.18 m	26.0 t
3	1.73 m	32.4 t	*α* 1.40 m *β* 1.73 m	32.2 t	*α* 1.40 m *β* 1.77 m	31.7 t
4	—	38.6 s	—	38.1 s	—	38.3 s
5	2.02 m	46.0 d	1.80 m	39.2 d	1.83 m	45.9 d
6	1.40 m	31.7 t	*α* 2.11 m *β* 1.50 m	32.8 d	5.52 s	78.3 d
7	—	91.6 s	—	90.6 s	—	93.2 s
8	—	88.0 s	—	82.7 s	—	83.2 s
9	2.44 m	52.1 d	2.42 d (4.8)	53.4 d	3.37 m	52.3 d
10	1.55 d (7.8)	44.2 d	—	79.8 s	—	79.8 s
11	—	51.2 s	—	55.8 s	—	55.0 s
12	*α* 2.01 m *β* 2.23 m	24.9 t	*α* 1.81 m *β* 2.85 m	38.5 t	*α* 1.79 m *β* 2.50 m	33.0 t
13	2.66 m	45.6 d	2.76 m	36.2 d	2.60 m	36.6 d
14	—	213.7s	5.26 t (5.4)	74.7 d	4.64 m	72.9 d
15	*α* 2.09 m *β* 1.42 m	31.5 t	*α* 2.55 dd (9.6,16.2) *β* 1.74 m	34.4 t	*α* 2.45 m	37.4 t
16	3.15 dd (6.6, 10.8)	84.8 d	3.20 q (5.4, 9.6)	81.2 d	3.47 t (8.8)	81.3 d
17	3.59 brs	63.5 d	2.98 brs	65.1 d	3.34 m	64.9 d
18	3.01 d (9.0) 3.08 d (9.0)	78.9 t	3.00 d (9.0) 3.13 d (9.0)	79.1 t	3.05 d (9.2) 3.18 d (9.2)	78.3 t
19	2.45 m 2.63 m	52.7 t	2.45 m 2.65 d (11.4)	52.6 t	2.42 m 2.78 m	53.4 t
21	2.46 m 2.85 dd (7.2, 12.6)	51.0 t	2.70 dd(7.2, 12.6) 2.81 dd(7.2, 12.6)	50.7 t	2.83 m	50.6 t
22	1.09 t (7.2)	14.3 q	1.07 t (7.2)	14.1 q	1.07 t (7.2)	14.1 q
1–OCH_3_	3.29 s	56.1 q	3.27 s	55.8 q	3.25 s	55.7 q
16–OCH_3_	3.31 s	56.2 q	3.28 s	56.3 q	3.26 s	56.5 q
18–OCH_3_	3.34 s	59.6 q	3.29 s	59.6	3.35 s	59.5 q
O–CH_2_–O	4.95 s, 5.04 s	94.1 t	4.95 s, 5.01 s	93.8 t	4.96 s, 4.98 s	94.3 t
14– OAc			—	171.9s	—	170.2 s
			2.07 s	21.5 q	2.10 s	21.8 q

^a^Data are based on DEPT, HMQC, and HMBC experiments.

The molecular formula of anthriscifolrine B (**5**) was determined as C_27_H_41_NO_8_ by HR–ESI–MS at *m/z* 508.2952 [M + H]^+^ (calcd. for C_27_H_42_NO_8_, 508.2910). The NMR spectroscopic data of **5** were similar to those of **4**, suggesting **5** was also a lycoctonine C_19_–diterpenoid alkaloid with an acetyl group, an *N*–ethyl group, three methoxyl groups, and a methylenedioxy group^13^. In the HMBC spectrum of **5**, critical correlations for the protons of the methylenedioxy/C–7 and C–8, OCH_3_–1/C–1, OCH_3_–16/C–16, OCH_3_–18/C–18, H–14/OAc, suggested the location of three methoxyl groups at C–1, C–16 and C–18, the acetyl group at C–14, and the methylenedioxy group at C–7 and C–8. Its ^13^C NMR spectrum displayed seven oxygenated carbon signals, suggesting that it possessed an additional hydroxyl group in addition to three methoxy groups, an ester group, and a methylenedioxy group. The additional hydroxyl group in **5** was assigned to C–10 on the basis of the correlations of C–10 (*δ*c 79.8) with H–1 (*δ*_H_ 3.57), H–9 (*δ*_H_ 2.42), H–13 (*δ*_H_ 2.76) and H–17 (*δ*_H_ 2.98) in the HMBC spectrum. The configurations of 1*α*–OCH_3_, 16*β*–OCH_3_, 14*α*–OAc and constant18*β*–OCH_3_ were deduced by the vicinal coupling constants (Table 2) and a NOESY experiment. Thus, the structure of anthriscifolrine B was confirmed by NMR experiments.

The molecular formula of anthriscifolrine C (**6**) was determined as C_27_H_41_NO_9_ (HR–ESI–MS). The ^1^H and ^13^C NMR data (Table 2) of **6** showed close structural similarity to compound **5**, and the distinction between the two sets of spectra was demonstrated by the presence of an additional hydroxyl group signal in **6**, which was validated by the additional 16 mass units in mass spectrometry. The proton signal of H–6 at [*δ*_*H*_ 2.11 (1 H, m), 1.50 (1 H, m) and *δ*_*C*_ 32.8 (t)] in compound **5** was shifted downfield to [*δ*_*H*_ 5.52 (1 H, s), *δ*_*C*_78.3(d)] in compound **6**, suggesting that the hydroxyl group in **6** might be located at C–6, which was further confirmed by the HMBC correlations. The hydroxyl group at C–6 was determined to have a *β*–orientation based on the multiplicity of H–6 (singlet) in the ^1^H NMR spectrum^2^. Thus, the structure of anthriscifolrine C was determined as shown in Fig. 1.

Anthriscifolrine D (**7**), a white amorphous powder, C_27_H_39_NO_9_ (HR–ESI–MS), was also a lycoctonine–type C_19_–diterpenoid alkaloid. By comparison of the NMR data of **7** with those of **4**, the main difference was the presence of a acetyl and a hydroxyl groups. In the HMBC experiment, long–range correlations were observed from H–6 (5.54) to the carbonyl carbon of the acetyl group at *δ*_*C*_ 170.2, and H–1 (*δ*_H_ 3.91), H–9 (*δ*_H_ 3.49), H–13 (*δ*_H_ 2.82) and H–17 (*δ*_H_ 3.71) to C–10 (*δ*c 79.8) supported the hydroxyl group at C–10. The structure of anthriscifolrine D (Fig. 1) was confirmed by the analysis of its 2D NMR data.

The molecular formula of anthriscifolrine E (**8**) was deduced to be C_26_H_39_NO_8_ from its HR–ESI–MS at *m/z* 494.2752 [M + H]^+^. From its NMR data (Table 3), an *N*–ethyl group, two methoxy groups, an acetoxy group, and a methylenedioxy group could be easily recognized. Compound **8** shared highly similar ^1^H– and ^13^C–NMR spectral patterns with those of **5**. The only difference is that the absence of a methoxy group and the presence of a hydroxy group at C–18 in **8**, which was further supported by comparison of the NMR data: the C–18 signal in **8** appeared at *δ*_*C*_ 68.3 instead of at *δ*_*C*_ 79.1 in **5**. The structure of anthriscifolrine E was unquestionably confirmed by extensive analyses of its 1D and 2D NMR spectra.

**Table 3:** NMR Spectroscopic Data^a^ for Compounds **7**–**9** (600 MHz for ^1^H, 150 MHz for ^13^C, CDCl_3,_ *δ* ppm).

*No*.	7		8		9	
	*δ*_H_	*δ*_C_	*δ*_H_	*δ*_C_	*δ*_H_	*δ*_C_
1	3.91 t (5.4)	83.8 d	3.60 t (7.2)	78.0 d	3.55 t (5.4)	78.2 d
2	2.15 m	25.6 t	*α* 2.12 m *β* 2.35 m	26.6 t	2.12 m 2.35 m	26.7 t
3	1.40 m 1.70 m	31.5 t	1.70 m	31.6 t	1.49 m	32.1 t
4	—	38.5 s	—	38.4 s	—	38.4 s
5	1.86 brs	45.9 d	1.75 m	38.4 d	2.02 m	38.6 d
6	5.54 s	77.9 d	1.45 m 2.12 m	32.6 t	1.45 m 2.15 m	32.7 t
7	—	92.7 s	—	90.6 s	—	90.3 s
8	—	87.0 s	—	79.9 s	—	80.2 s
9	3.49 s	58.5 d	2.45 d (4.8)	53.2 d	2.31 m	54.0 d
10	—	79.8 s	—	82.7 s	—	83.4 s
11	—	55.0 s	—	56.3 s	—	56.5 s
12	1.69 m 2.38 dd (6.0,16.8)	31.1 t	*α* 1.90 d (7.2) *β* 2.85 m	38.6 t	*α* 1.70 m *β* 3.01 d (15.6)	39.4 t
13	2.82 m	45.3 d	2.75 m	36.4 d	2.55 m	38.0 d
14		213.2 s	5.26 t (5.4)	74.7 d	4.12 t (4.8)	81.7 s
15	1.96 dd (7.2,15.6) 2.74 m	36.2 t	*α* 2.55 dd (9.6, 16.2) *β* 1.74 m	34.4 t	*α* 2.50 m *β* 1.89m	34.1 t
16	3.77 dd (7.2, 10.2)	76.9 d	3.20 dd (4.8, 9. 0)	81.2 d	3.17 dd (4.8, 9.0)	81.7 d
17	3.71 d (2.4)	65.3 d	2.99 brs	62.2 d	2.97 brs	61.9 d
18	3.04 d (9.6) 3.14 d (9.6)	78.1 t	3.25 m 3.40 d (11.4)	68.3 t	3.31 m 3.43 m	68.5 t
19	2.49 m 2.75 m	53.3 t	2.38 d (11.4) 2.61 d (11.4)	52.4 t	2.41 d (11.4) 2.62 d (11.4)	52.4 t
21	2.73 m 2.87 m	50.7 t	2.72 m 2.82 m	50.8 t	2.70 m 2.82 m	50.7 t
22	1.09 t (7.2)	14.1 q	1.08 t (7.2)	14.1 q	1.08 t (7.2)	14.3 q
1–OCH_3_	3.31 s	55.8 s	3.27 s	55.8 q	3.28 s	55.8 q
14–OCH_3_	—	—	—	—	3.45 s	58.0 q
16–OCH_3_	3.34 s	56.3 q	3.28 s	56.3 q	3.33 s	56.5 q
18–OCH_3_	3.24 s	59.5 q	—	—	—	—
O–CH_2_–O	4.95 s, 4.96 s	94.8 t	4.94 s, 5.01 s	93.8 t	4.94 s, 5.02 s	93.9 t
6–OAc		170.2 s	—	—	—	—
	2.08 s	21.7 q	—	—	—	—
14–OAc	—	—	—	172.1 s	—	—
	—	—	2.08 s	21.5 q	—	—

^a^Data are based on DEPT, HMQC, and HMBC experiments.

Comparison of spectroscopic data of anthriscifolrine F (**9**) and E (**8**) indicated that an acetyl group in **8** was substituted by a methoxy group in **9**. According to 2D NMR analysis, especially the HMBC correlation of OCH_3_/C–14, the OCH_3_ group was attributed to C–14 in **9**. The corresponding structure of **9** was confirmed by DEPT, HMQC, ^1^H–^1^H COSY, and HMBC experiments. Thus, anthriscifolrine F was assigned as shown in Fig. 1.

To evaluate the biological activities of these compounds isolated from the whole plant of *D.anthriscifolium* var. *majus*, compounds **3**–**6** were tested for their *in vitro* cytotoxicity against the MCF–7, HepG2 and H460 cancer cell lines. Unfortunately, all of the compounds were inactive (IC_50_ > 50 *μ*M, *n* = 3).

## Discussion

Investigation on the whole plant of *Delphinium anthriscifolium* var. *majus* resulted in the isolation of nine new diterpenoid alkaloids named anthriscifolsines A–C (**1**–**3**) and anthriscifolrines A–F (**4**–**9**), together with five known alkaloids (**10**–**14**). Notably, anthriscifolsine A (**1**) is the first naturally occurring C_20_–diterpenoid alkaloid with a unique seco C–ring generated by an unprecedented C11–C12 bond cleavage, and its possible biogenetic pathway was proposed. Since the Sect. *Anthriscifolium* only comprises three species (*D. anthriscifolium*, *D. anthriscifolium* var. *majus*, and *D. anthriscifolium* var. *savatieri*), the present research would be particularly valuable in understanding their chemotaxonomical significance. The identification of various C_19_– and C_20_–diterpenoid alkaloids from *D. anthriscifolium* var. *majus* revealed its transitional position among the *Delphinium* plants.

## Materials and Methods

### General Experimental Procedures

Optical rotations were measured using a Perkin–Elmer 341 polarimeter. The IR spectra were obtained using a Thermo Fisher Nicolet 6700 spectrometer and KBr pellets in cm^−1^. The HR–ESI–MS data were measured using a Q–TOF micro mass spectrometer (Waters). The 1D and 2D NMR spectra were recorded using a Bruker AV 600 with TMS. Silica gel (Qingdao Haiyang Chemical Co., Ltd., 200–300 mesh) was used for column chromatography (CC). The TLC plates were precoated with silica gel GF_254_ (Qingdao Haiyang Chemical Co., Ltd., China), and it was visualized under a UV lamp at 254 nm or by spraying with Dragendorff’s reagent or iodine.

### Plant Material

The whole herbs of *D. anthriscifolium* var. *majus* were collected in Longshanwa, Zhuxi county, Hubei province of China, in April 2015, and were identified (voucher specimen: L H. Shan & J X. Wang 801) by Prof. Qing–Er Yang of the Institute of Botany, Chinese Academy of Sciences.

### Extraction and Isolation

Dried and powdered whole herbs of *D. anthriscifolium* (21.5 kg) were extracted with 95% EtOH four times at room temperature, with each soaking process lasting a week. After removal of the solvent by evaporation, the ethanol extract (2000 g) was recovered. The extract was suspended in H_2_O (3 L) and adjusted to pH 2 with HCl, and successively extracted with petroleum ether (4 × 1 L) and ethyl acetate (4 × 1 L). The pH of aqueous layer was adjusted to 10 with aqueous ammonia solution and extracted with CH_2_Cl_2_ (4 × 1 L). The CH_2_Cl_2_ extracts were concentrated to produce the crude alkaloid extract (28.5 g). Column chromatography of the crude alkaloid extract over silica gel, using a CH_2_Cl_2_:MeOH (80:1, v/v) mixture with increasing polarity afforded fractions A–D based on TLC analysis.

Fraction A (10.7 g) was submitted to silica gel CC eluting with petroleum ether/Me_2_CO/Et_2_N (50: 1: 0.1 to 20:1:0.1, v/v/v) to yield compounds **4** (14 mg), **10** (20 mg) respectively.

Fraction B (7.2 g) was submitted to silica gel CC eluting with petroleum ether/Me_2_CO/Et_2_N (15: 1: 0.1 to 6:1:0.1, v/v/v) to yield compounds **5** (20 mg), **6** (43 mg) and **12** (8 mg).

Fraction C (5.6 g) was subjected to silica gel CC, petroleum ether/Me_2_CO/Et_2_N (15: 1: 0.1 to 6:1:0.1, v/v/v) to yield compounds **1** (16 mg), **11** (14 mg), **7** (10 mg) and **3** (4.9 mg).

Fraction D (5.0 g) was subjected to silica gel CC, eluted with CH_2_Cl_2_:MeOH (30:1 to 10:1, v/v) to get four fractions (D_1_–D_4_), fraction D_1_ was subjected to Sephadex LH–20 column chromatography (MeOH) to yield compounds **13** (14 mg) and **14** (8 mg), fraction D_2_ was further purified using an RP–18 silica gel column with MeOH: H_2_O (10:90 to 30:70, v/v) as the mobile phase to yield compounds **2** (7.6 mg), **8** (20 mg), and **9** (11 mg).

### Spectroscopic data of 1–9

**Anthriscifolsine** A (**1)**: needle crystal (MeOH); ${[{\rm{\alpha }}]}_{{\rm{D}}}^{20}$ + 11.1 (*c* 0.38, CHCl_3_); IR (KBr) *v*_max_: 3465, 3070, 2928, 2880, 2854, 2747, 1744, 1716, 1667, 1630, 1450, 1381, 1340, 1276, 1233, 1121, 1062, 1038, 997, 965, 942, 909, 752, 715; ^1^H and ^13^C NMR data see Table 1; HR–ESI–MS at *m/z* 506.2182 [M + H]^+^ (calcd. for C_29_H_32_NO_7_, 506.2179).

**Anthriscifolsine** B (**2)**: white, amorphous powder; ${[{\rm{\alpha }}]}_{{\rm{D}}}^{20}$ + 1.1 (*c* 0.38, CHCl_3_); IR (KBr) *v*_max_: 3408, 2925, 2853, 1741, 1655, 1371, 1251, 1063, 1042, 985, 769, 719; ^1^H and ^13^C NMR data see Table 1; HR–ESI–MS at *m/z* 446.2196 [M + H]^+^ (calcd. for C_24_H_32_NO_7_, 446.2179).

**Anthriscifolsine** C (**3)**: white, amorphous powder; ${[{\rm{\alpha }}]}_{{\rm{D}}}^{20}$ −9.8 (*c* 0.25, CHCl_3_); IR (KBr) *v*_max_: 3443, 2967, 2935, 2878, 1727, 1656, 1462, 1383, 1265, 1237, 1187, 1152, 1077, 1135, 1009, 997, 906, 754, 715; ^1^H and ^13^C NMR data see Table 1; HR–ESI–MS at *m/z* 530.3118 [M + H]^+^ (calcd. for C_30_H_44_NO_7_, 530.3118).

**Anthriscifolrine** A (**4)**: white, amorphous powder; ${[{\rm{\alpha }}]}_{{\rm{D}}}^{20}$ −4.2 (*c* 0.50, CHCl_3_); IR (KBr) *v*_max_: 3423, 2953, 2925, 2854, 1752, 1648, 1463, 1377, 1094, 954, 734, 721; ^1^H and ^13^C NMR data see Table 2; HR–ESI–MS at *m/z* 448.2757 [M + H]^+^ (calcd. for C_25_H_38_NO_6_, 448.2699).

**Anthriscifolrine** B (**5)**: white, amorphous powder; ${[{\rm{\alpha }}]}_{{\rm{D}}}^{20}$ −26.7 (*c* 0.30, CHCl_3_); IR (KBr) *v*_max_: 3472, 2958, 2923, 2875, 2823, 2755, 1740, 1718, 1456, 1369, 1248, 1206, 1092, 1075, 1054, 961, 934, 755, 730; ^1^H and ^13^C NMR data see Table 2; HR–ESI–MS at *m/z* 508.2952 [M + H]^+^ (calcd. for C_27_H_42_NO_8_, 508.2910).

**Anthriscifolrine** C (**6)**: white, amorphous powder; ${[{\rm{\alpha }}]}_{{\rm{D}}}^{20}$ −2.3 (*c* 0.70, CHCl_3_); IR (KBr) *v*_max_: 3438, 2972, 2927, 2875, 2829, 2750, 1738, 1666, 1453, 1387, 1368, 1246, 1232, 1090, 1045, 961, 918, 756, 715; ^1^H and ^13^C NMR data see Table 2; HR–ESI–MS at *m/z* 524.2867 [M + H]^+^ (calcd. for C_27_H_42_NO_9_, 524.2860).

**Anthriscifolrine** D (**7)**: white, amorphous powder; ${[{\rm{\alpha }}]}_{{\rm{D}}}^{20}$ −12.8 (*c* 0.50, CHCl_3_); IR (KBr) *v*_max_: 3461, 2962, 2924, 2873, 2854, 2827, 2752, 1757, 1742, 1647, 1456, 1368, 1245, 1227, 1103, 1088, 1043, 958, 926, 761, 742; ^1^H and ^13^C NMR data see Table 3; HR–ESI–MS at *m/z* 522.2702 [M + H]^+^ (calcd. for C_27_H_40_NO_9_, 522.2703).

**Anthriscifolrine** E (**8)**: white, amorphous powder; ${[{\rm{\alpha }}]}_{{\rm{D}}}^{20}$ −10.7 (*c* 0.30, CHCl_3_); IR (KBr) *v*_max_: 3447, 2962, 2926, 2885, 2857, 2818, 2746, 1741, 1717, 1463, 1370, 1265, 1230, 1092, 1075, 1052, 953, 913, 756, 729, 710; ^1^H and ^13^C NMR data see Table 3; HR–ESI–MS at *m/z* 494.2752 [M + H]^+^ (calcd. for C_26_H_40_NO_8_, 494.2754).

**Anthriscifolrine** F (**9)**: white, amorphous powder; ${[{\rm{\alpha }}]}_{{\rm{D}}}^{20}$ −15.0 (*c* 0.30, CHCl_3_); IR (KBr) *v*_max_: 3528, 3382, 2958, 2925, 2889, 2855, 2817, 1742, 1666, 1464, 1388, 1371, 1327, 1239, 1158, 1132, 1045, 1103, 1087, 1072, 1051, 1001, 970, 955, 921, 781, 757, 731; ^1^H and ^13^C NMR data see Table 3; HR–ESI–MS at *m/z* 466.2817 [M + H]^+^ (calcd. for C_25_H_40_NO_7_, 466.2805).

Crystal Data of **1**: C_29_H_31_NO_7_, *M*r 505.21, *a* = 12.9571(5) Å, *b* = 20.9703(17) Å, c = 21.7049(12) Å, *V* = 5897.5(6) Å^3^, space group *P*2_1_2_1_2_1_, Z = 8, *D*_calc_ = 1.204 Mg/m^3^, λ = 0.71073 Å, *μ*(Moka) = 0.086 mm^−1^, *F*(000) = 2272.0, and *T* = 293.15 K; Data were collected using an orthorhombic of size 0.4 × 0.1 × 0.05 mm^3^ in the range −15 ≤ *h* ≤ 16, −24 ≤ *k* ≤ 26, −27 ≤ *l* ≤ 16. 21557 reflections measured, 11486 unique reflections *R*_int_ = 0.0297. Refinement by full–matrix least–squares on *F* ^2^ converged to give final *R* indices *R*_1_ = 0.0781, *wR*_2_ = 0.1779 [I > 2σ(*I*)] and *R*_1_ = 0.1399, *wR*_2_ = 0.2106 (all data).

Data/restraints/parameters = 11486/1/725, goodness–of–fit on *F* ^2^ = 0.988, largest difference peak and hole are 0.25 and −0.19 e Å^−3^. Crystallographic data for **1** have been deposited with the Cambridge Crystallographic Data Center as supplementary publication number CCDC 1487703. These data can be obtained free of charge via www.ccdc.cam.ac.uk/deposit (or from the CCDC, 12 Union Road, Cambridge CB2 1EZ, UK; fax: + 44 1223336033; deposit@ccdc.cam.ac.uk).

Crystal Data of **11**: C_29_H_31_NO_7_, *M*r 505.21, *a* = 13.1758(7) Å, *b* = 18.3883(9) Å, c = 21.9022(13) Å, *V* = 5306.5(5) Å^3^, space group *P*2_1_2_1_2_1_, Z = 8, *D*_calc_ = 1.263 Mg/m^3^, λ = 0.71073 Å, *μ*(Moka) = 0.090 mm^−1^, *F*(000) = 2136.0, and *T* = 293.15 K; Data were collected using an orthorhombic of size 0.4 × 0.08 × 0.08 mm^3^ in the range −16 ≤ *h* ≤ 15, −13 ≤ *k* ≤ 22, −23 ≤ *l* ≤ 27. 17628 reflections measured, 9715 unique reflections *R*_int_ = 0.0250. Refinement by full–matrix least–squares on *F* ^2^ converged to give final *R* indices *R*_1_ = 0.0551, *wR*_2_ = 0.1345 [I > 2σ(*I*)] and *R*_1_ = 0.07969, *wR*_2_ = 0.1498 (all data).

Data/restraints/parameters = 9715/0/687, goodness–of–fit on *F* ^2^ = 0.999, largest difference peak and hole are 0.36 and −0.14 e Å^−3^. Crystallographic data for **11** have been deposited with the Cambridge Crystallographic Data Center as supplementary publication number CCDC 1487702. These data can be obtained free of charge via www.ccdc.cam.ac.uk/deposit (or from the CCDC, 12 Union Road, Cambridge CB2 1EZ, UK; fax: + 44 1223336033; deposit@ccdc.cam.ac.u k).

### Cell Culture and Cytotoxicity Assay

The cytotoxicity of the compounds against cultured human tumor cell lines such as MCF–7, HepG2 and H460 cell lines was evaluated by the MTT method as described in our previous paper^14^. Cells treated with DMSO (0.1% *v*/*v*) were used as negative controls, whereas adriamycin (≥98%; Sigma Chemical Co., Ltd., Shanghai, China) was used as the positive control.

## Electronic supplementary material

Supplementary Info
