# Supplementary material for: Diterpenoid Alkaloids from Delphinium anthriscifolium var. majus
Source: Sci Rep. 2017 Jul 20;7:6063. doi: 10.1038/s41598-017-05372-3 (PMC5519776; doi:10.1038/s41598-017-05372-3)

Diterpenoid Alkaloids from *Delphinium anthriscifolium* var. *majus*

Lian–Hai Shan[1, 2], Ji–Fa Zhang [1], Feng Gao [1], Shuai Huang [1]& Xian–Li Zhou [1, 2]

[1] School of Life Science and Engineering, Southwest Jiaotong University, Chengdu 610031, Sichuan, P.R. China. [2]Key Laboratory of Advanced Technology of Materials, Ministry of Education, School of Material Science and Engineering, Southwest Jiaotong University, Chengdu 610031, Sichuan, P. R. China. Correspondence and requests for materials should be addressed to S.H. (email: shuaih@swjtu.edu.cn) or X.-L.Z. (email: zhouxl@swjtu.edu.cn).

S *Supporting Information*

Correspondence

Dr. Shuai Huang; Prof. Dr. Xian-li Zhou

Natural Products Laboratory of School of Life Science and Engineering, Southwest Jiaotong University, Chengdu 610031, Sichuan, People's Republic of China.

Phone: +86-28-66367232

Fax: +86-28-66367260

E-mail: shuaih@swjtu.edu.cn; zhouxl@swjtu.edu.cn;

1 HR-ESI-MS spectrum for compound **1**
2 IR spectrum for compound **1**
3 ^{1}H NMR spectrum for compound **1**
4 ^{13}C NMR spectrum for compound **1**
5 DEPT (θ = 135°) spectrum for compound **1**
6 ^{1}H-^{1}H COSY spectrum for compound **1**
7 HMQC spectrum for compound **1**
8 HMBC spectrum for compound **1**
9 Noesy spectrum for compound **1**
10 X-Ray spectrum for compound **1**
11 HR-ESI-MS spectrum for compound **2**
12 IR spectrum for compound **2**
13 ^{1}H NMR spectrum for compound **2**
14 ^{13}C NMR spectrum for compound **2**
15 DEPT (θ = 135°) spectrum for compound **2**
16 ^{1}H-^{1}H COSY spectrum for compound **2**
17 HMQC spectrum for compound **2**
18 HMBC spectrum for compound **2**
19 Noesy spectrum for compound **2**
20 HR-ESI-MS spectrum for compound **3**
21 IR spectrum for compound **3**
22 ^{1}H NMR spectrum for compound **3**
23 ^{13}C NMR spectrum for compound **3**
24 DEPT (θ = 135°) spectrum for compound **3**
25 ^{1}H-^{1}H COSY spectrum for compound **3**
26 HMQC spectrum for compound **3**
27 HMBC spectrum for compound **3**
28 Noesy spectrum for compound **3**
29 HR-ESI-MS spectrum for compound **4**
30 IR spectrum for compound **4**
31 ^{1}H NMR spectrum for compound **4**
32 ^{13}C NMR spectrum for compound **4**

33 DEPT (θ = 135°) spectrum for compound **4**

34 ^{1}H-^{1}H COSY spectrum for compound **4**

35 HMQC spectrum for compound **4**

36 HMBC spectrum for compound **4**

37 Noesy spectrum for compound **4**

38 HR-ESI-MS spectrum for compound **5**

39 IR spectrum for compound **5**

40 ^{1}H NMR spectrum for compound **5**

41 ^{13}C NMR spectrum for compound **5**

42 DEPT (θ = 135°) spectrum for compound **5**

43 ^{1}H-^{1}H COSY spectrum for compound **5**

44 HMQC spectrum for compound **5**

45 HMBC spectrum for compound **5**

46 Noesy spectrum for compound **5**

47 HR-ESI-MS spectrum for compound **6**

48 IR spectrum for compound **6**

49 ^{1}H NMR spectrum for compound **6**

50 ^{13}C NMR spectrum for compound **6**

51 DEPT (θ = 135°) spectrum for compound **6**

52 ^{1}H-^{1}H COSY spectrum for compound **6**

53 HMQC spectrum for compound **6**

54 HMBC spectrum for compound **6**

55 HR-ESI-MS spectrum for compound **7**

56 IR spectrum for compound **7**

57 ^{1}H NMR spectrum for compound **7**

58 ^{13}C NMR spectrum for compound **7**

59 DEPT (θ = 135°) spectrum for compound **7**

60 ^{1}H-^{1}H COSY spectrum for compound **7**

61 HMQC spectrum for compound **7**

62 HMBC spectrum for compound **7**

63 Noesy spectrum for compound **7**

64 HR-ESI-MS spectrum for compound **8**

65 IR spectrum for compound **8**

66 ^{1}H NMR spectrum for compound **8**

67 ^{13}C NMR spectrum for compound **8**

68 DEPT (θ = 135°) spectrum for compound **8**

69 ^{1}H-^{1}H COSY spectrum for compound **8**

70 HMQC spectrum for compound **8**

71 HMBC spectrum for compound **8**

72 Noesy spectrum for compound **8**

73 HR-ESI-MS spectrum for compound **9**

74 IR spectrum for compound **9**

75 ^{1}H NMR spectrum for compound **9**

76 ^{13}C NMR spectrum for compound **9**

77 DEPT (θ = 135°) spectrum for compound **9**

78 ^{1}H-^{1}H COSY spectrum for compound **9**

79 HMQC spectrum for compound **9**

80 HMBC spectrum for compound **9**

81 Noesy spectrum for compound **9**

82 The exclusion experiment of the possibility of an artifact about anthriscifolsine A.

1 HR-ESI-MS spectrum for compound **1**

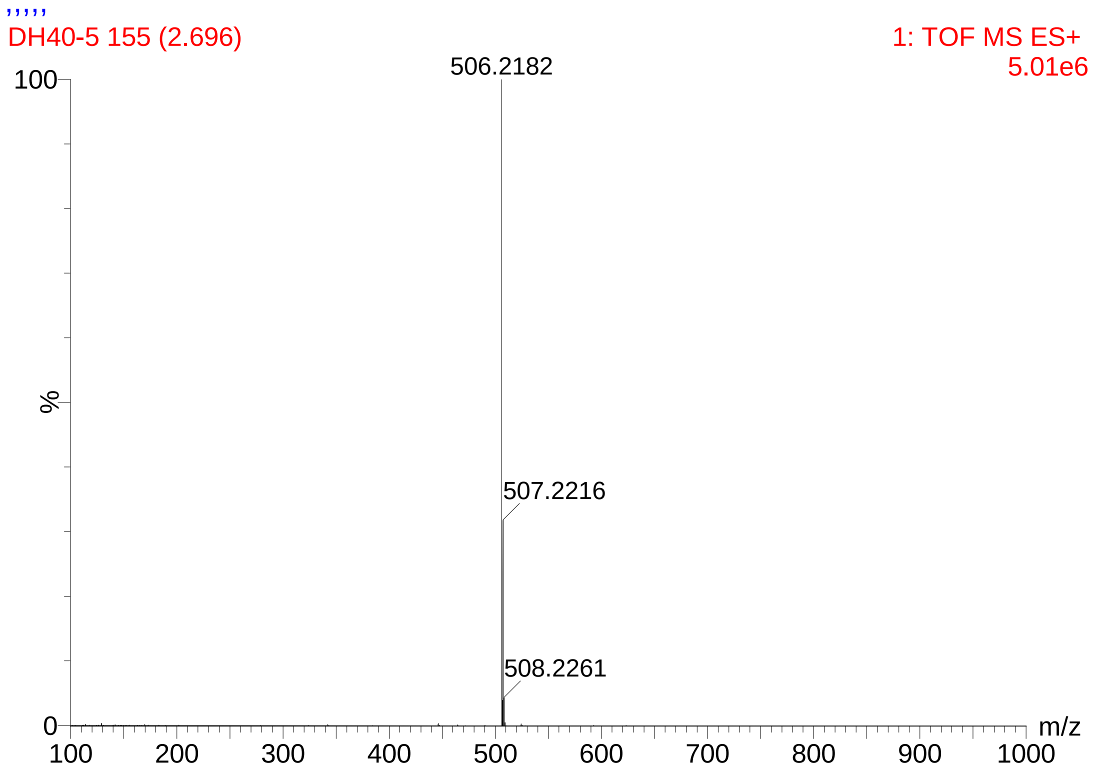

2 IR spectrum for compound **1**

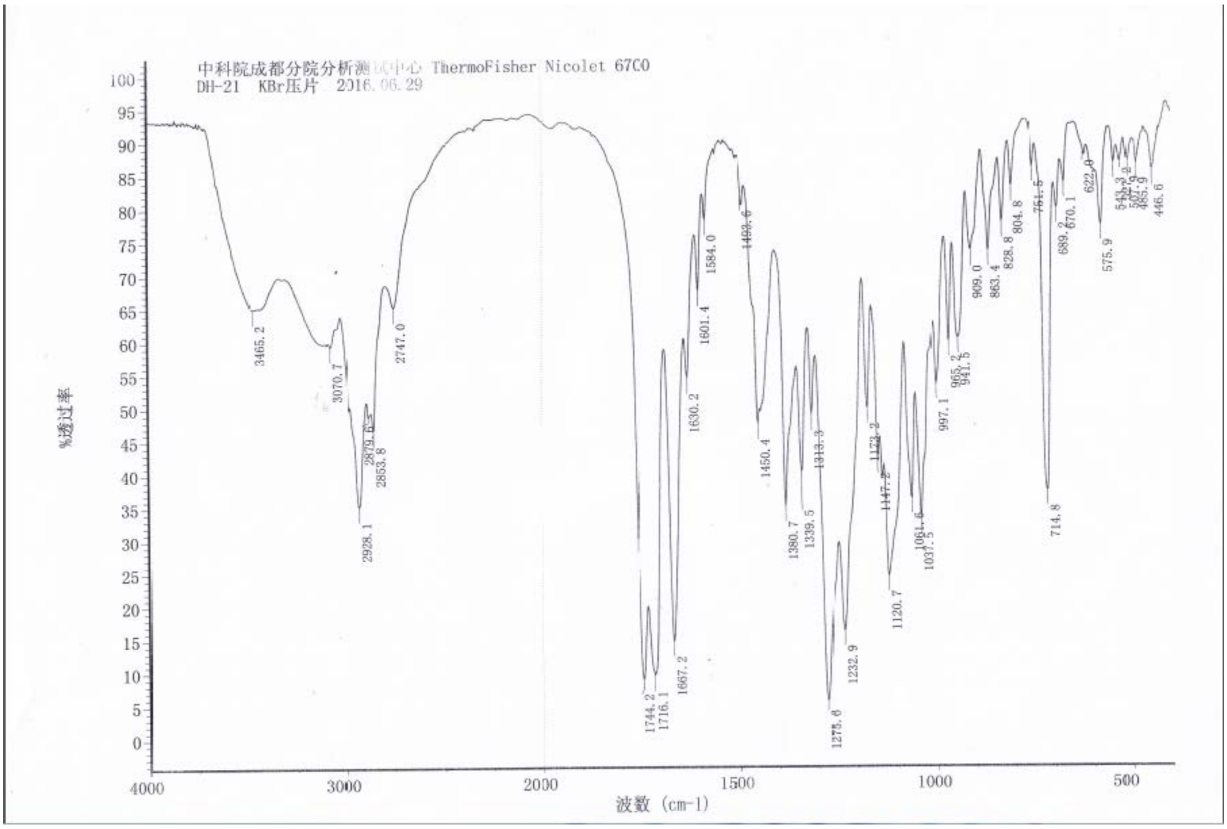

3 ^{1}H NMR spectrum for compound **1**

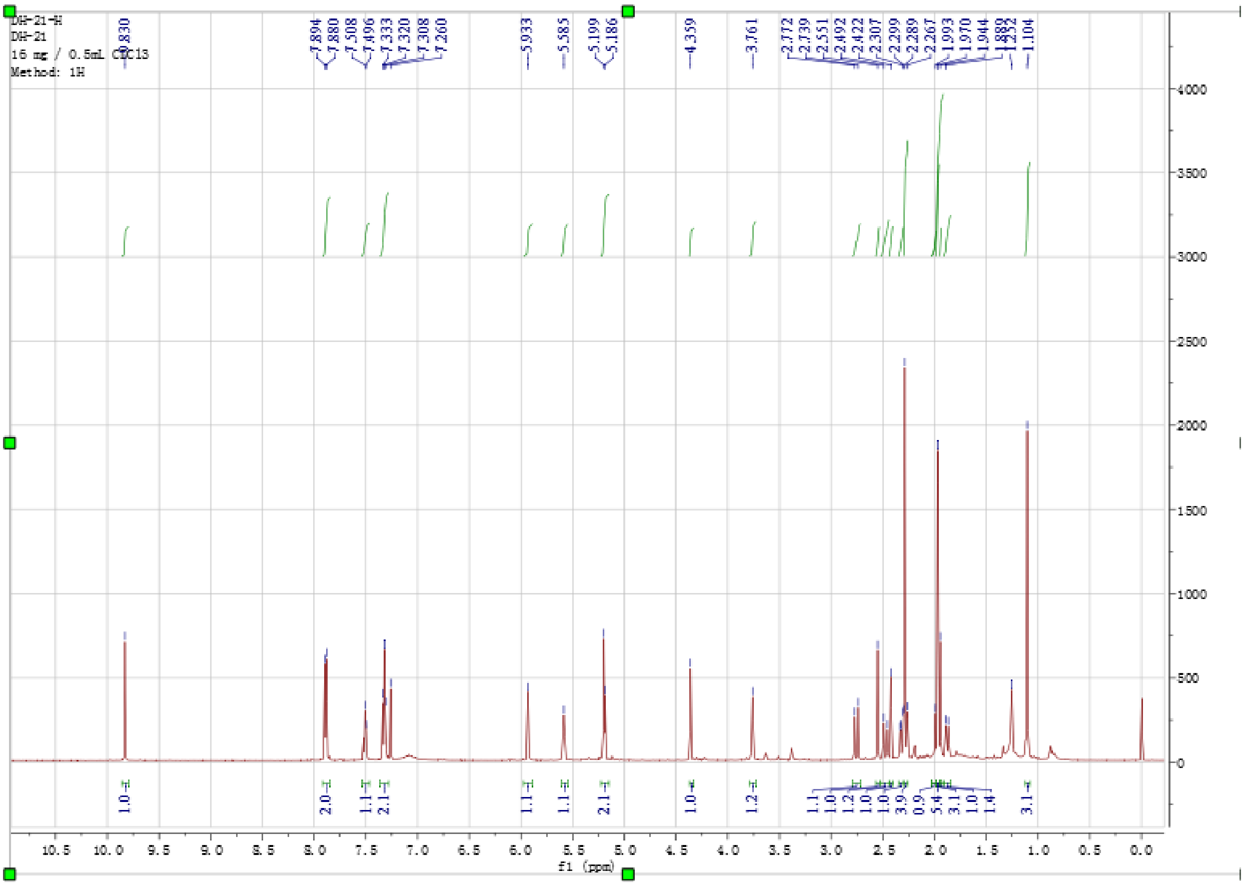

4 ^{13}C NMR spectrum for compound **1**

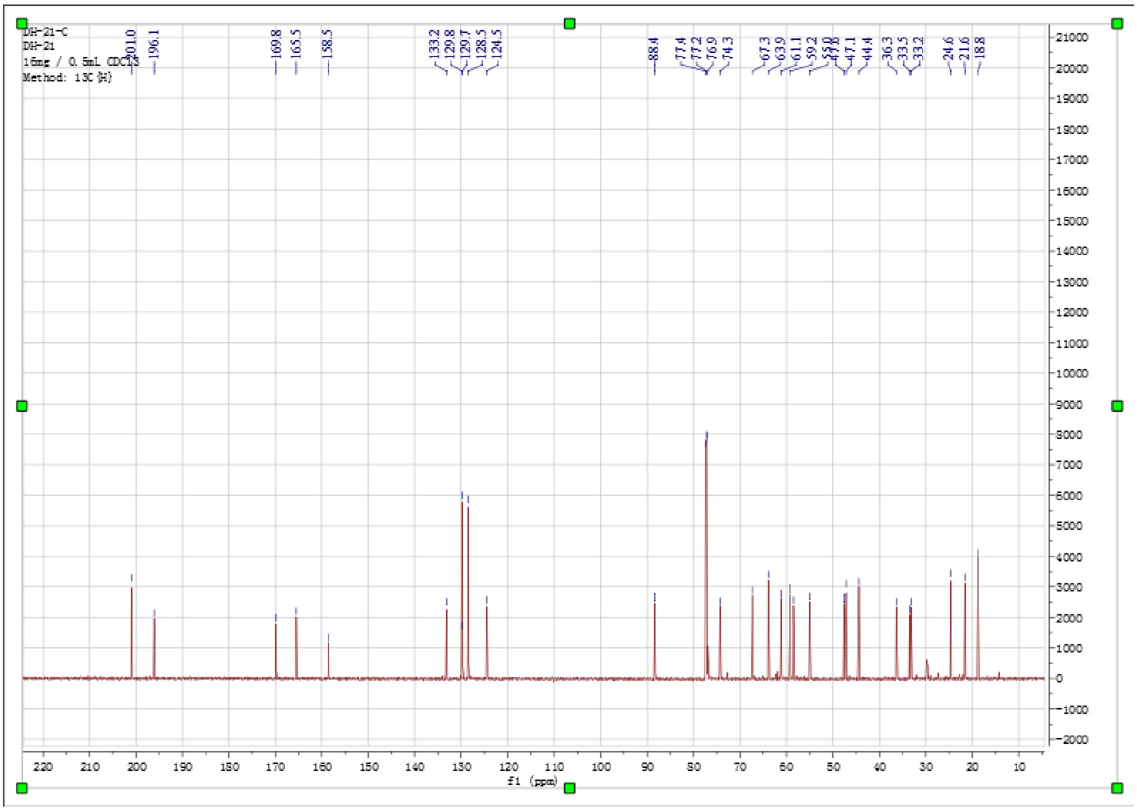

5 DEPT (θ = 135°) spectrum for compound **1**

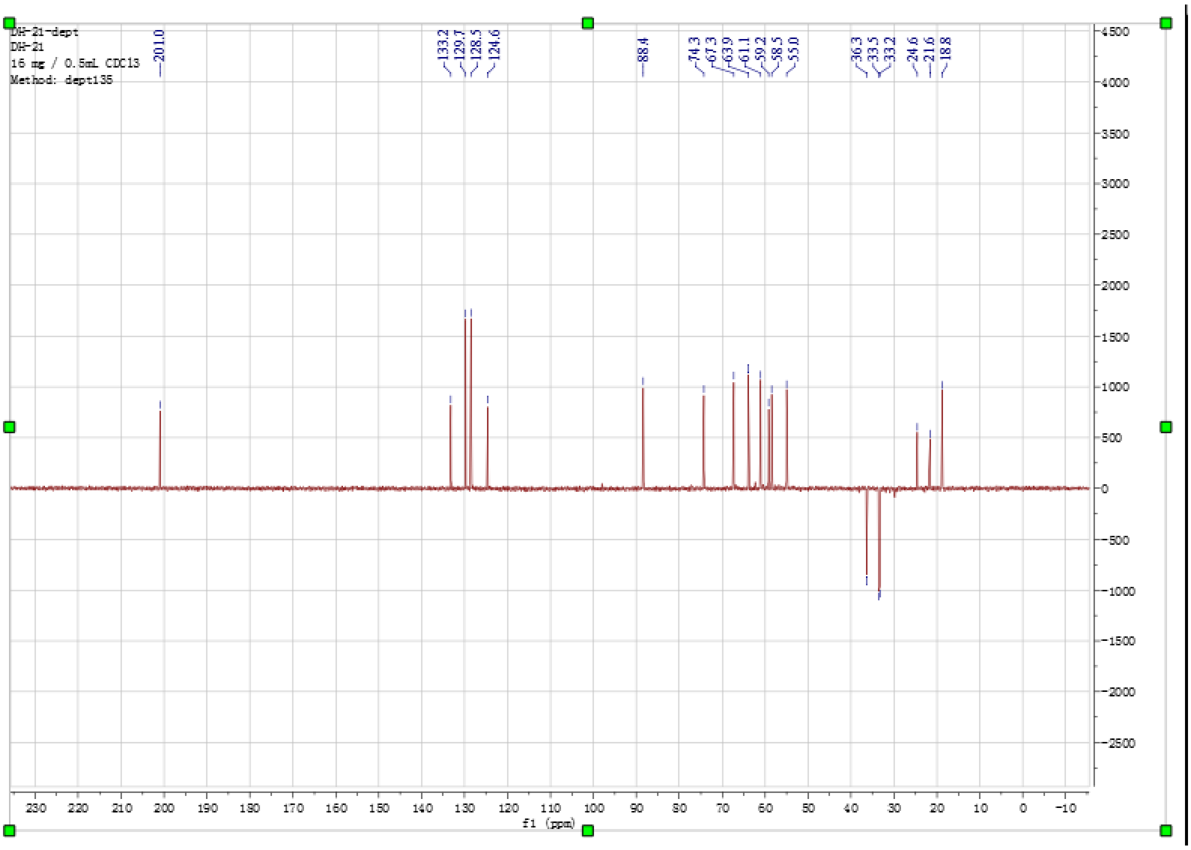

6 ^{1}H-^{1}H COSY spectrum for compound **1**

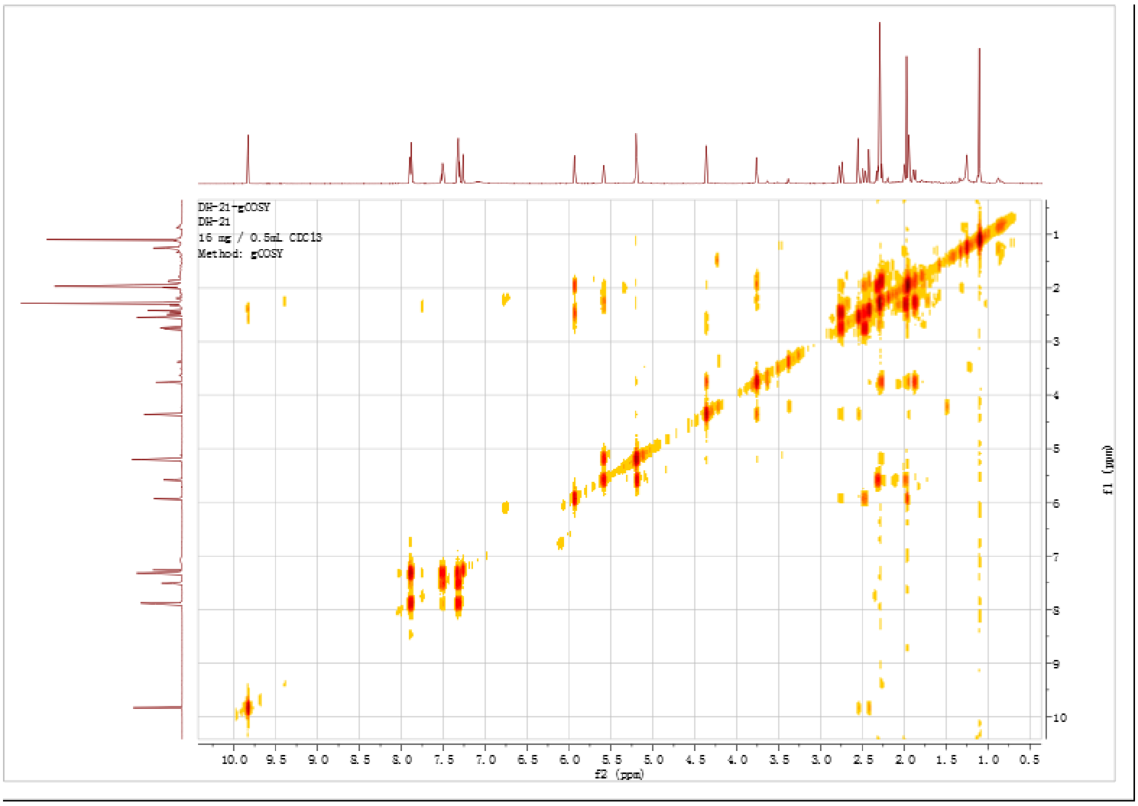

7 HMQC spectrum for compound **1**

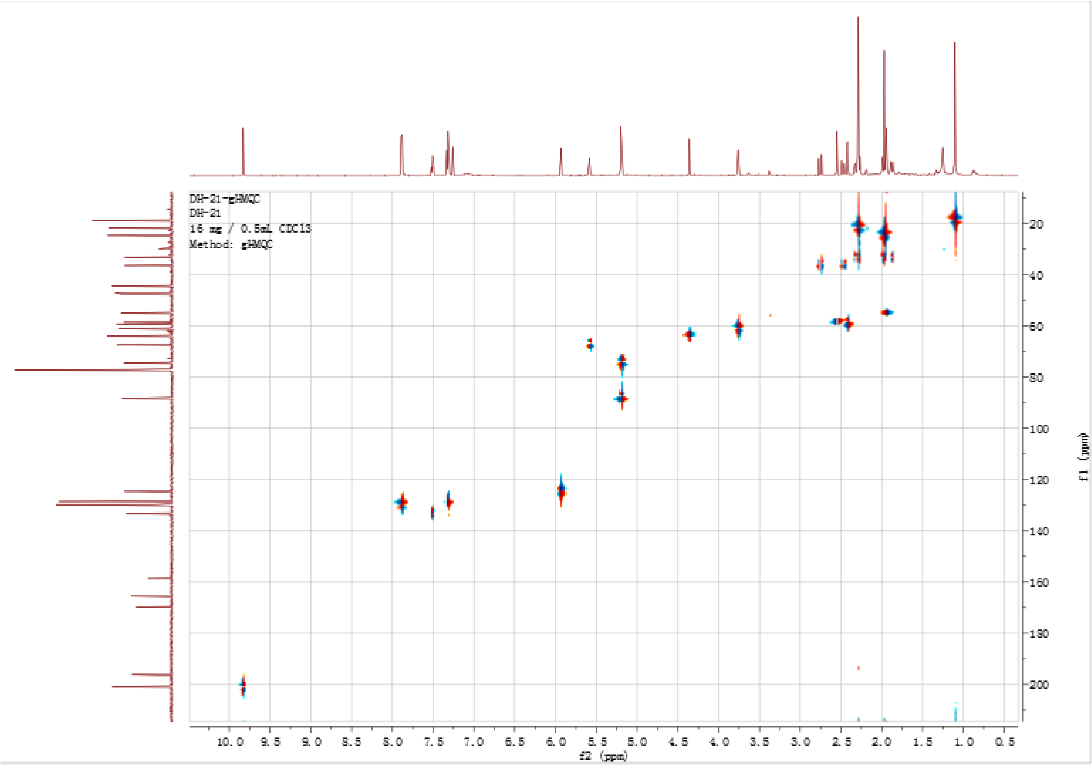

8 HMBC spectrum for compound **1**

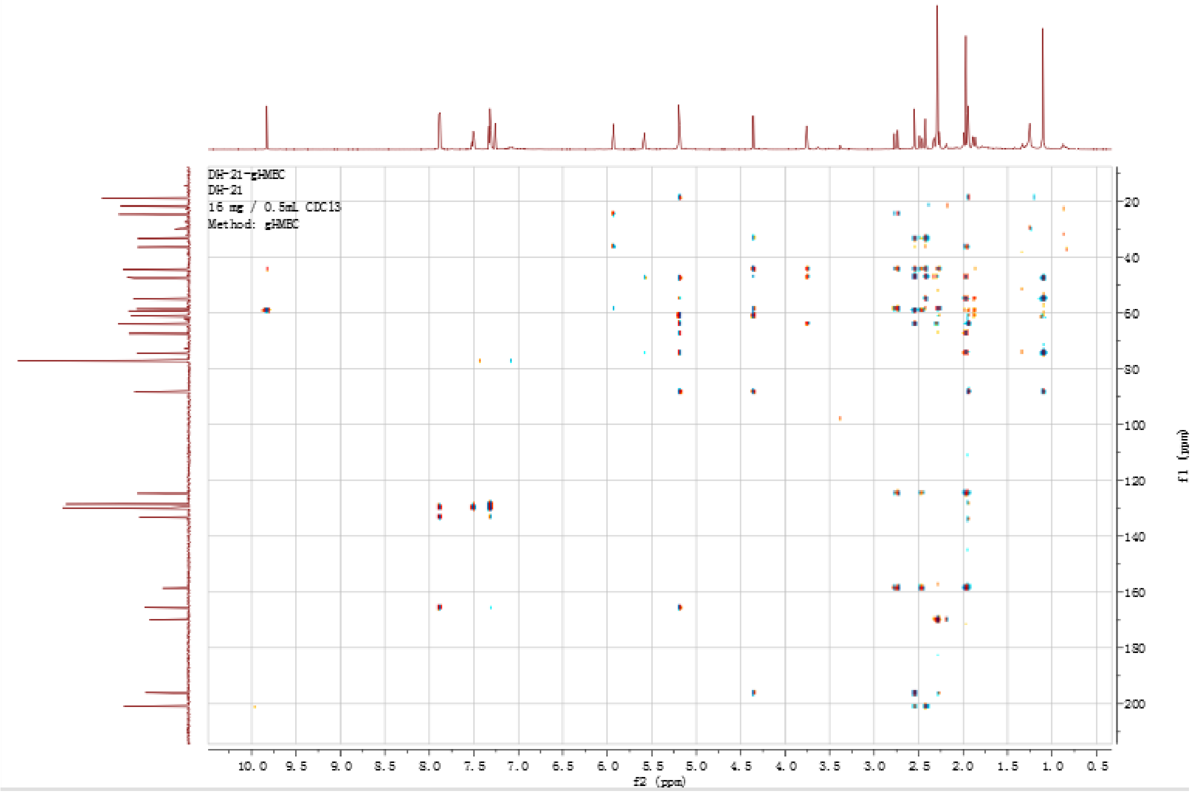

9 Noesy spectrum for compound **1**

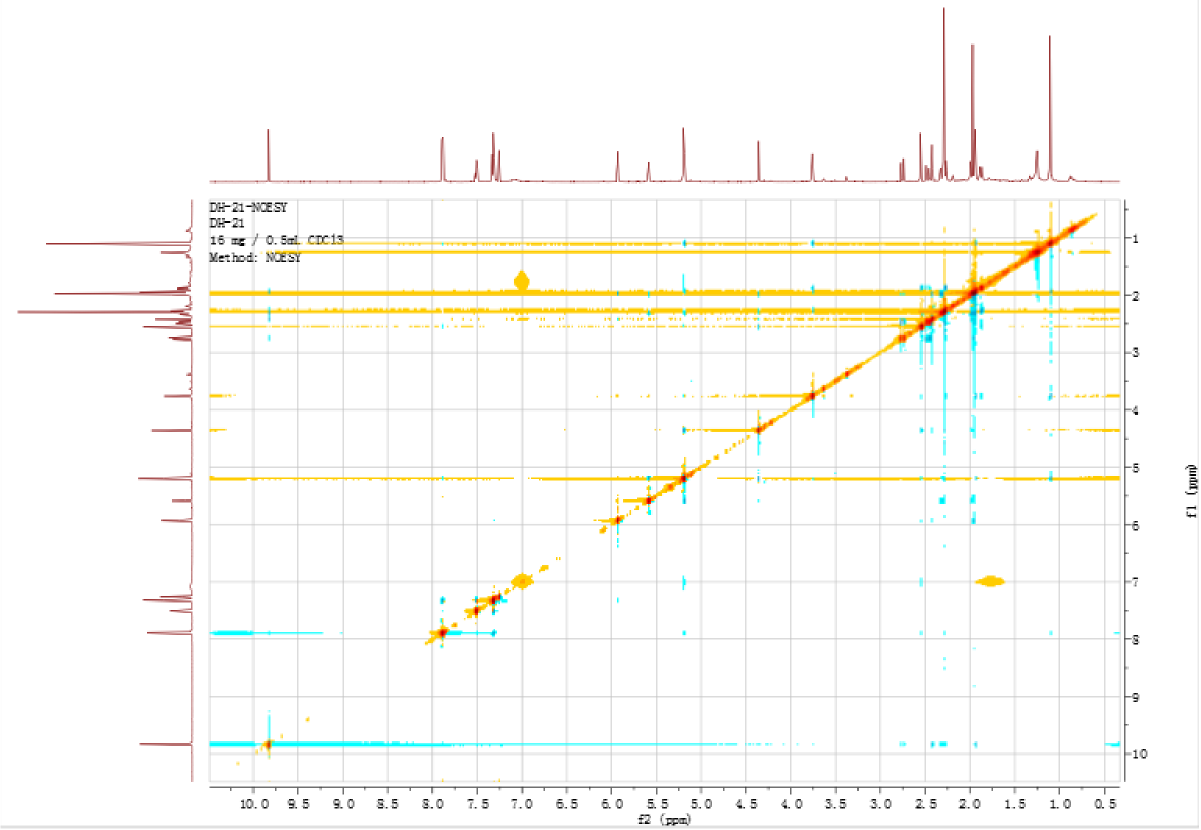

10 X-Ray spectrum for compound **1**

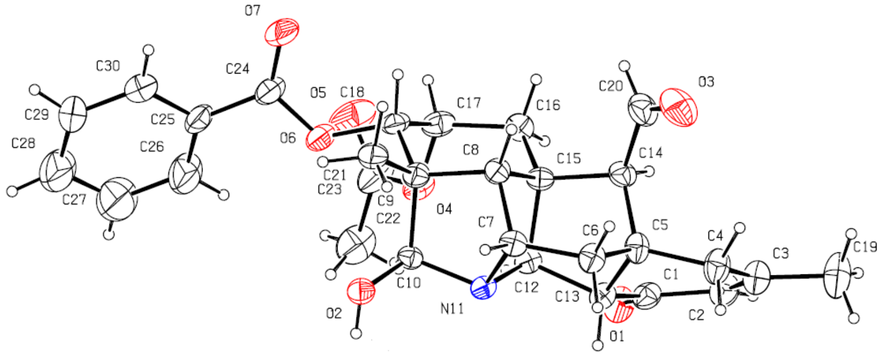

11 HR-ESI-MS spectrum for compound **2**

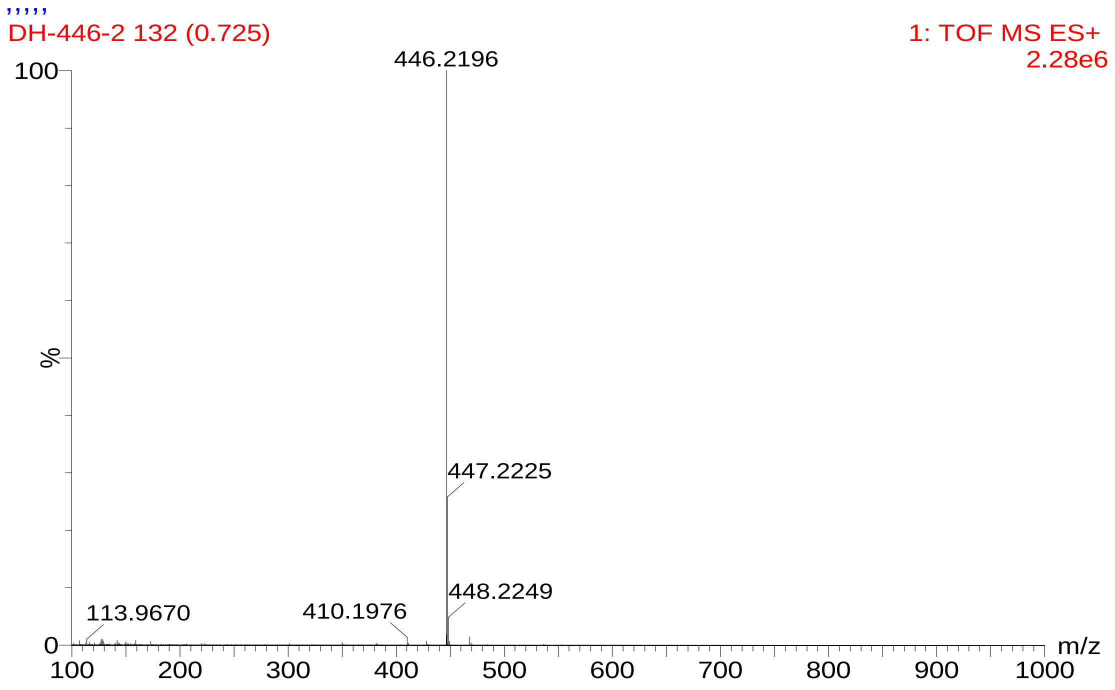

12 IR spectrum for compound **2**

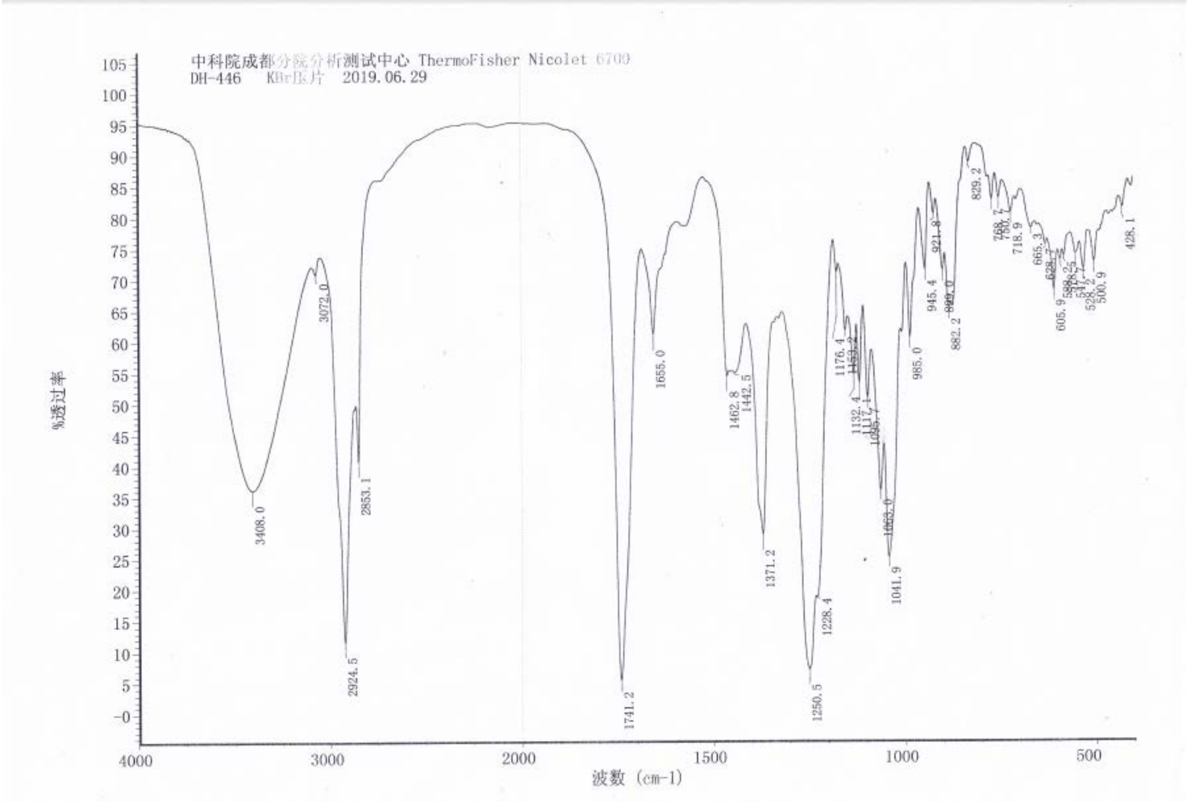

13 ^{1}H NMR spectrum for compound **2**

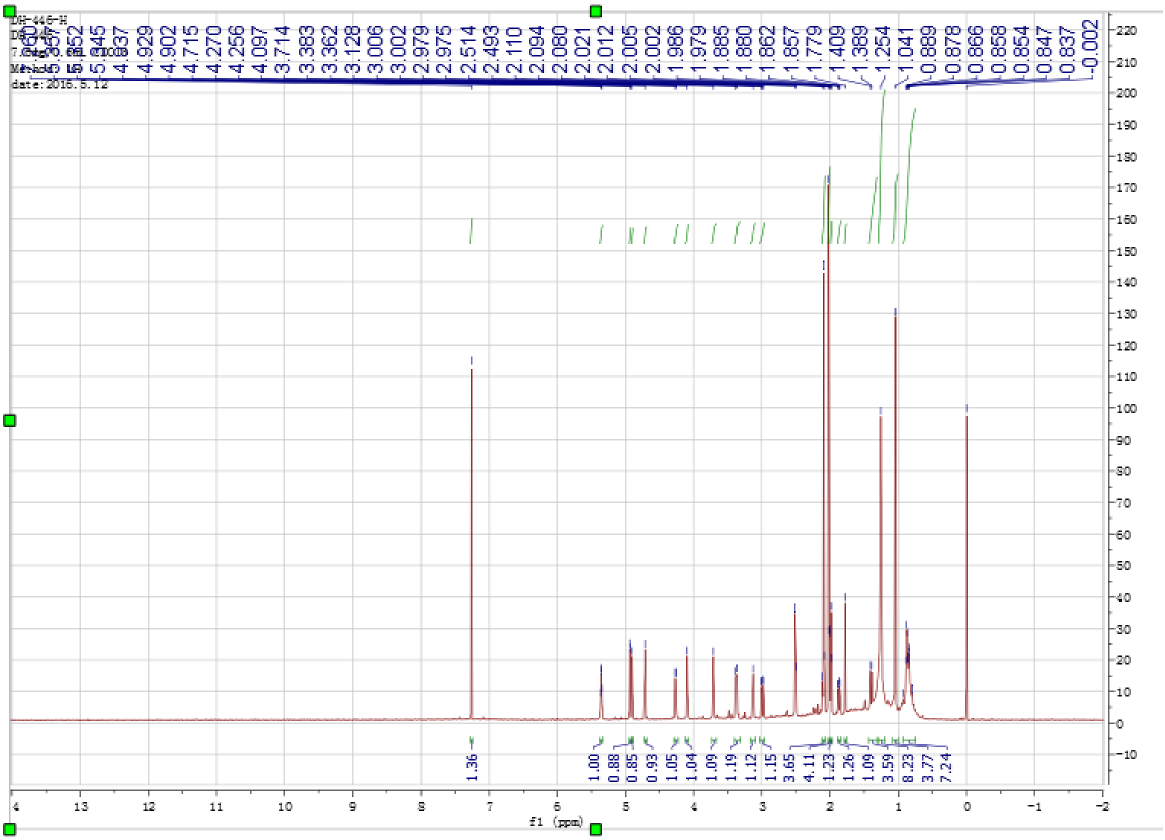

14 ^{13}C NMR spectrum for compound **2**

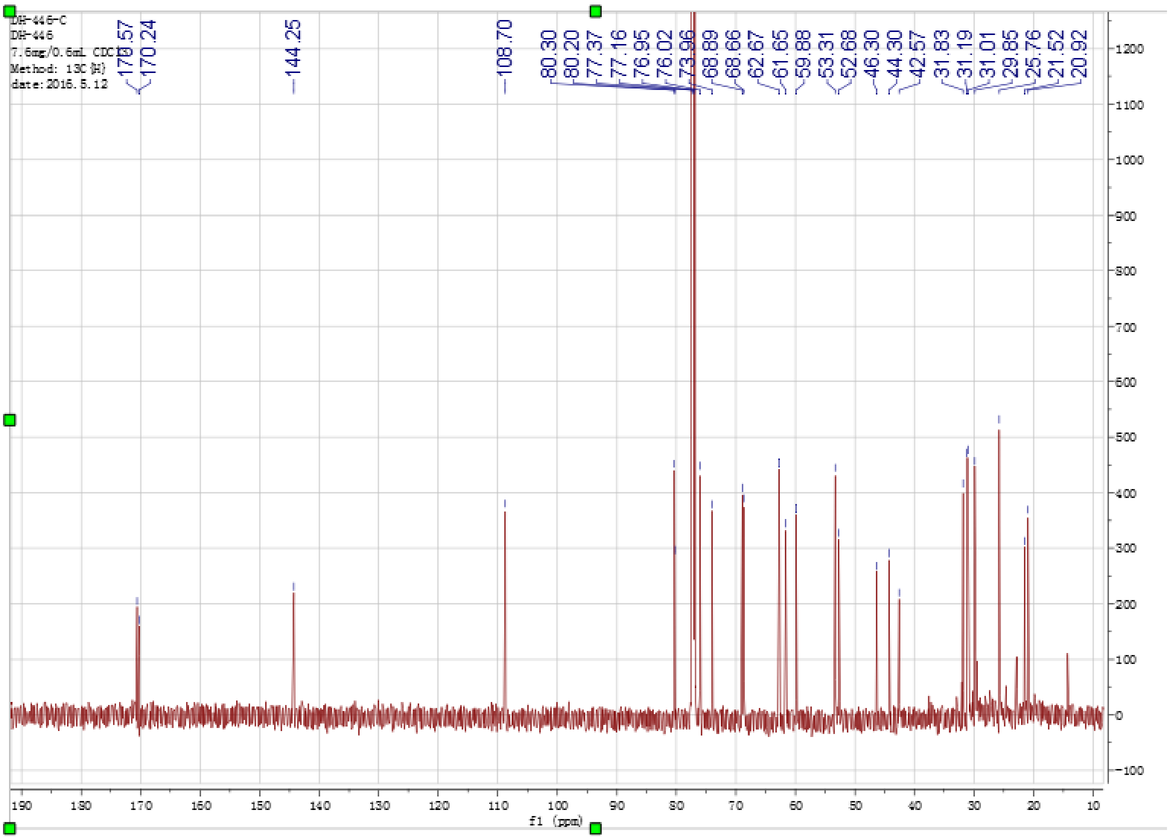

15 DEPT ($\theta = 135°$) spectrum for compound **2**

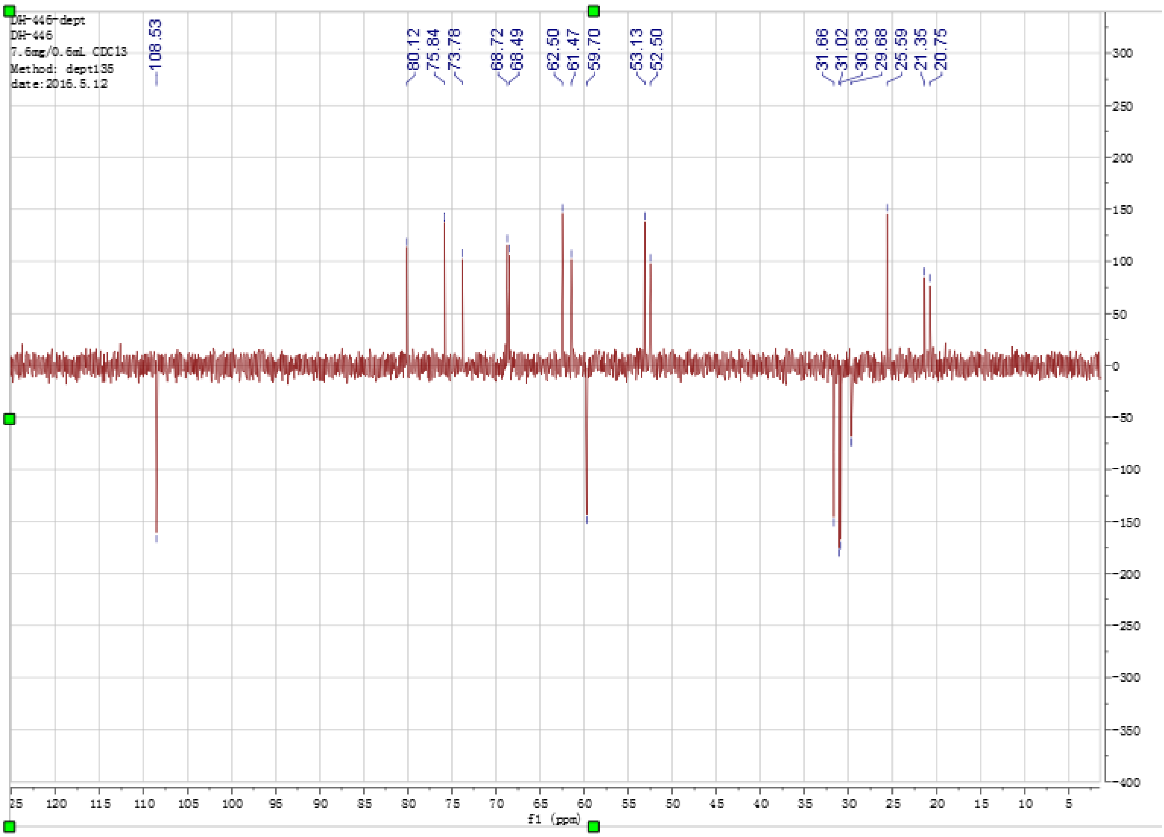

16 ^{1}H-^{1}H COSY spectrum for compound **2**

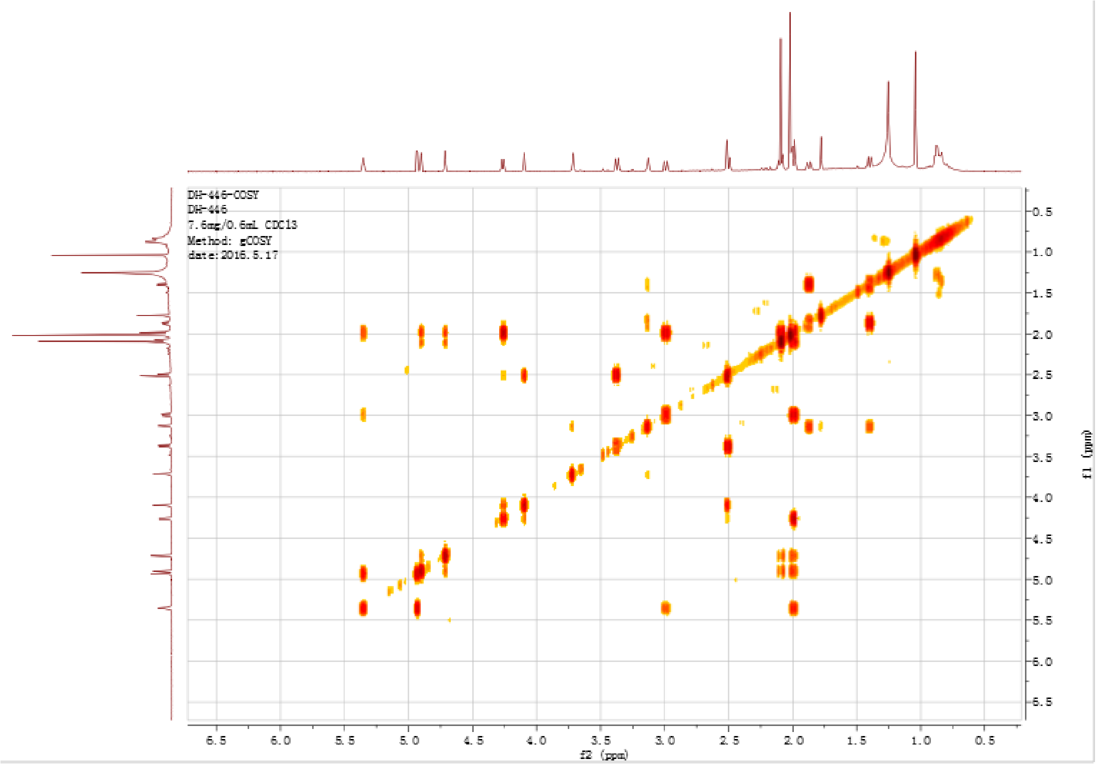

17 HMQC spectrum for compound **2**

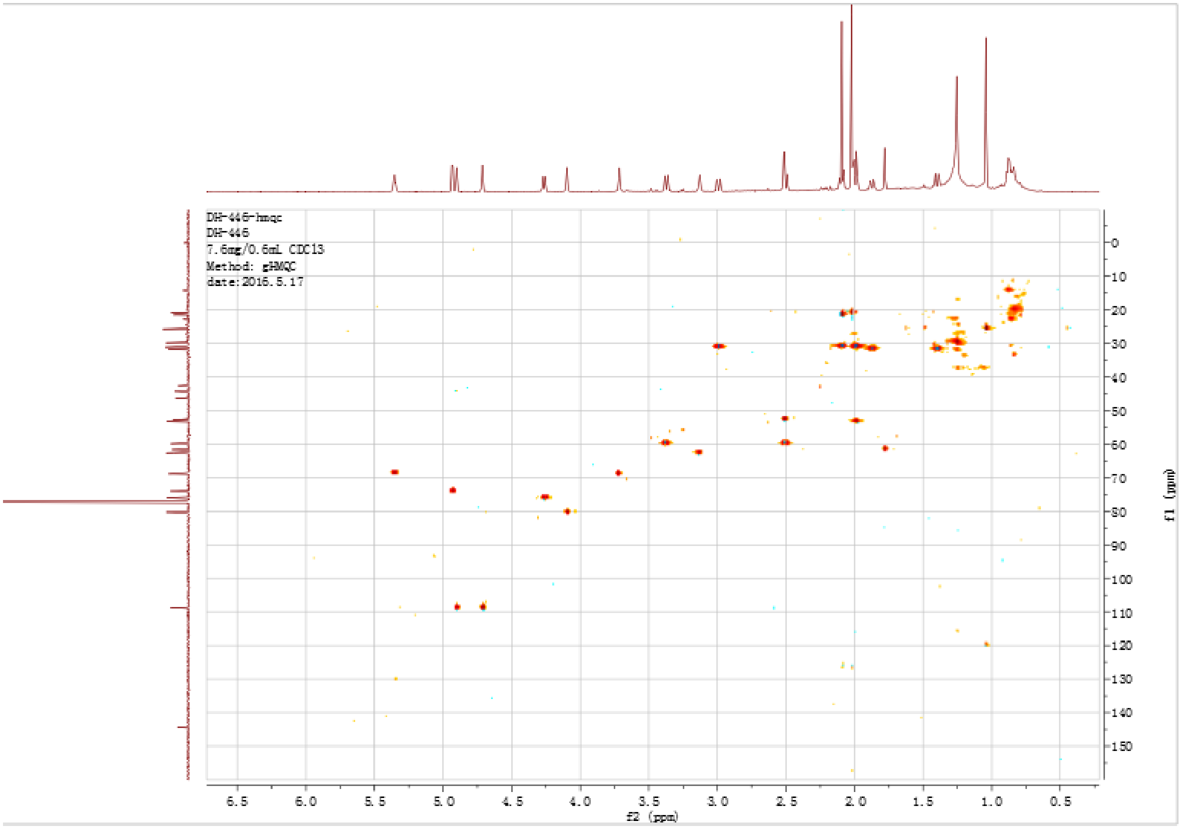

18 HMBC spectrum for compound **2**

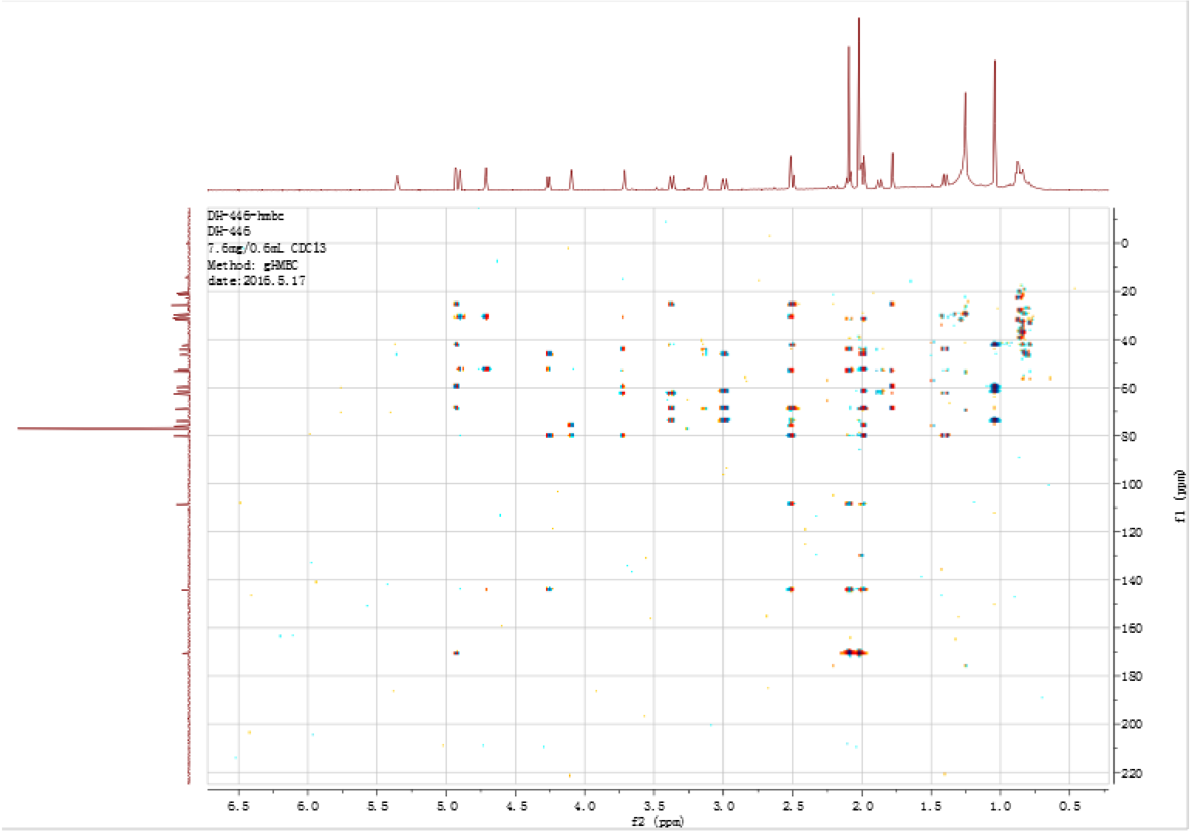

19 Noesy spectrum for compound **2**

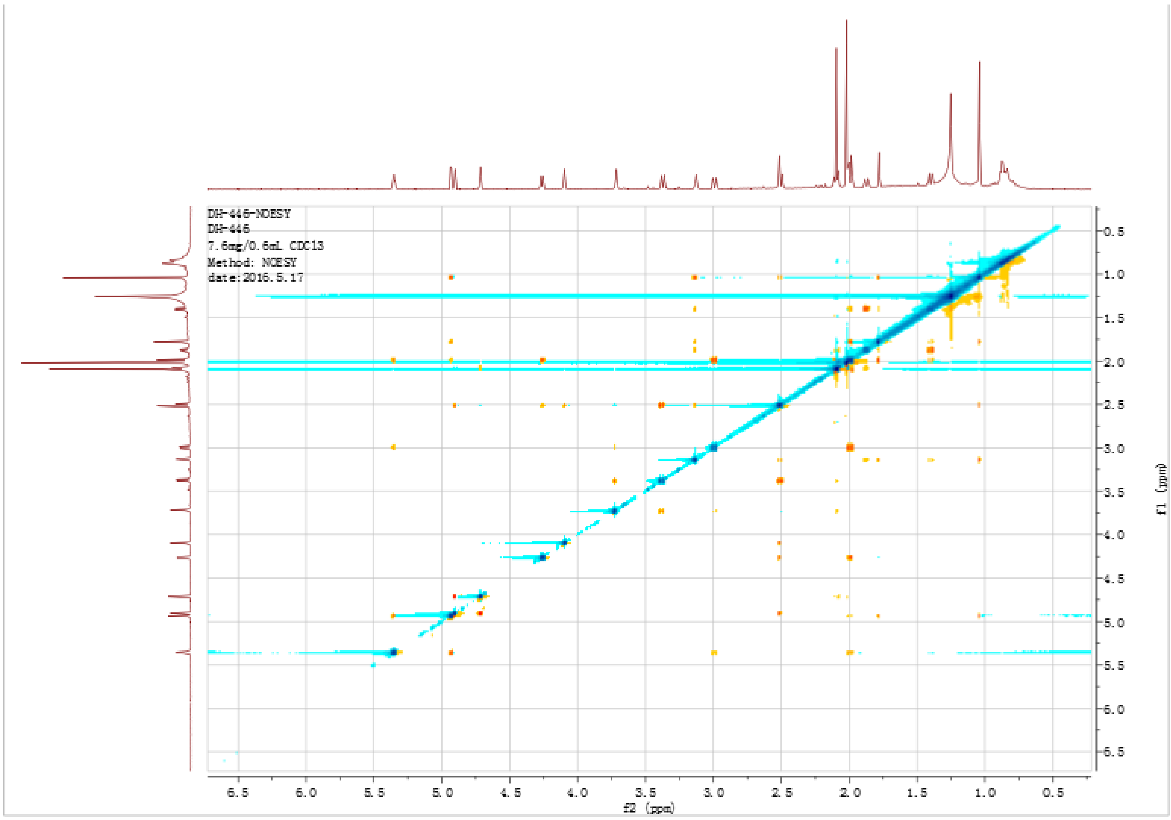

20 HR-ESI-MS spectrum for compound **3**

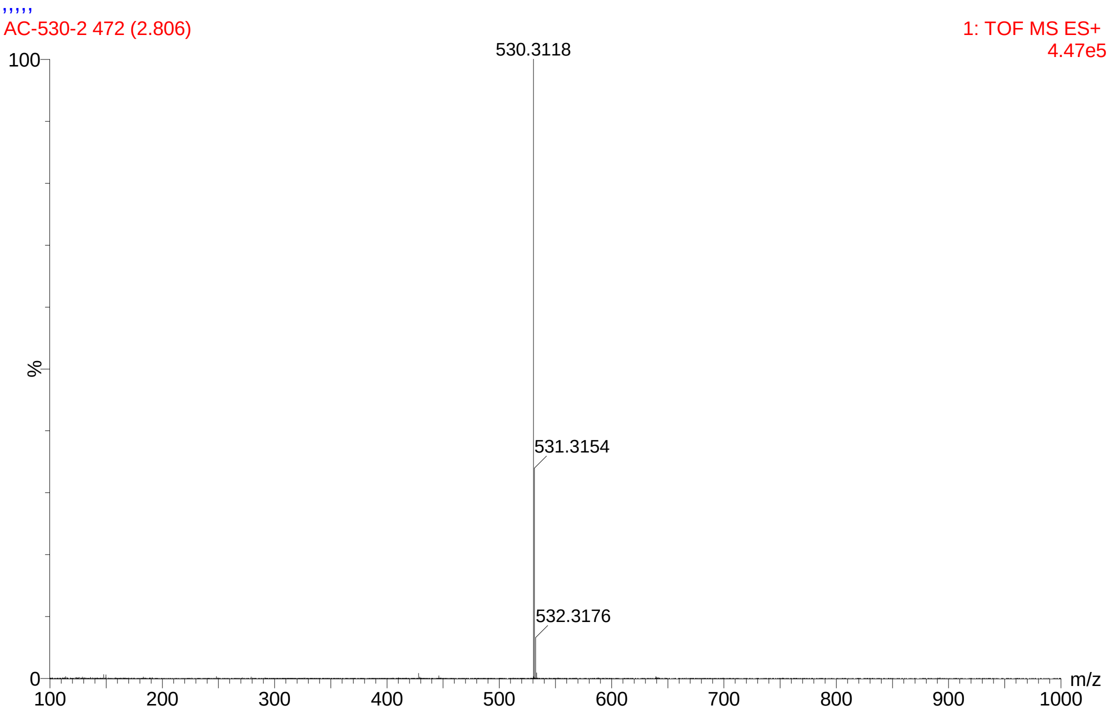

21 IR spectrum for compound **3**

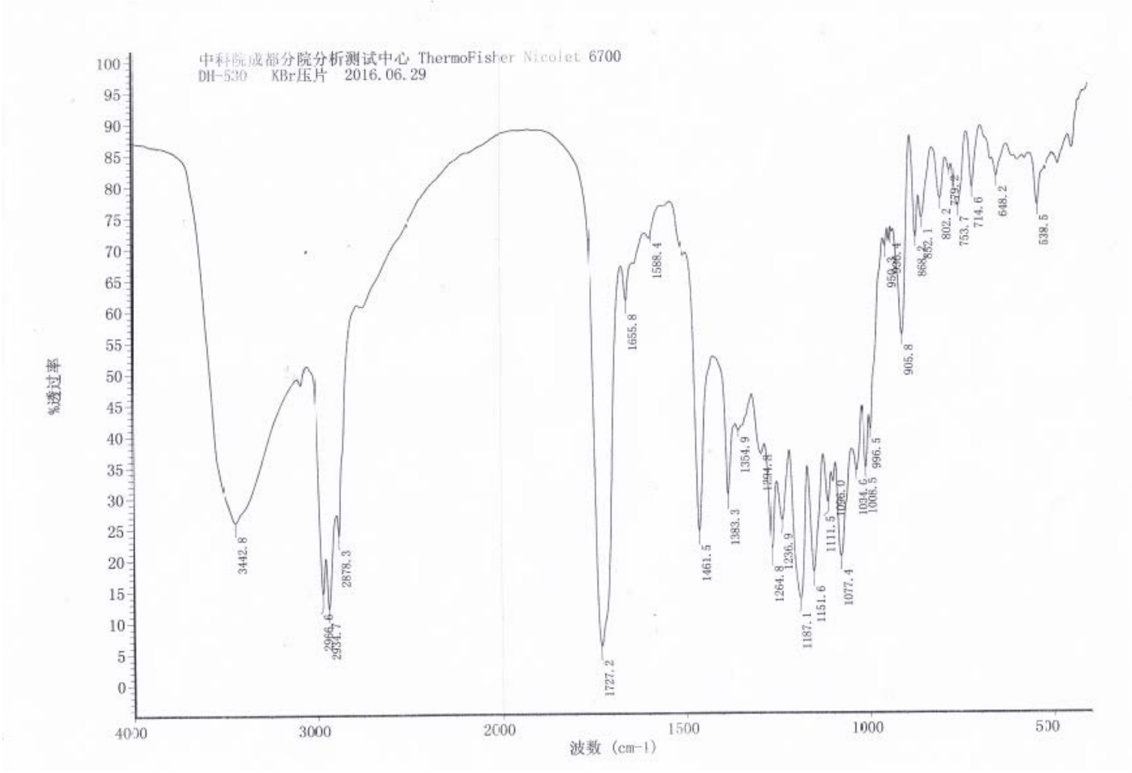

22 ^{1}H NMR spectrum for compound **3**

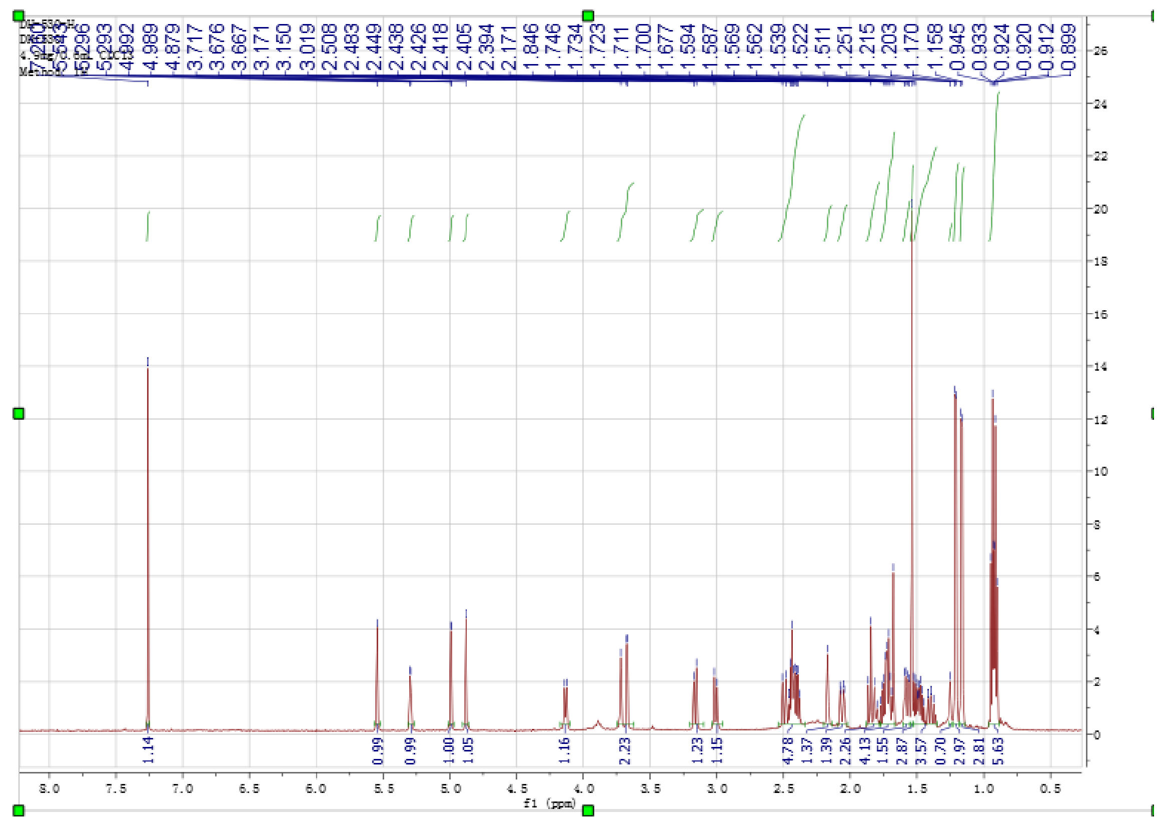

23 ^{13}C NMR spectrum for compound **3**

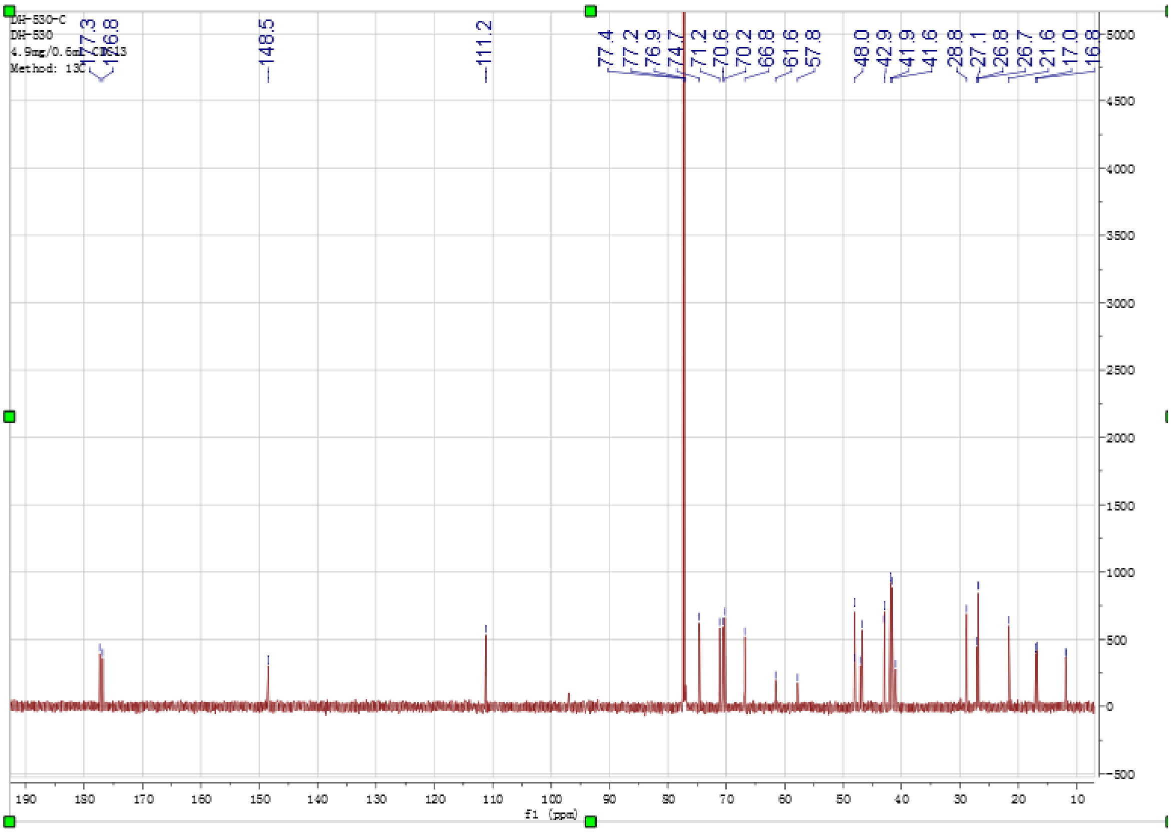

24 DEPT ($\theta = 135^{\circ}$) spectrum for compound **3**

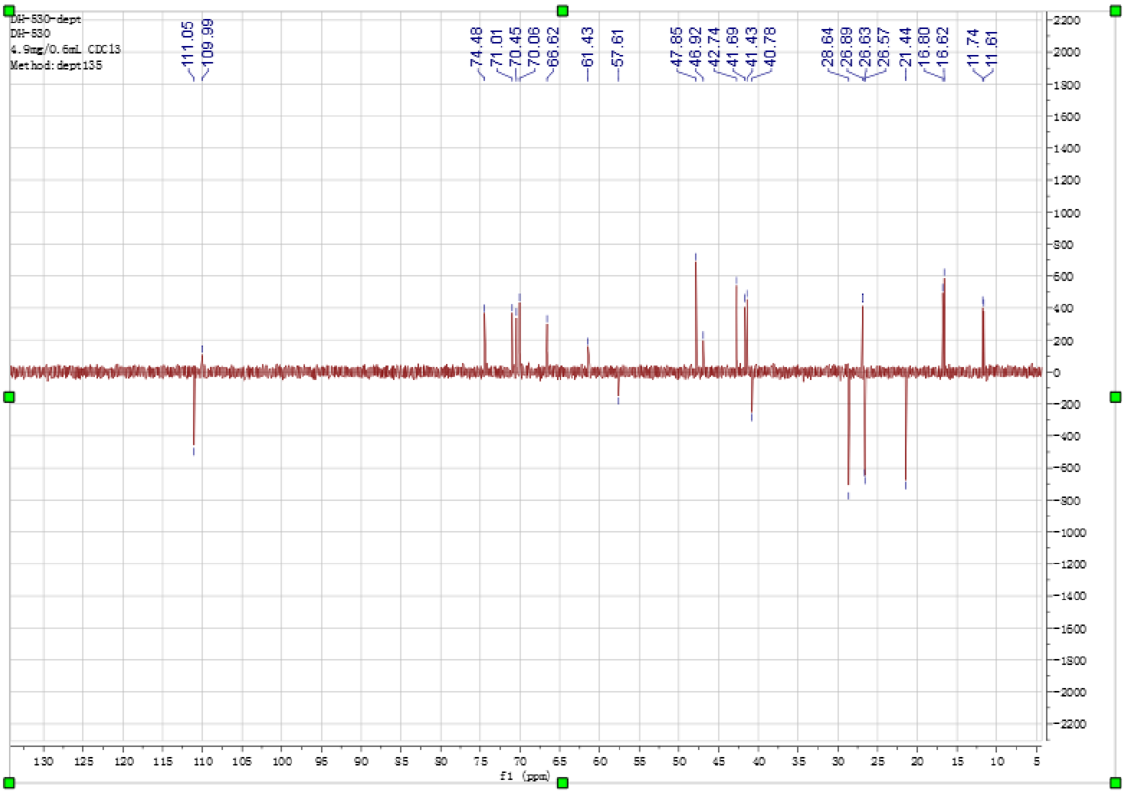

25 ^{1}H-^{1}H COSY spectrum for compound **3**

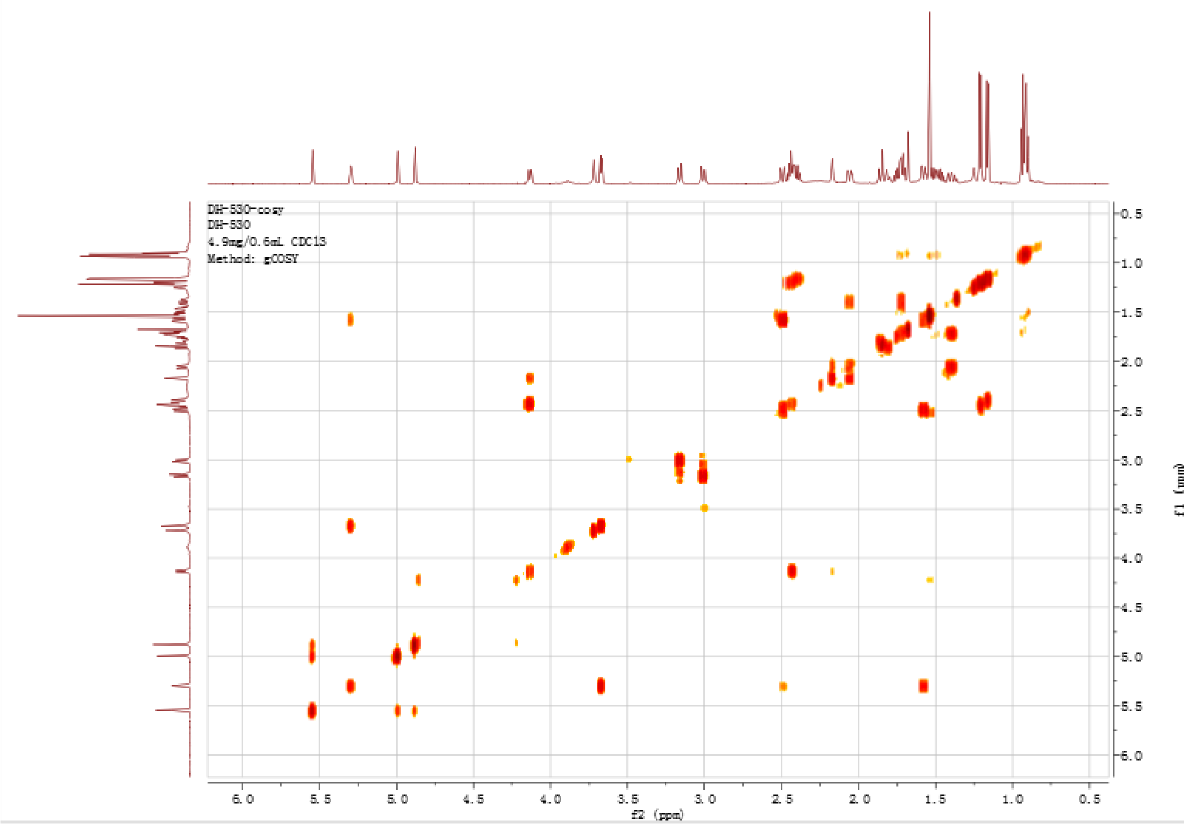

26 HMQC spectrum for compound **3**

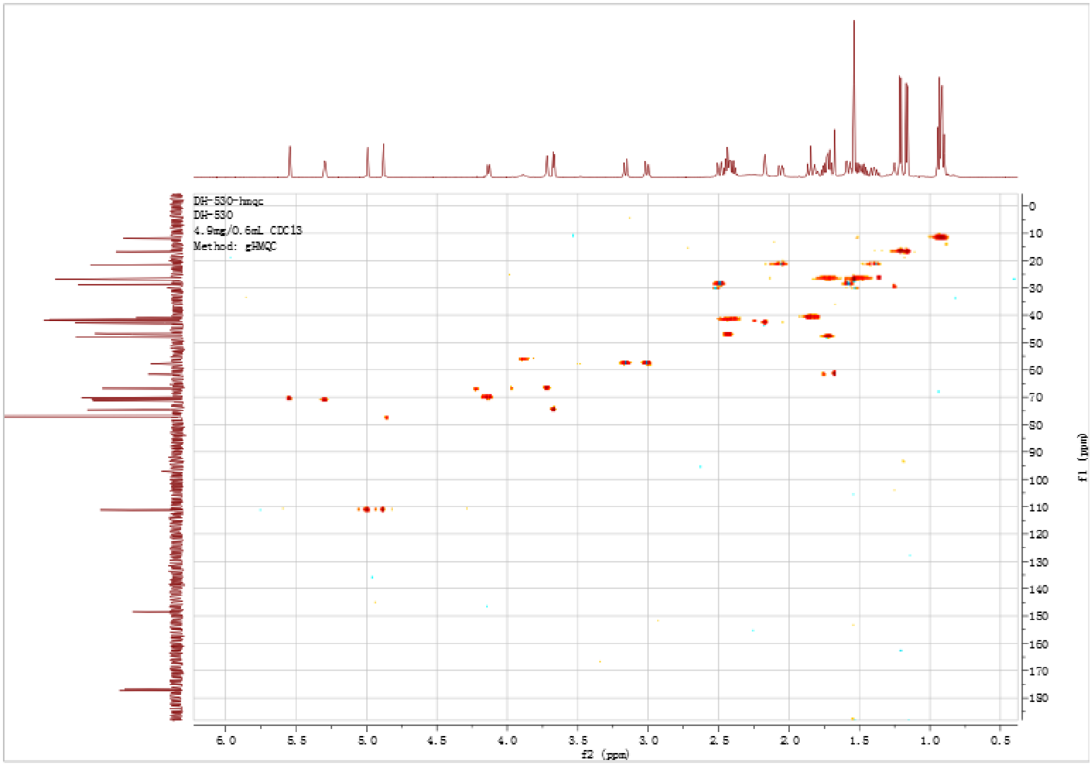

27 HMBC spectrum for compound **3**

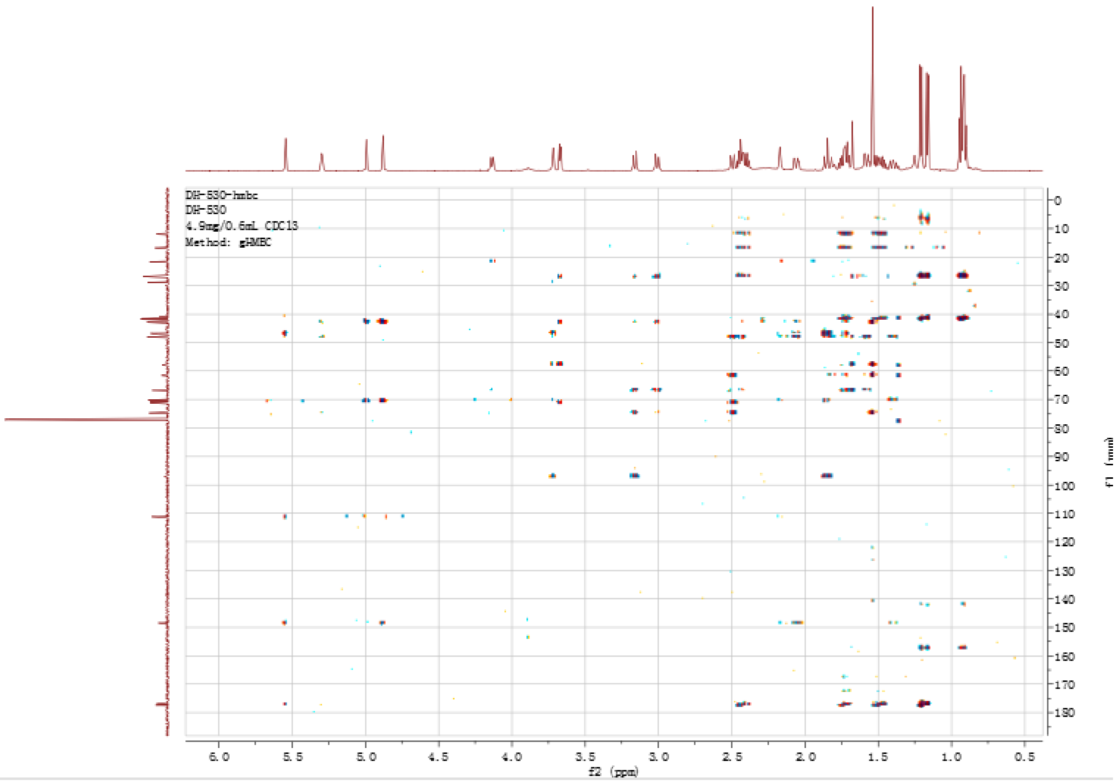

28 Noesy spectrum for compound **3**

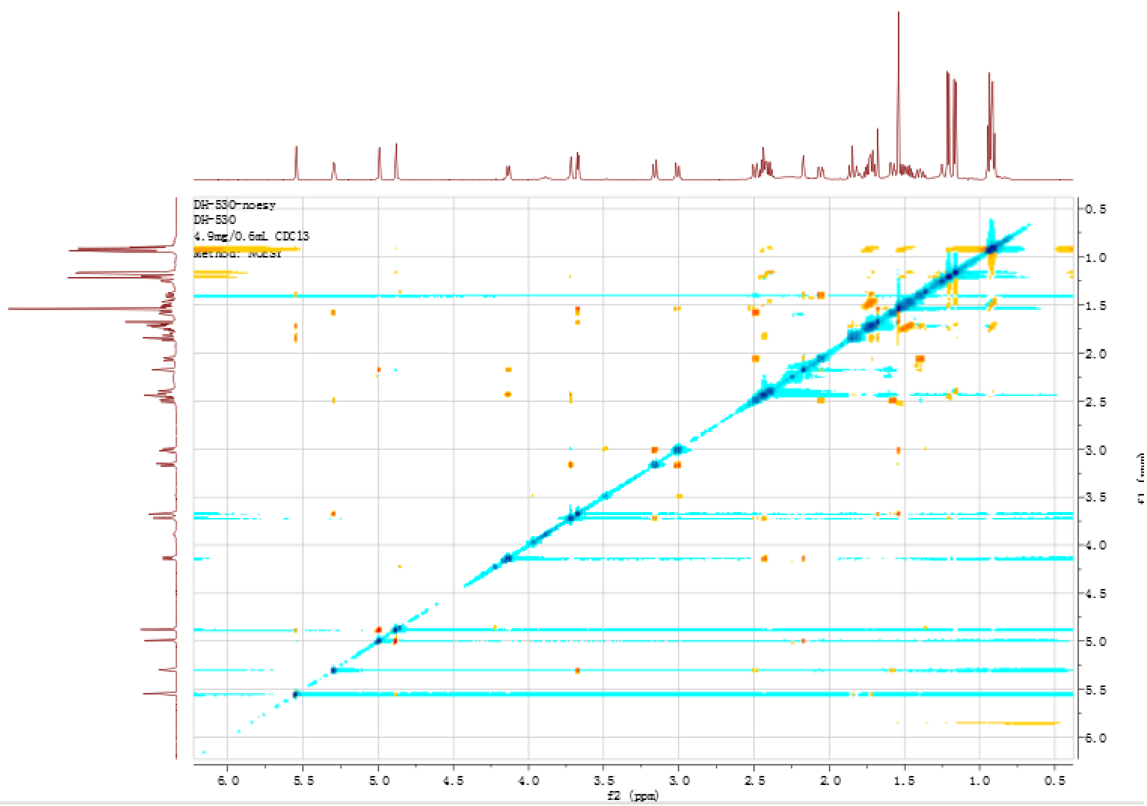

29 HR-ESI-MS spectrum for compound **4**

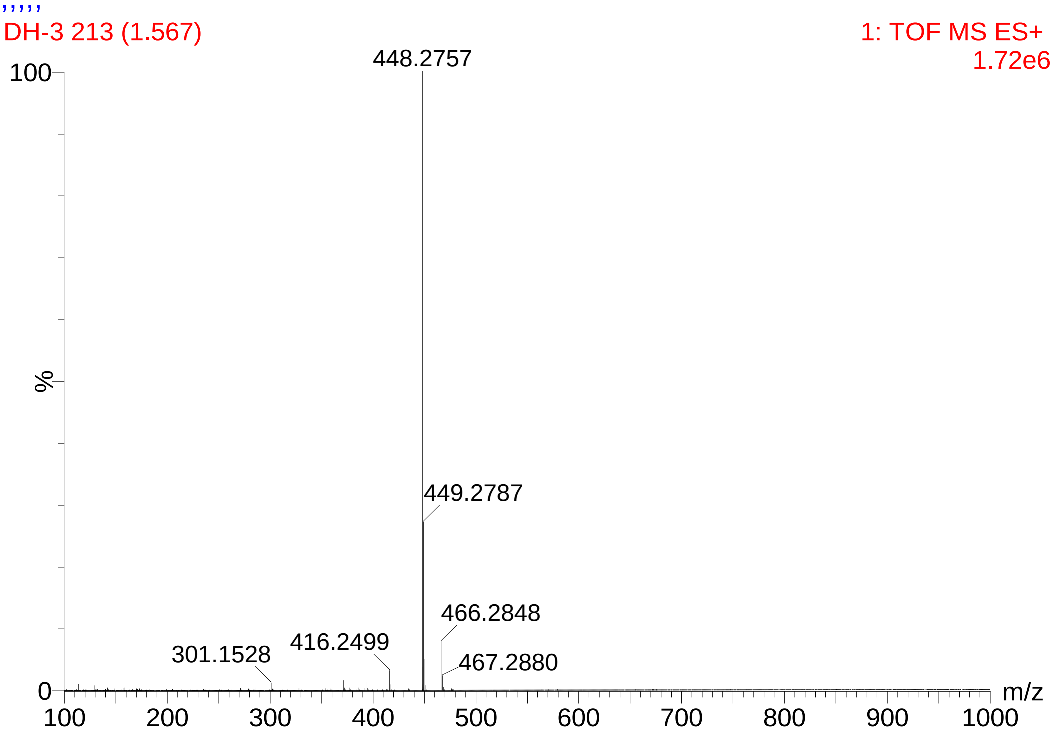

30 IR spectrum for compound **4**

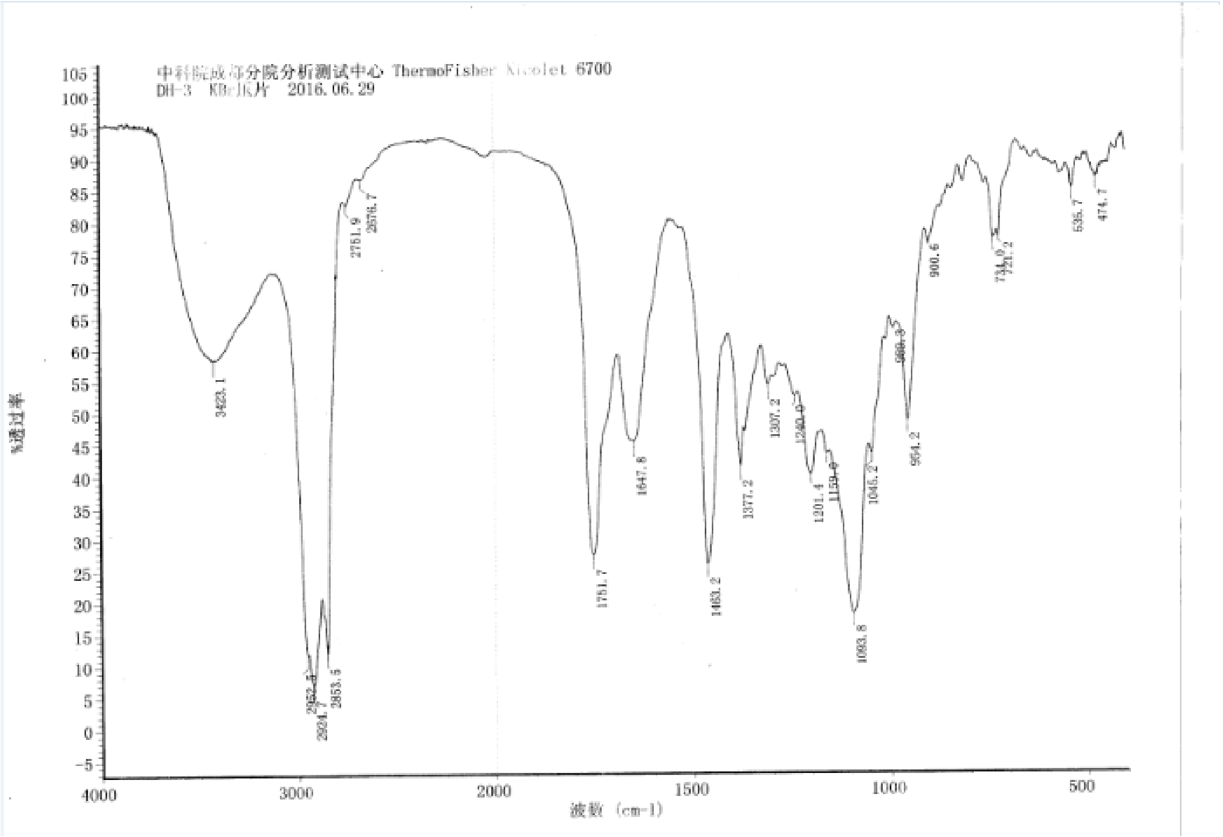

31 ¹H NMR spectrum for compound **4**

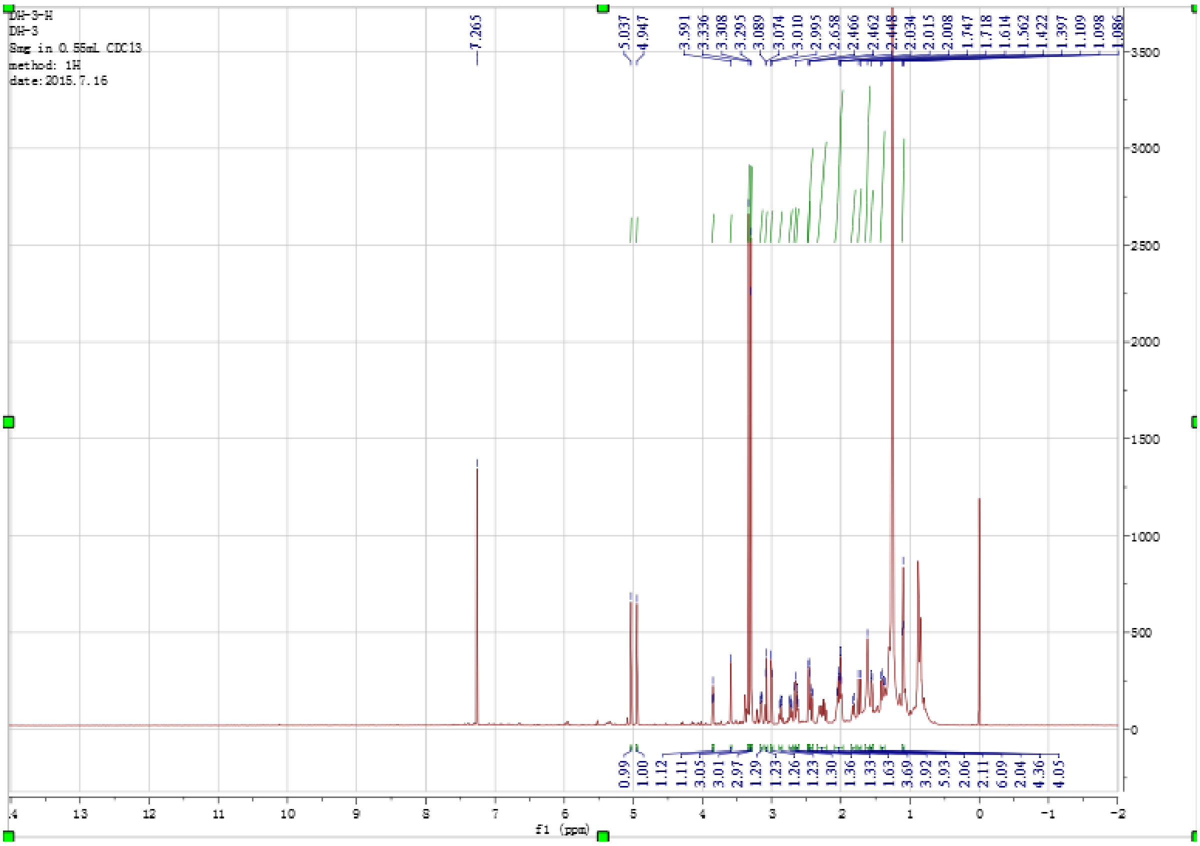

32 ¹³C NMR spectrum for compound **4**

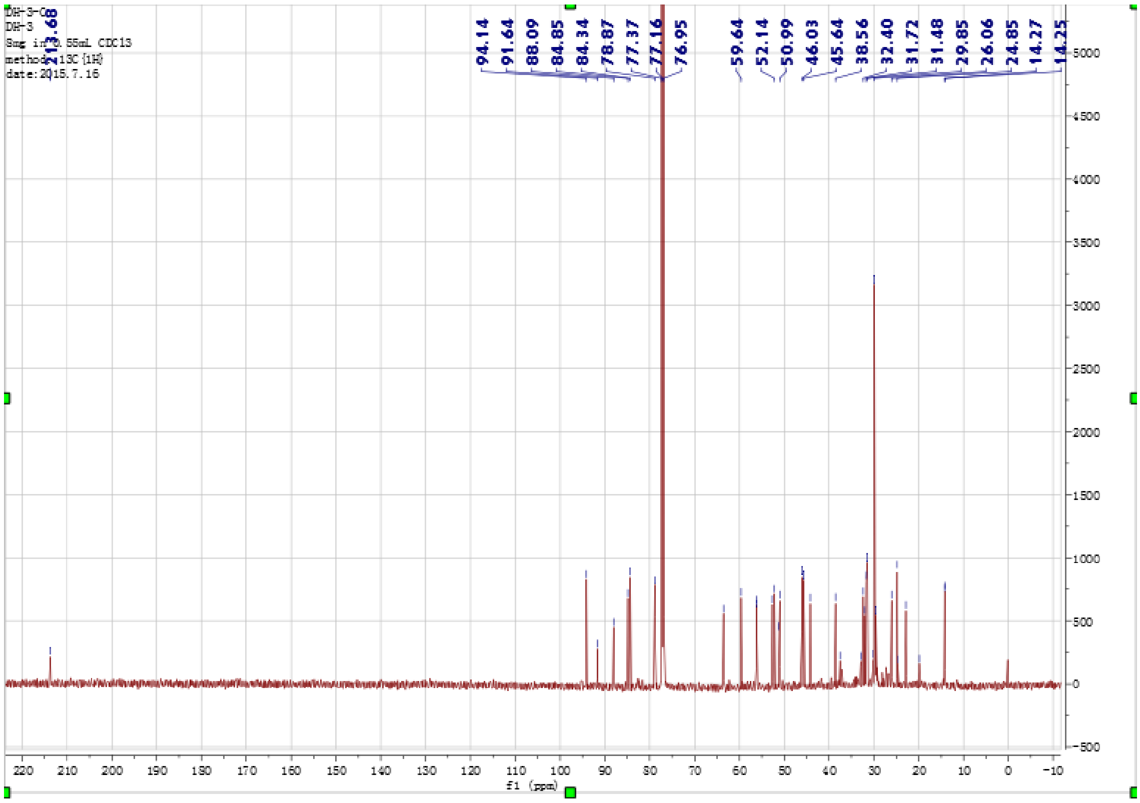

33 DEPT ($\theta = 135^{\circ}$) spectrum for compound **4**

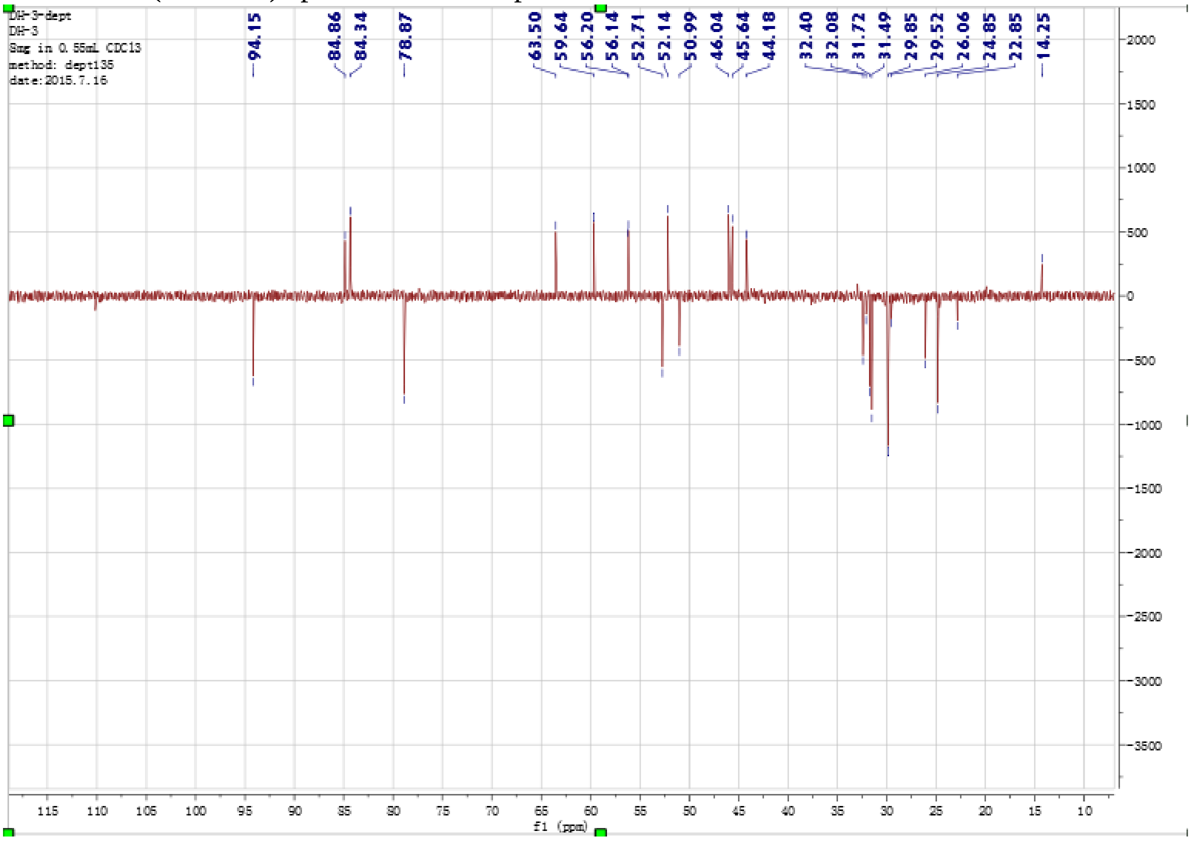

34 ^{1}H-^{1}H COSY spectrum for compound **4**

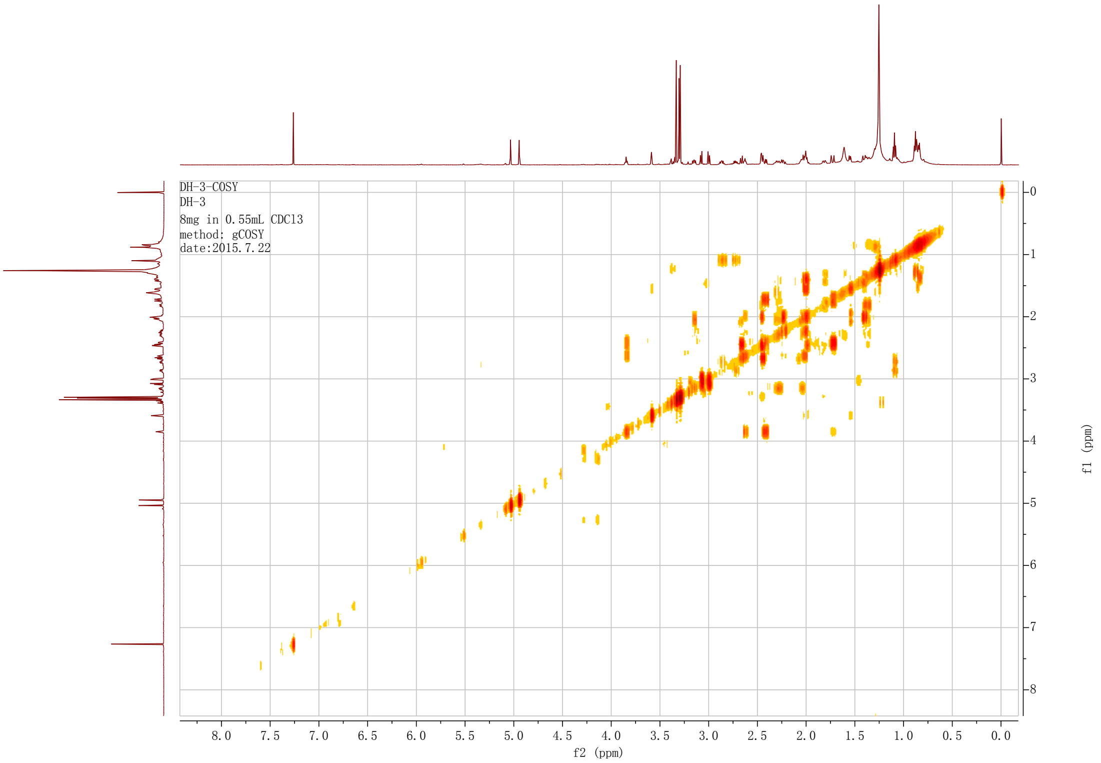

35 HMQC spectrum for compound **4**

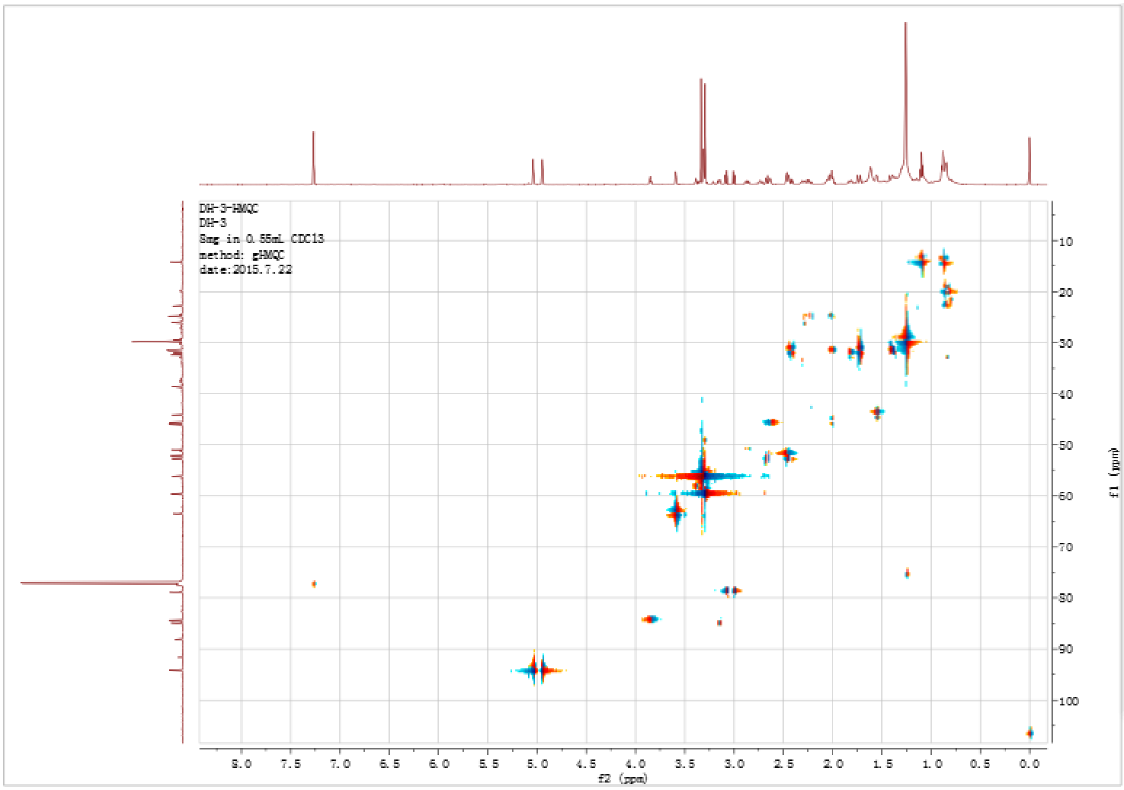

36 HMBC spectrum for compound **4**

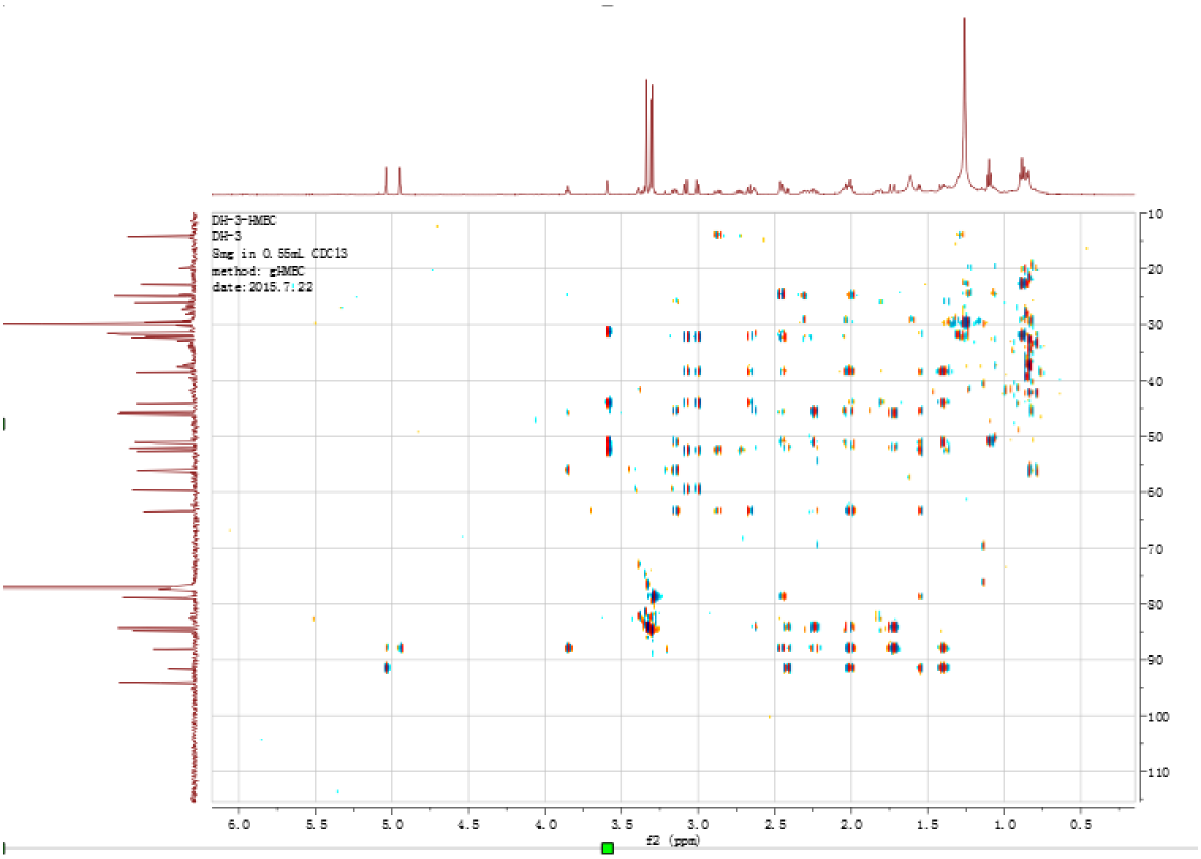

37 Noesy spectrum for compound **4**

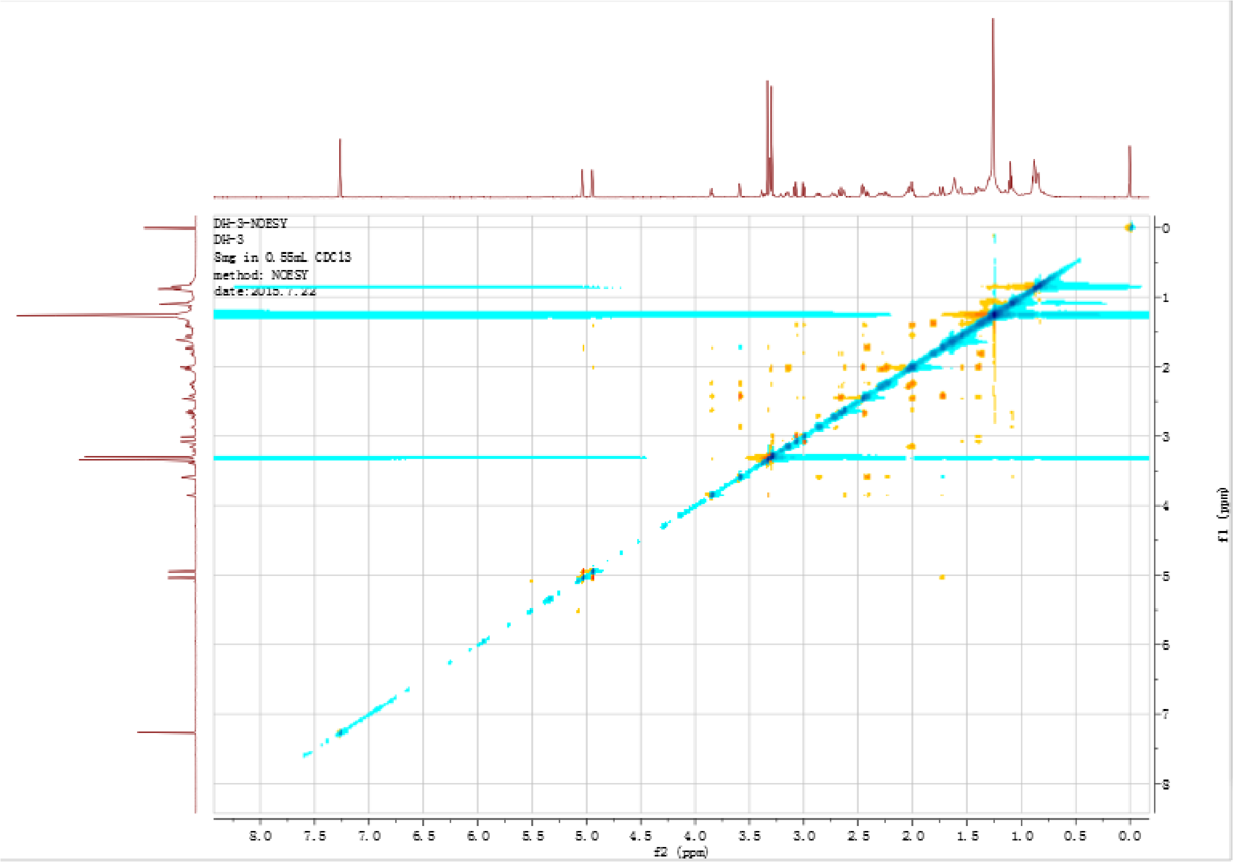

38 HR-ESI-MS spectrum for compound **5**

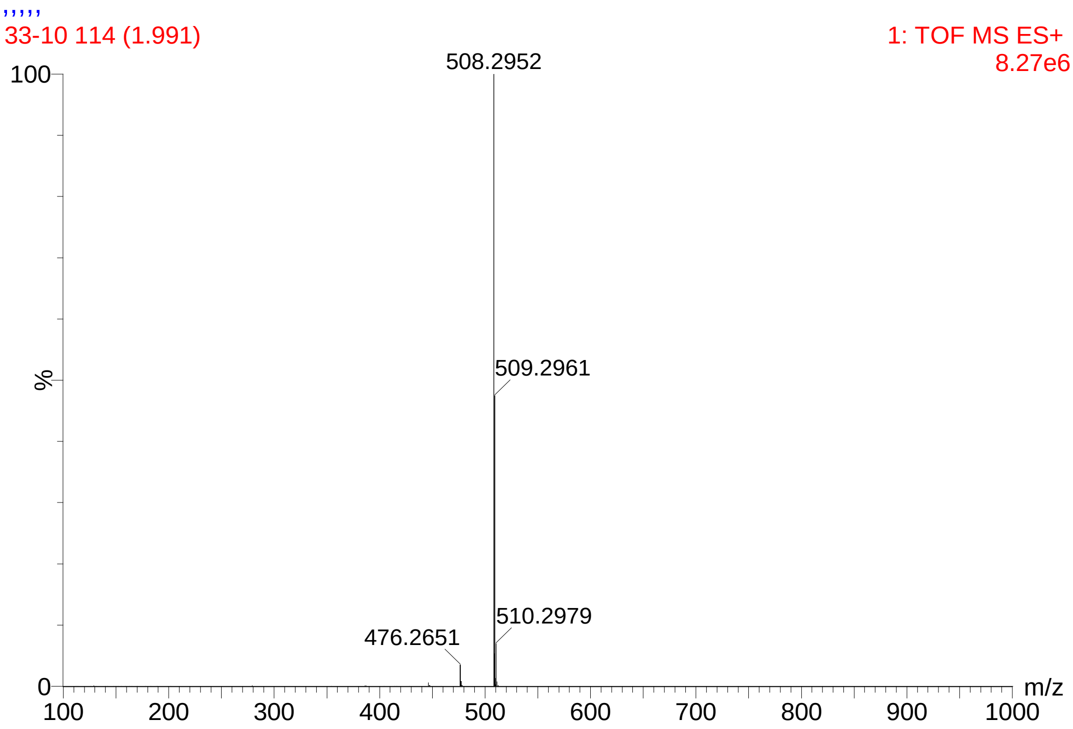

39 IR spectrum for compound **5**

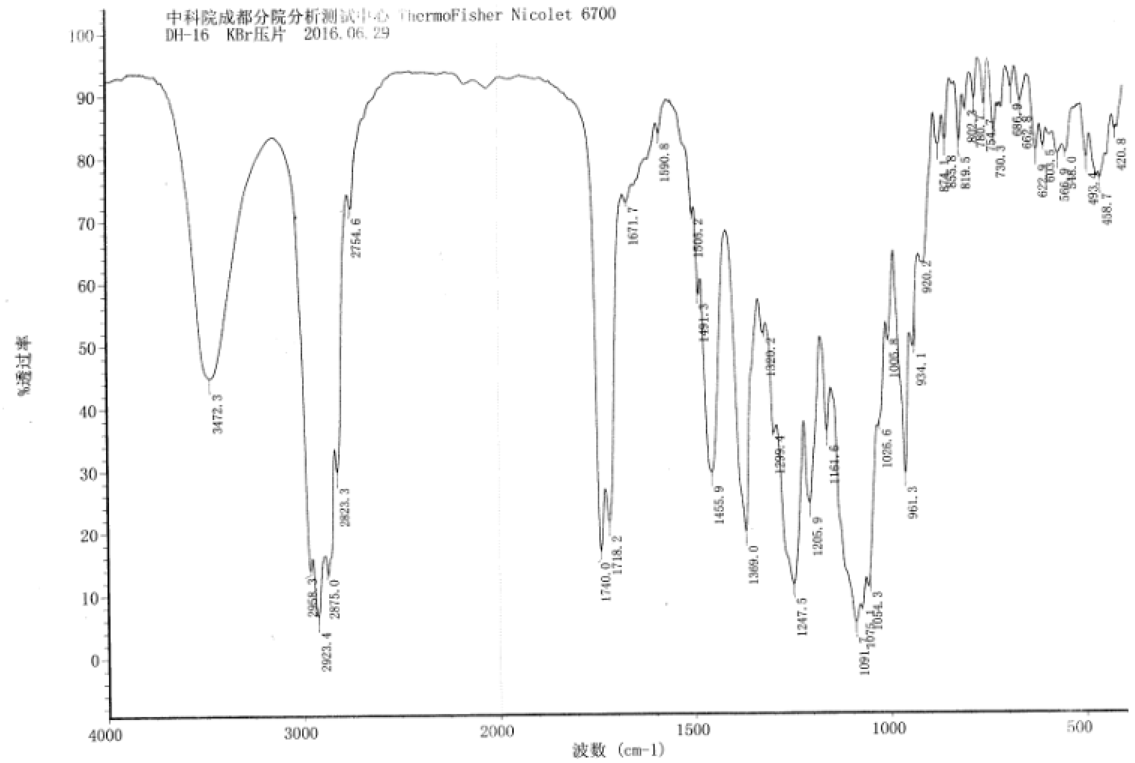

40 ^{1}H NMR spectrum for compound **5**

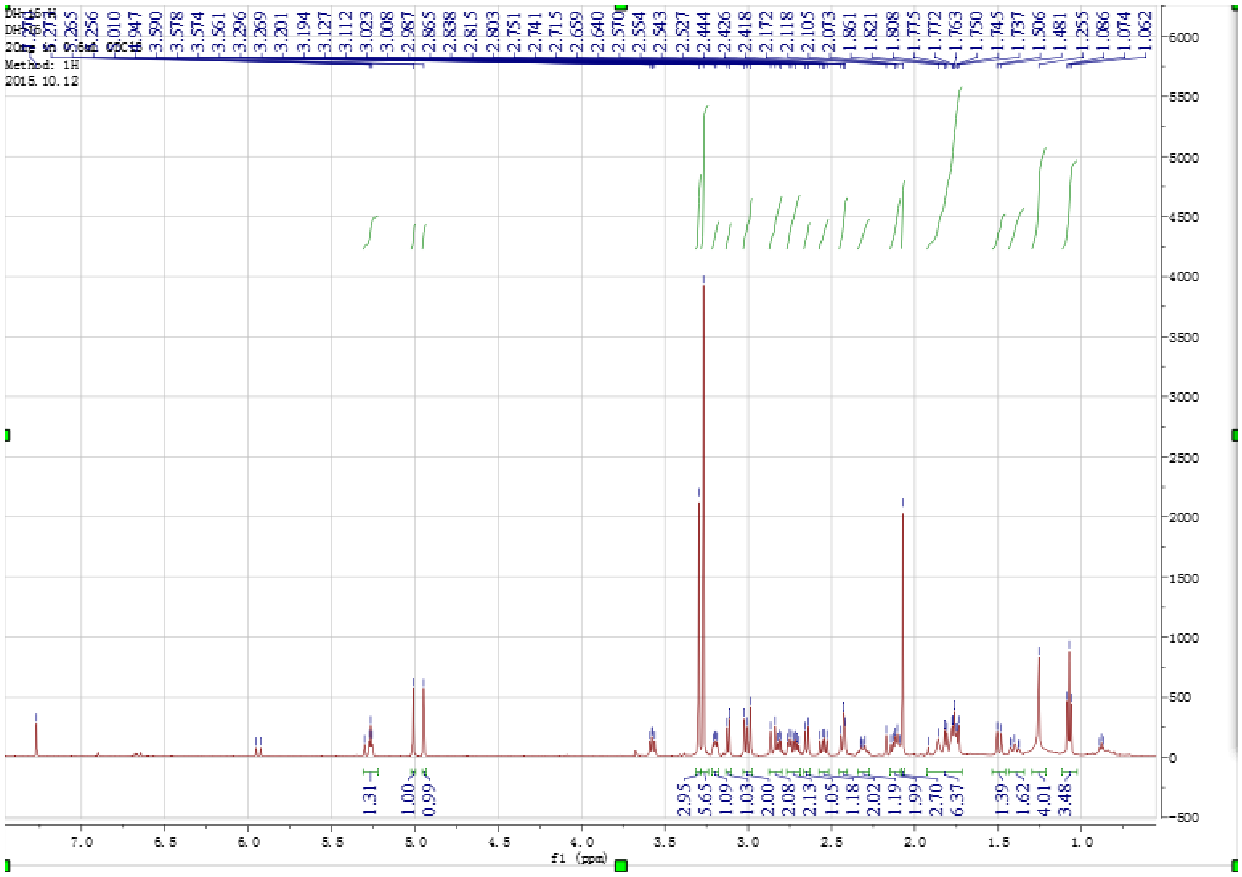

41 ^{13}C NMR spectrum for compound **5**

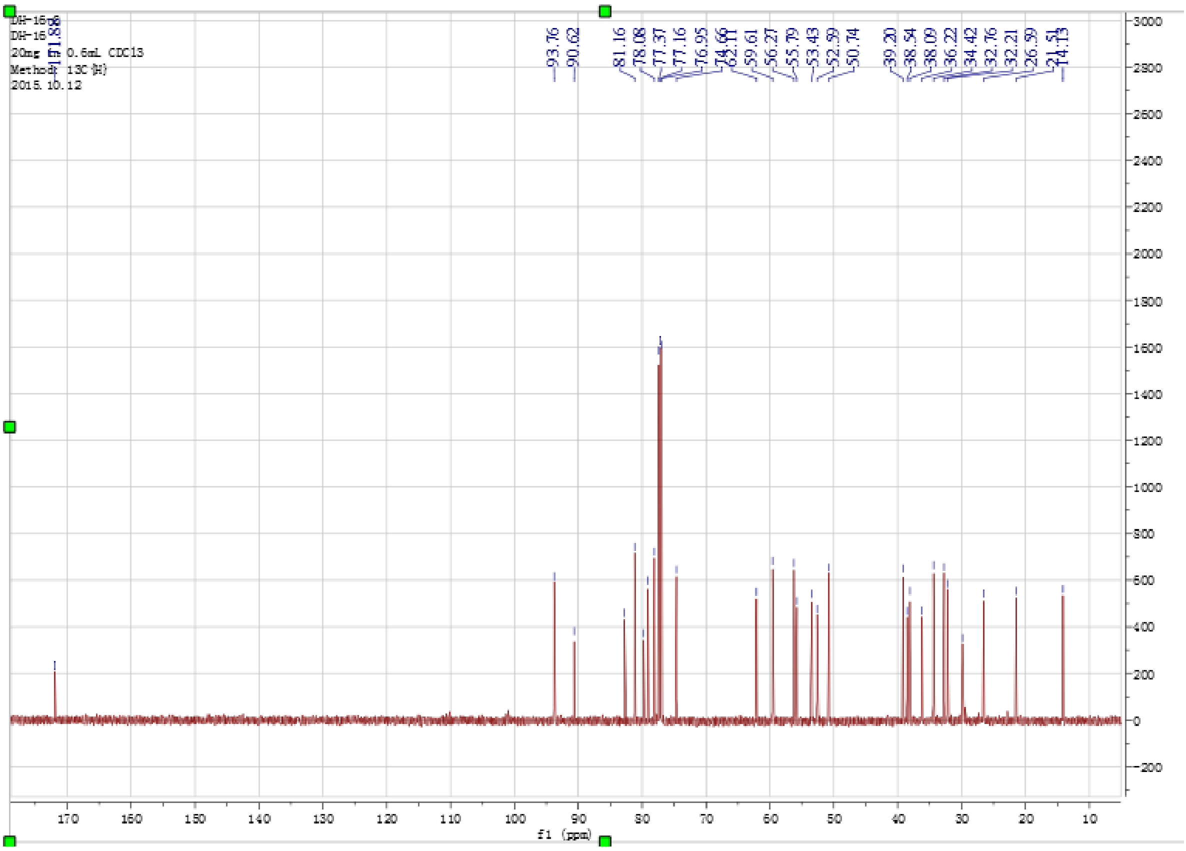

42 DEPT (θ = 135°) spectrum for compound **5**

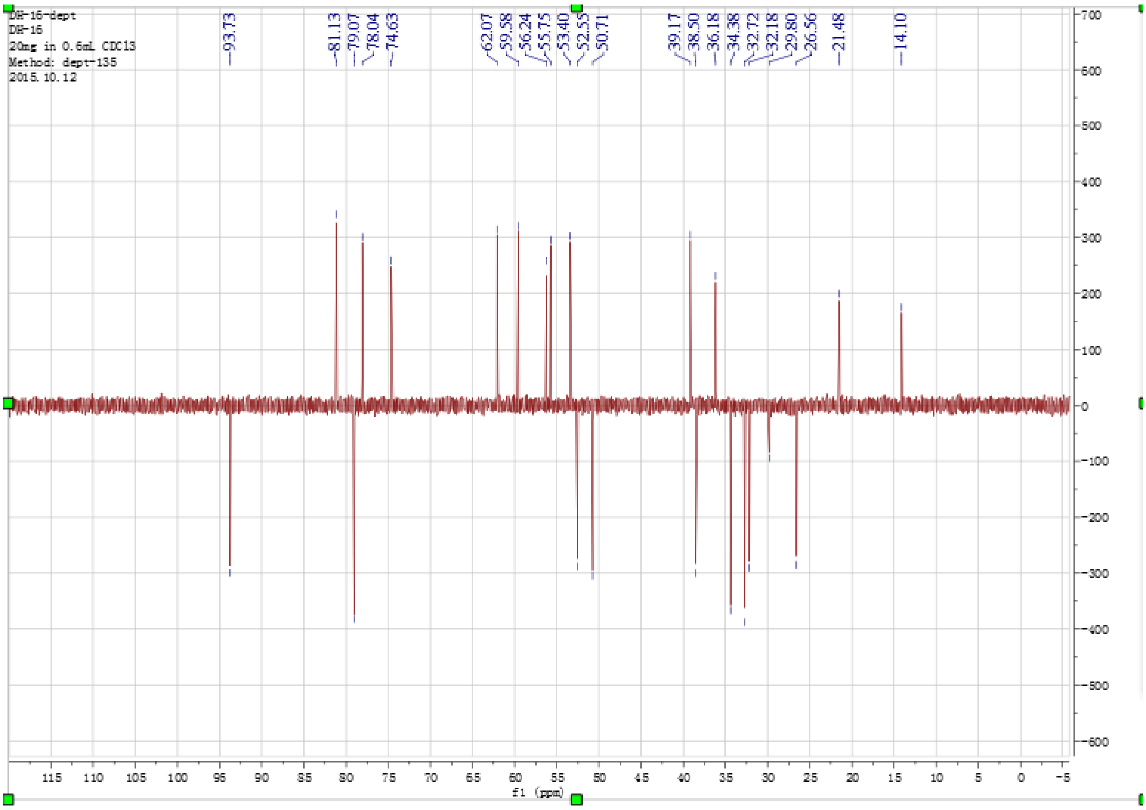

43 ^1H-^1H COSY spectrum for compound **5**

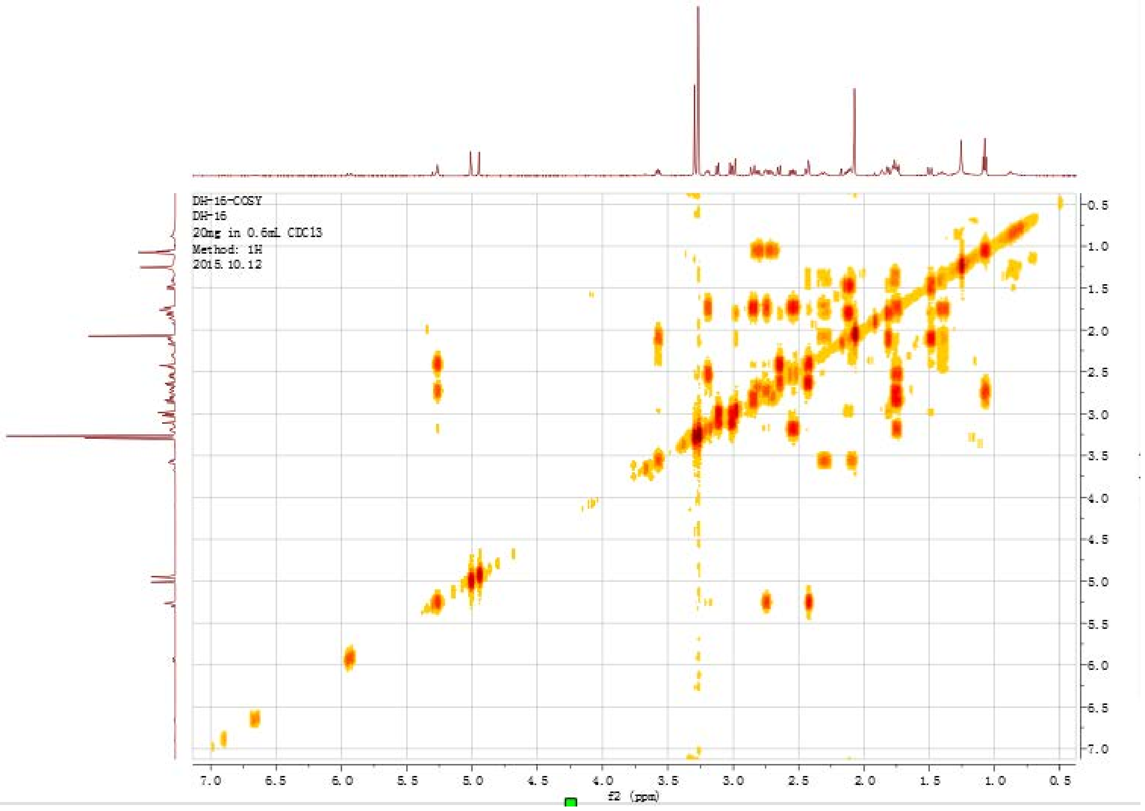

44 HMQC spectrum for compound **5**

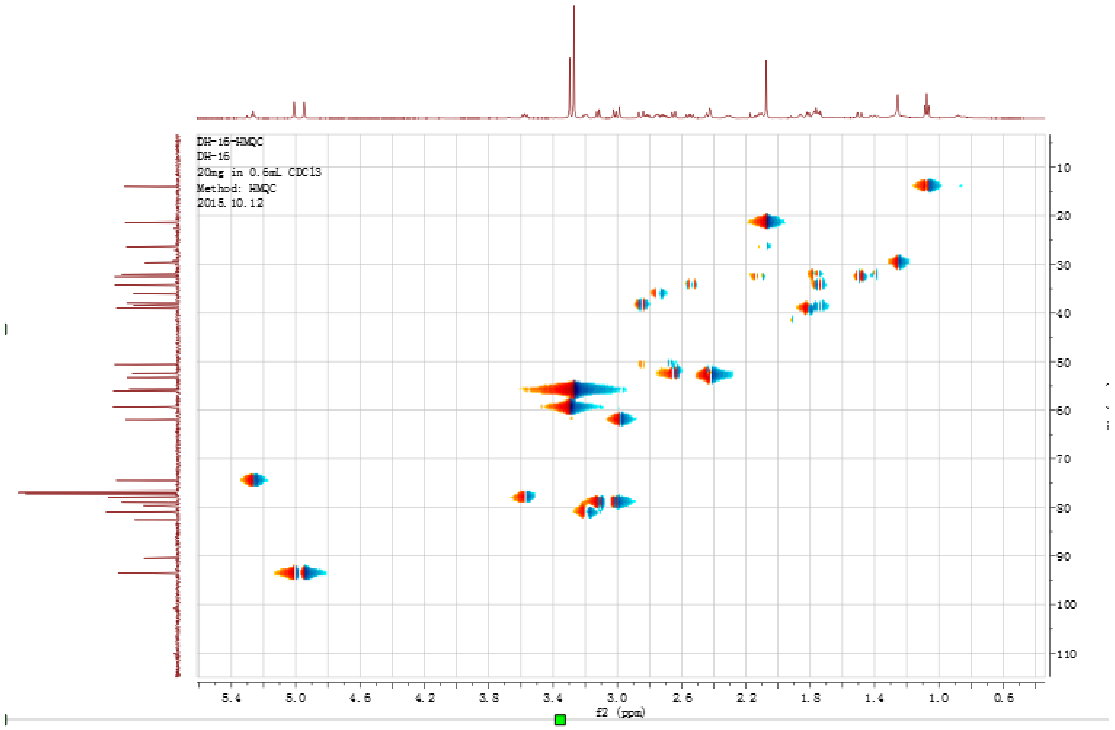

45 HMBC spectrum for compound **5**

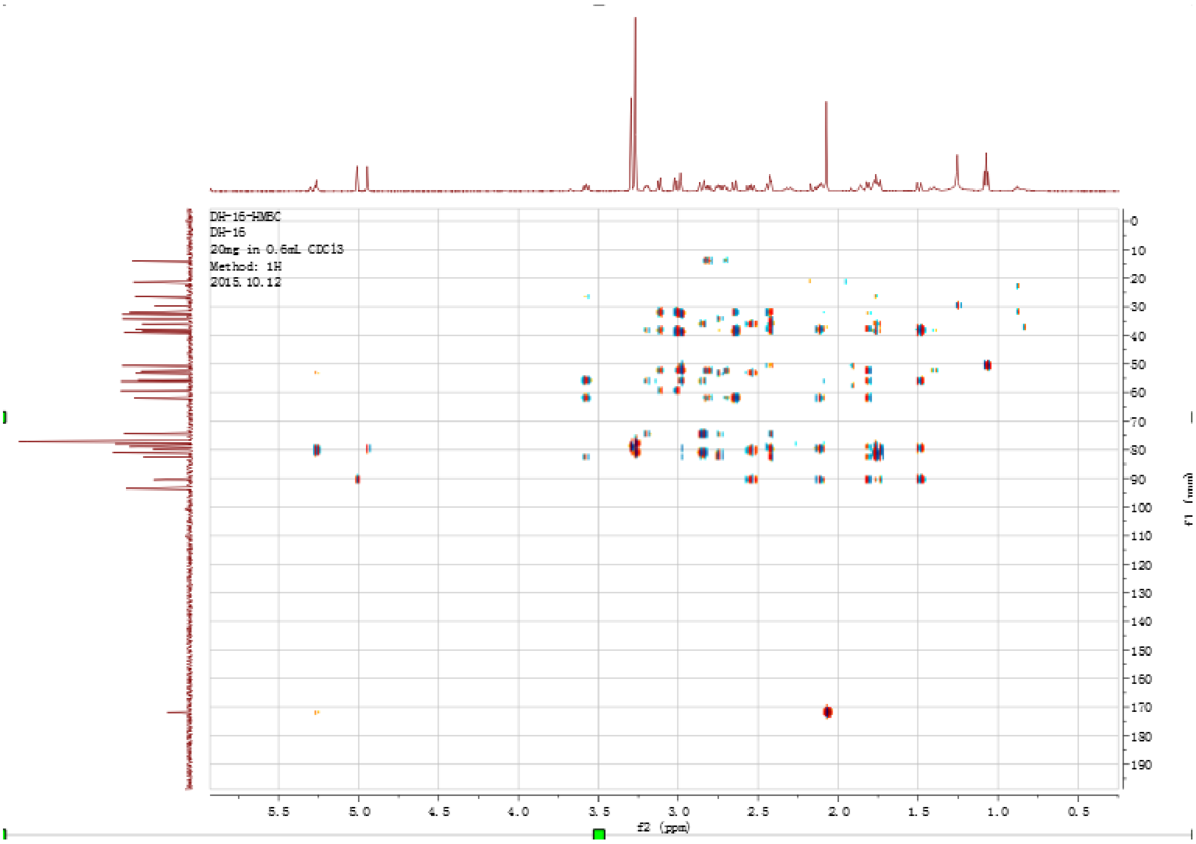

46 Noesy spectrum for compound **5**

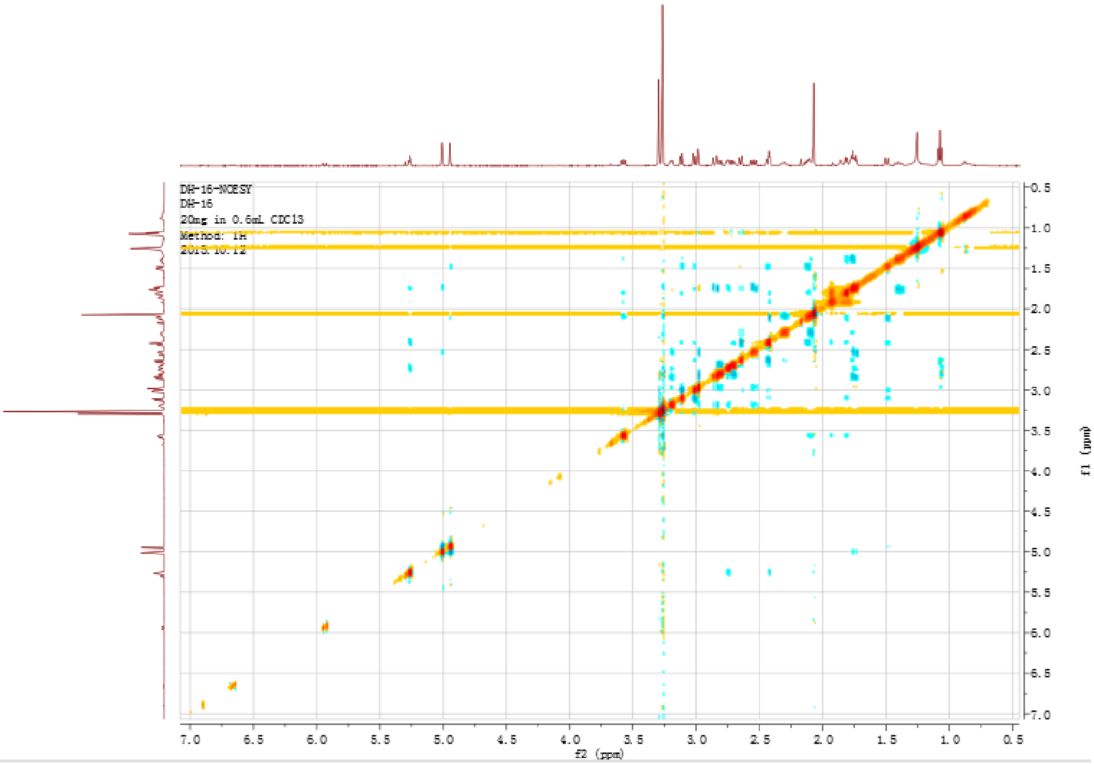

47 HR-ESI-MS spectrum for compound **6**

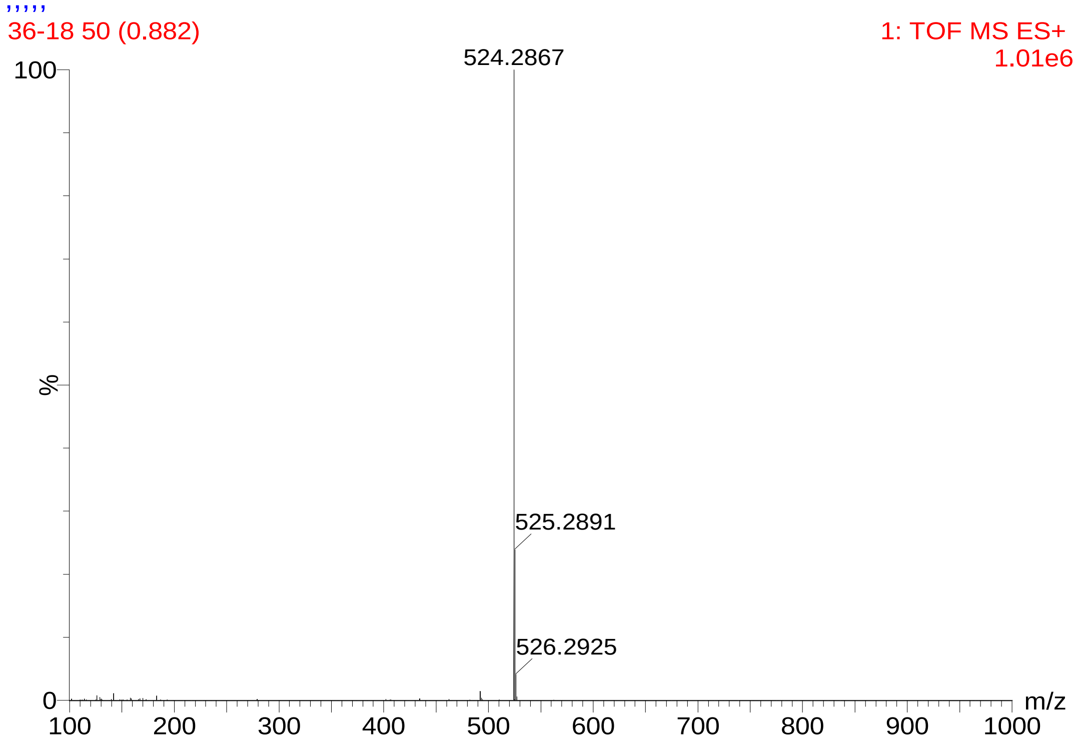

48 IR spectrum for compound **6**

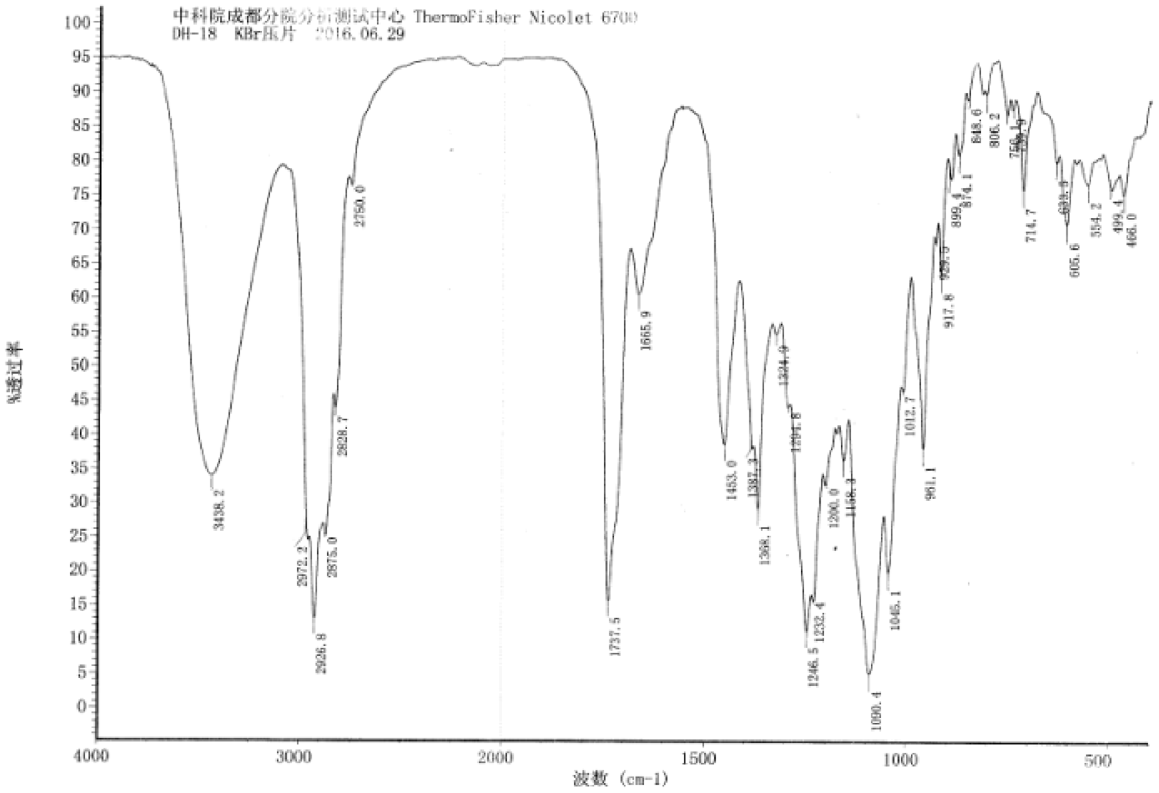

49 ^{1}H NMR spectrum for compound **6**

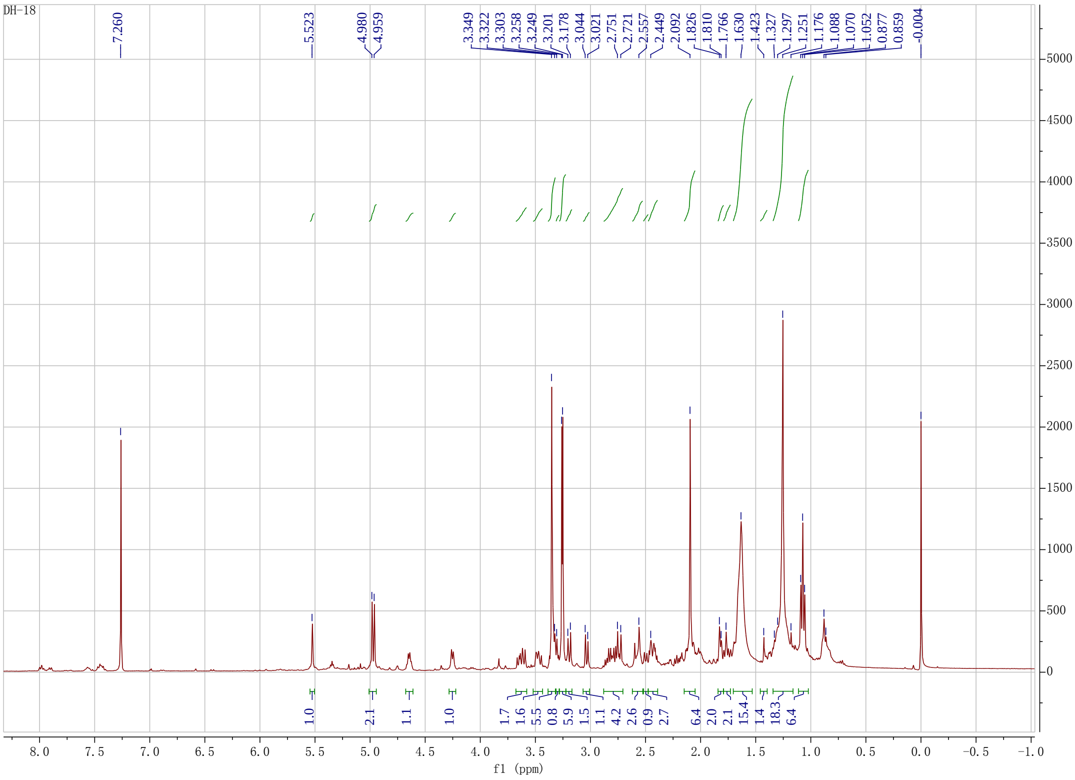

50 ^{13}C NMR spectrum for compound **6**

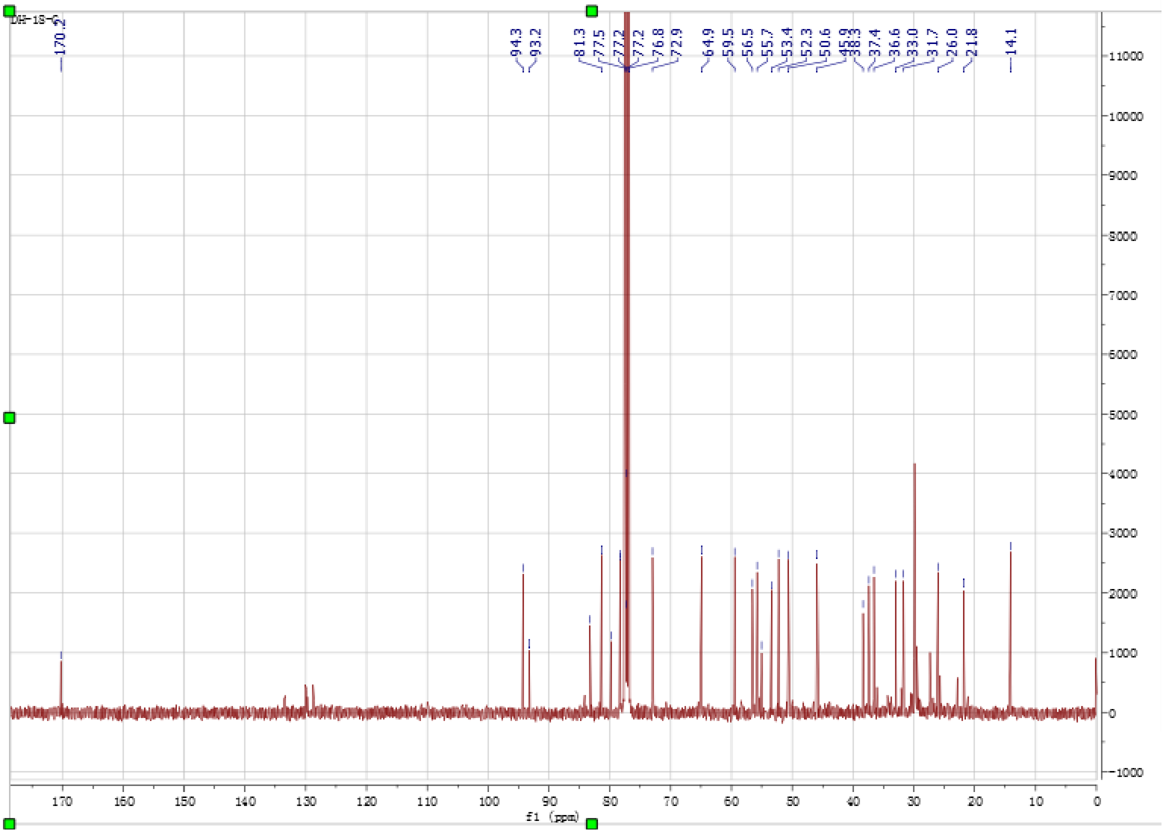

51 DEPT ($\theta = 135^{\circ}$) spectrum for compound **6**

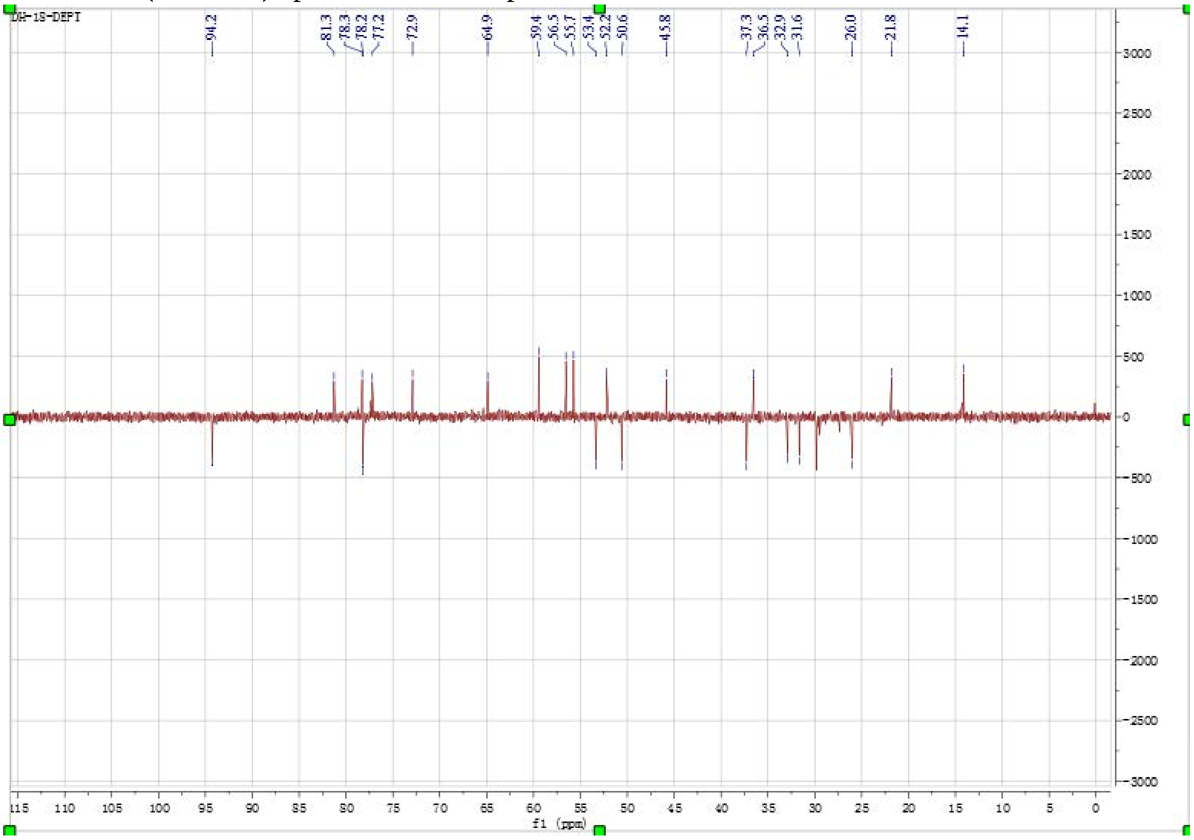

52 ^{1}H-^{1}H COSY spectrum for compound **6**

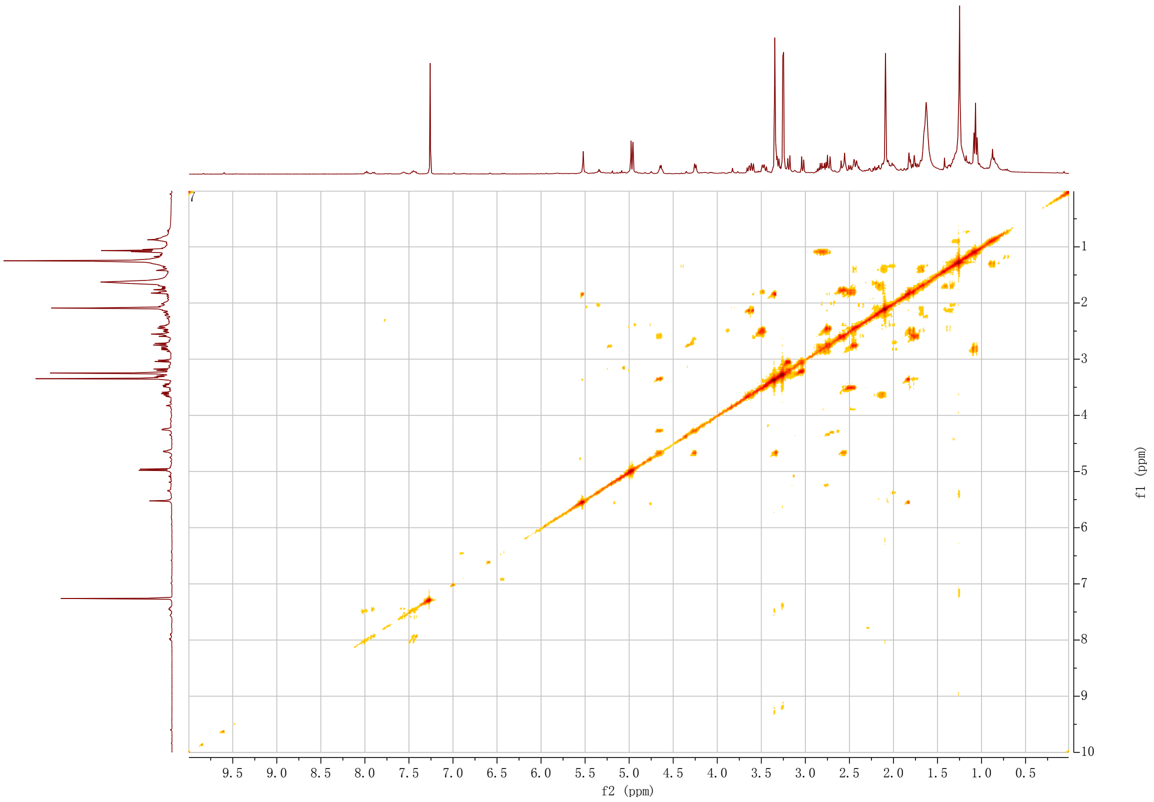

53 HMQC spectrum for compound **6**

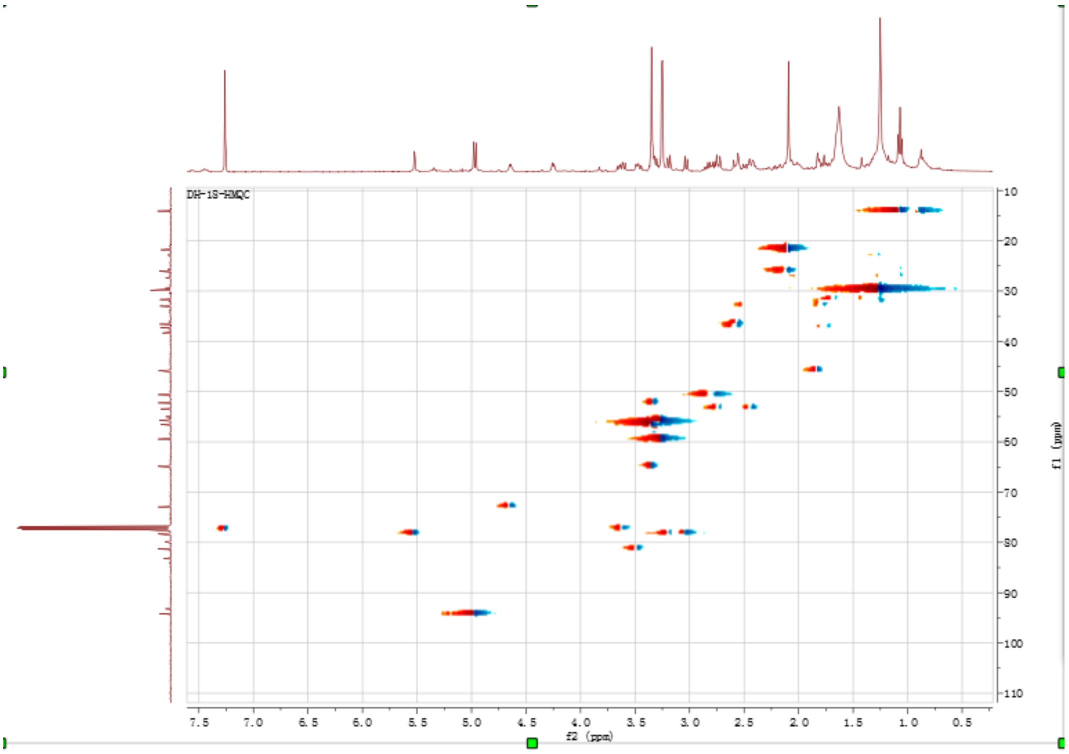

54 HMBC spectrum for compound **6**

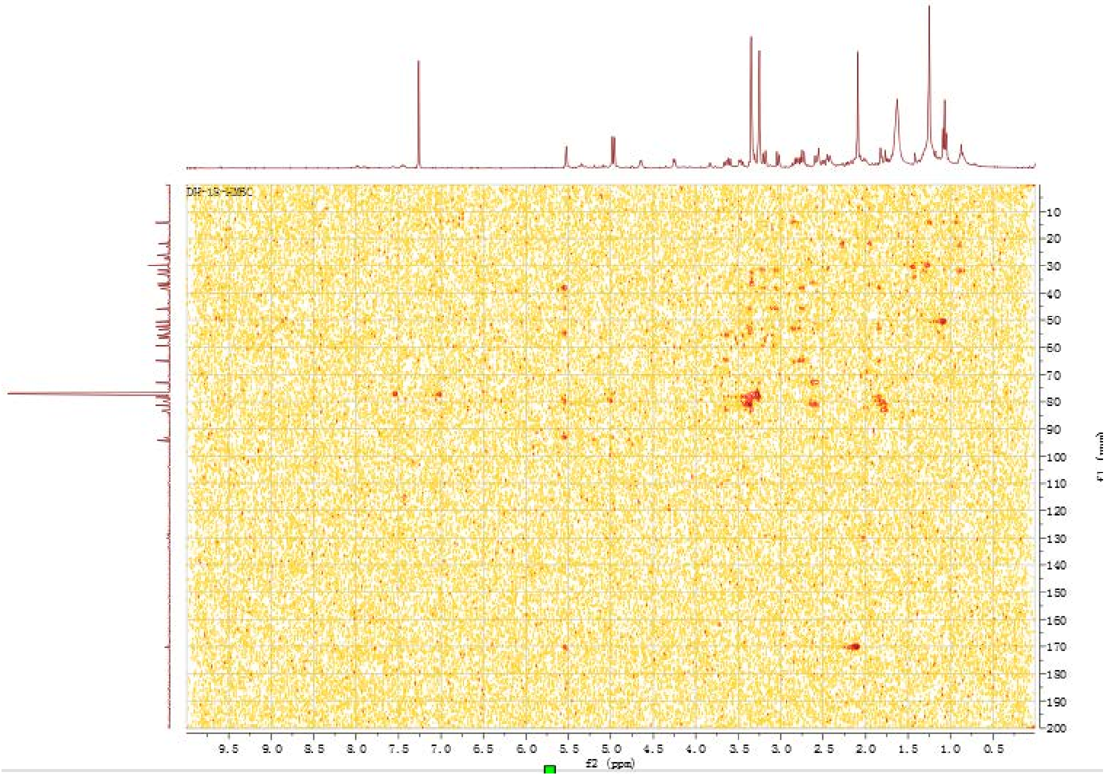

55 HR-ESI-MS spectrum for compound **7**

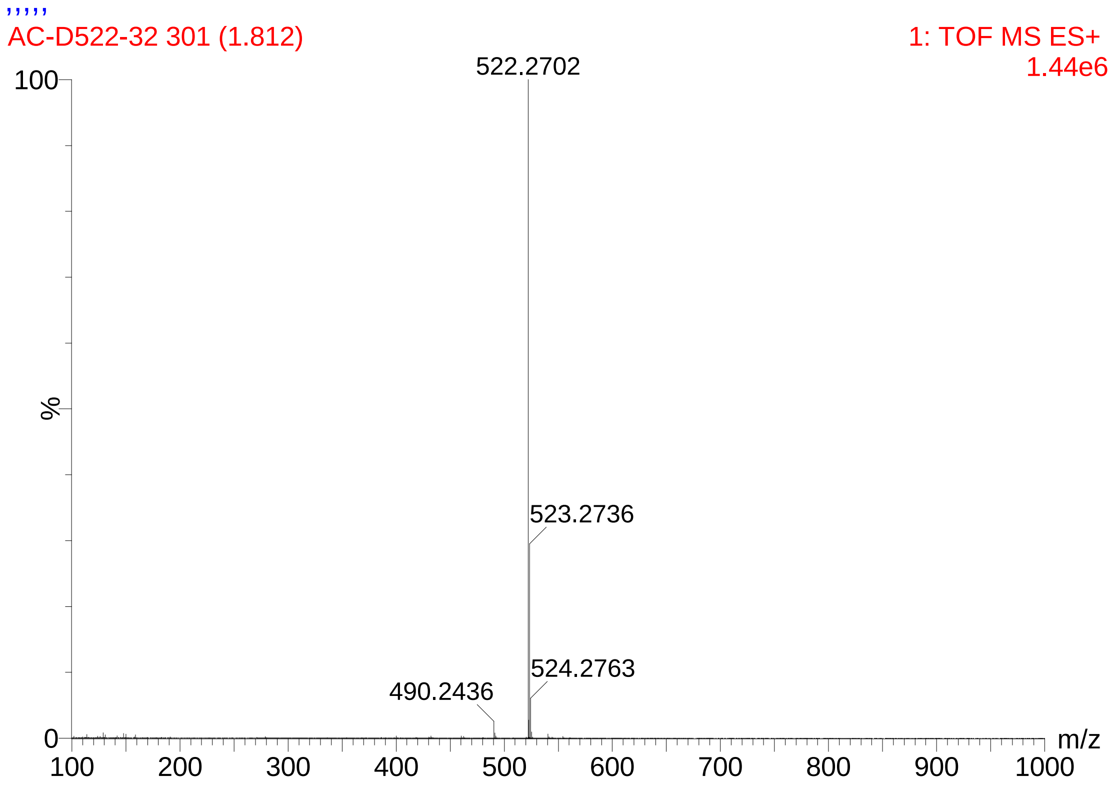

56 IR spectrum for compound **7**

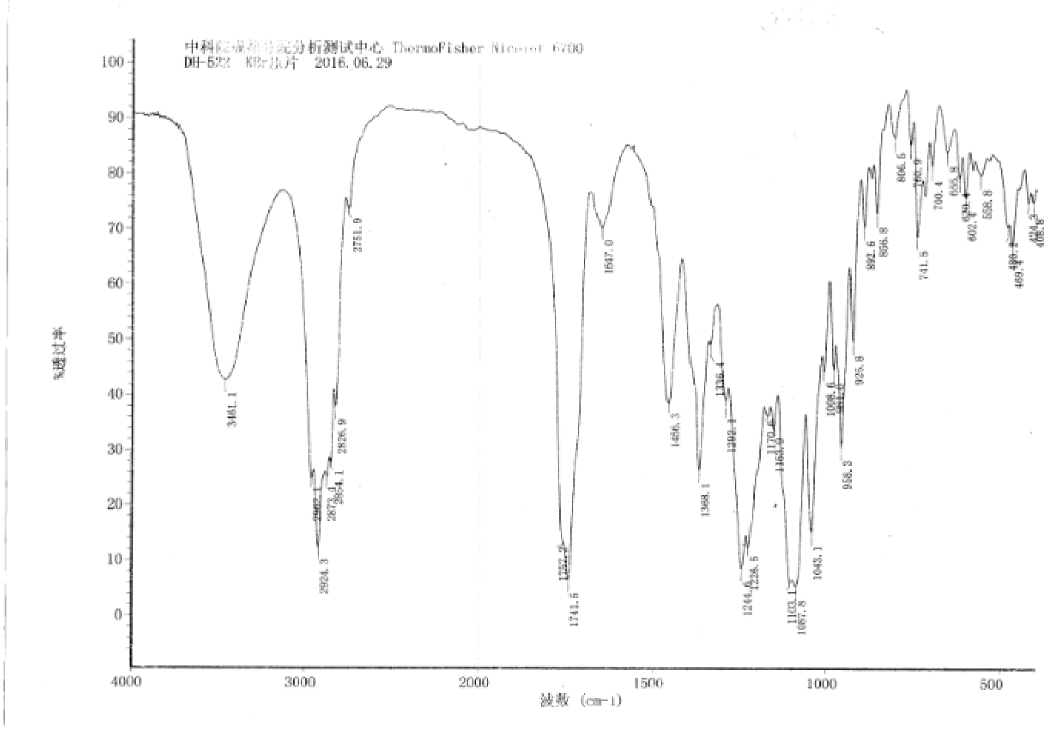

57 ^{1}H NMR spectrum for compound **7**

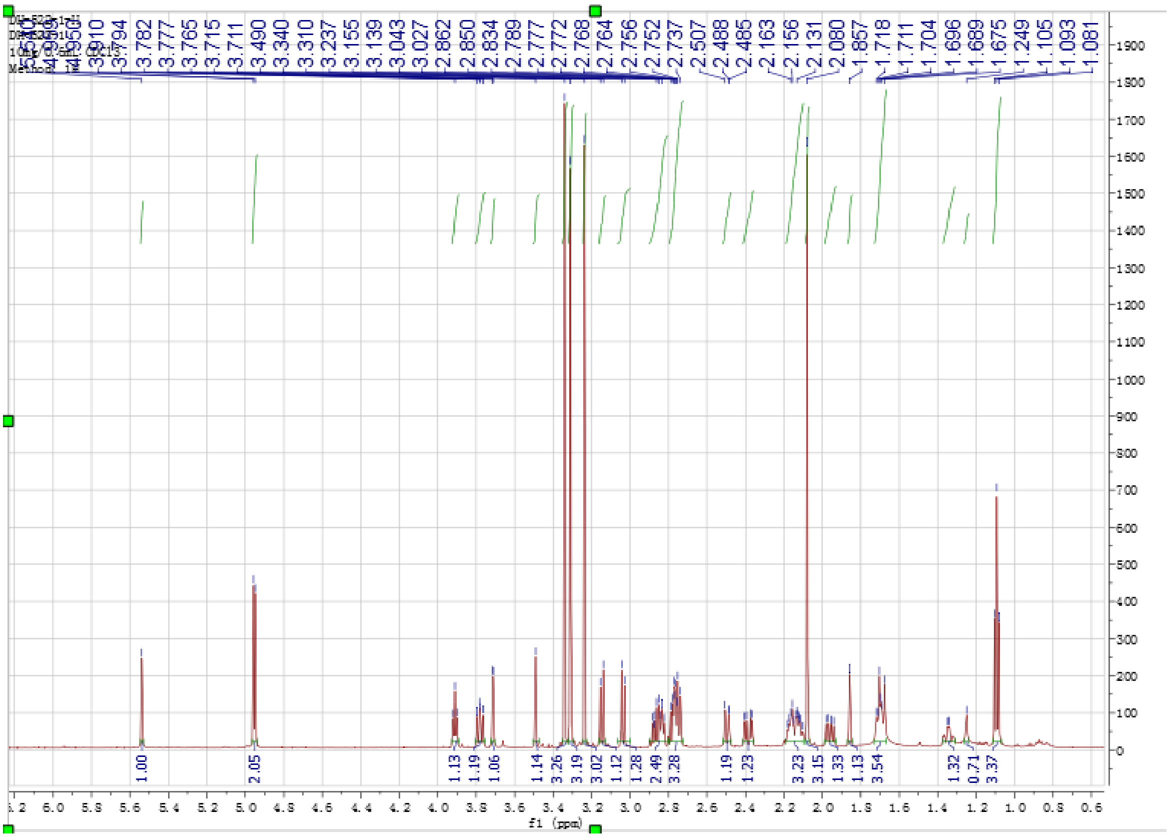

58 ^{13}C NMR spectrum for compound **7**

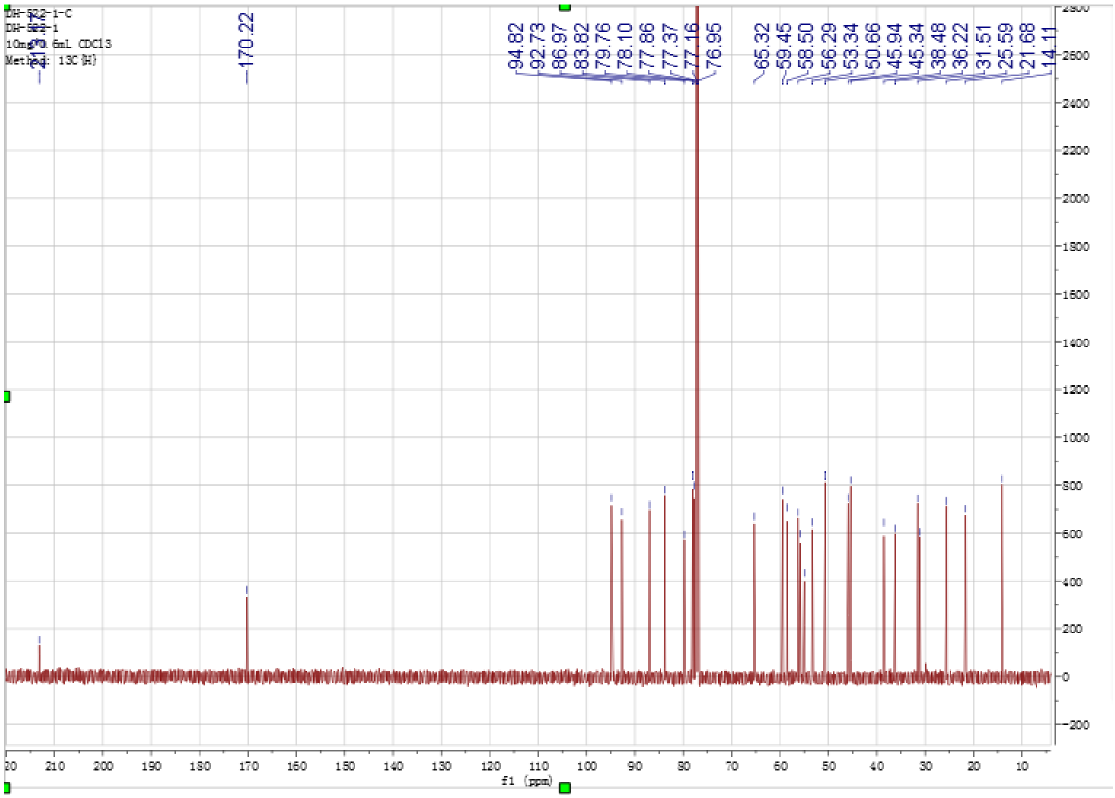

59 DEPT ($\theta = 135°$) spectrum for compound **7**

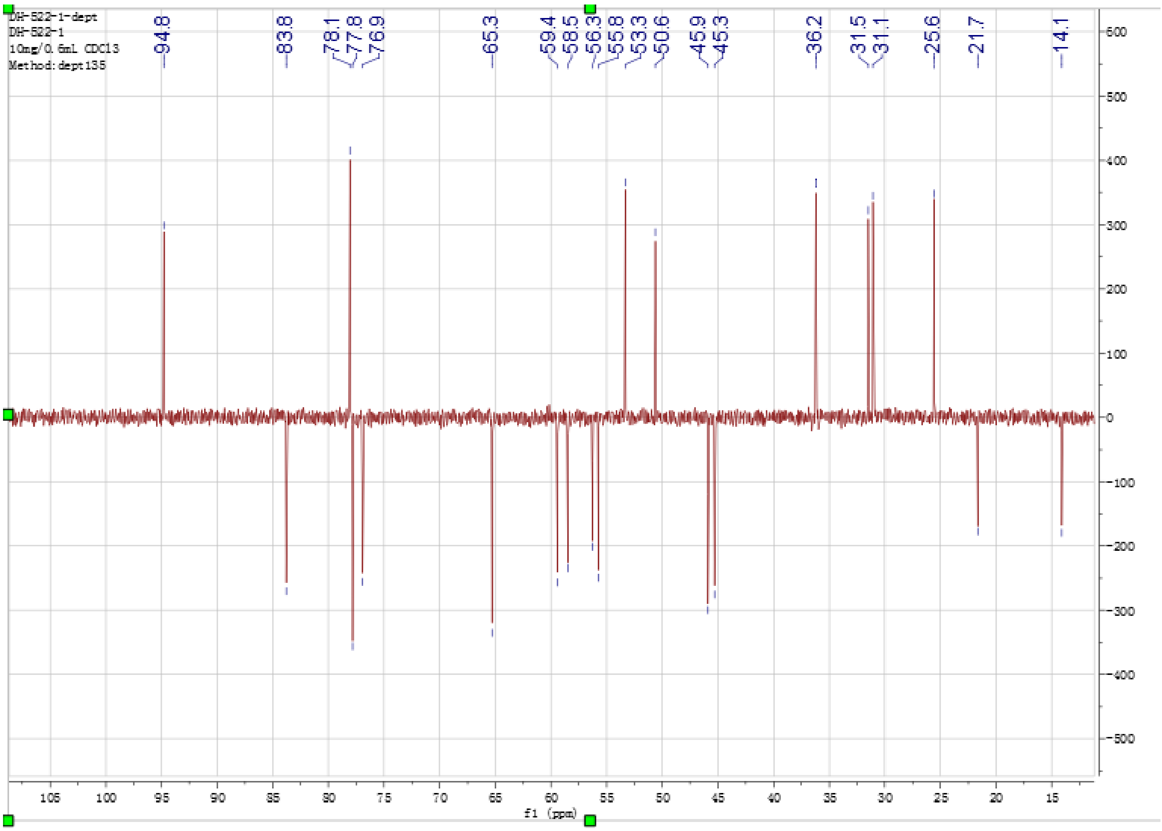

60 ^{1}H-^{1}H COSY spectrum for compound **7**

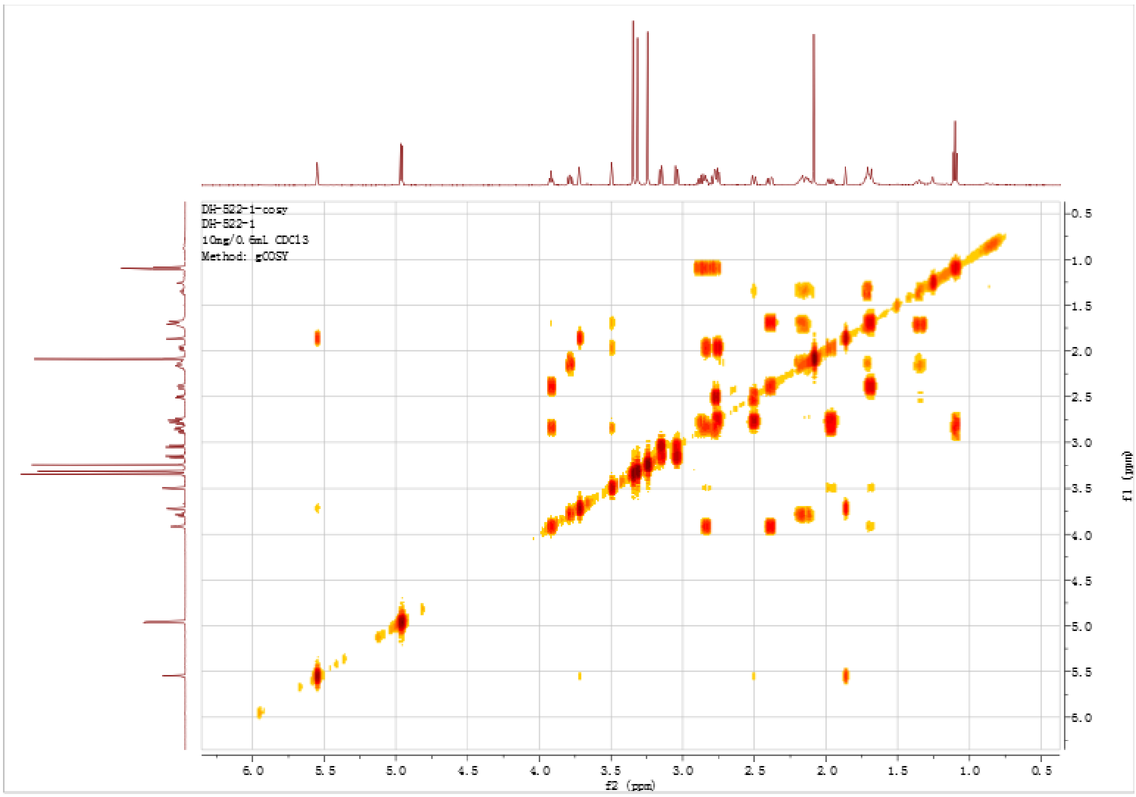

61 HMQC spectrum for compound **7**

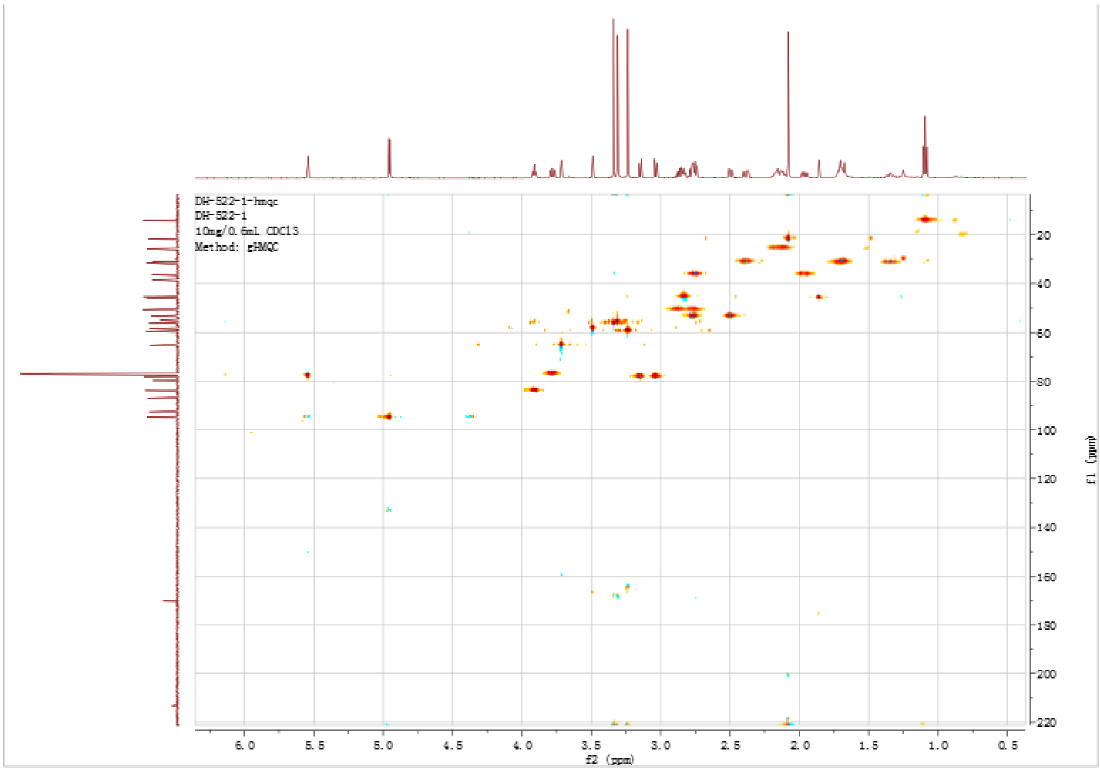

62 HMBC spectrum for compound **7**

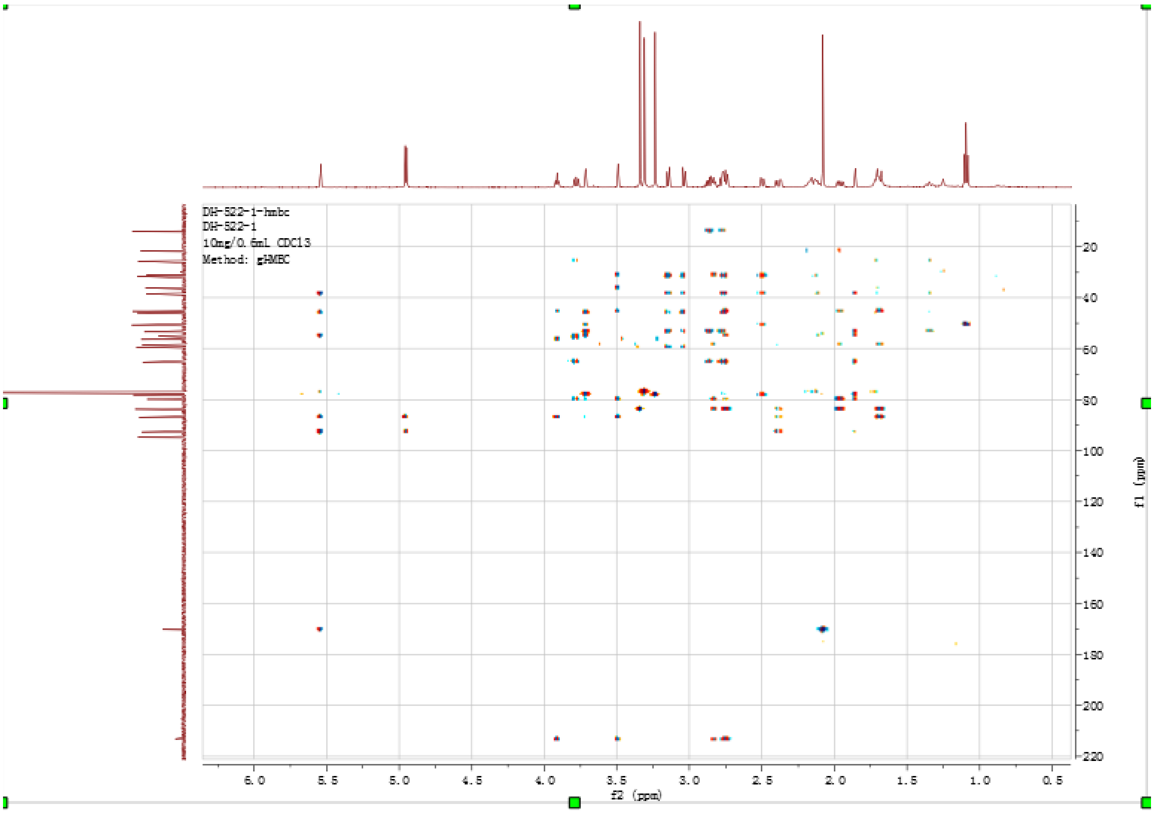

63 Noesy spectrum for compound **7**

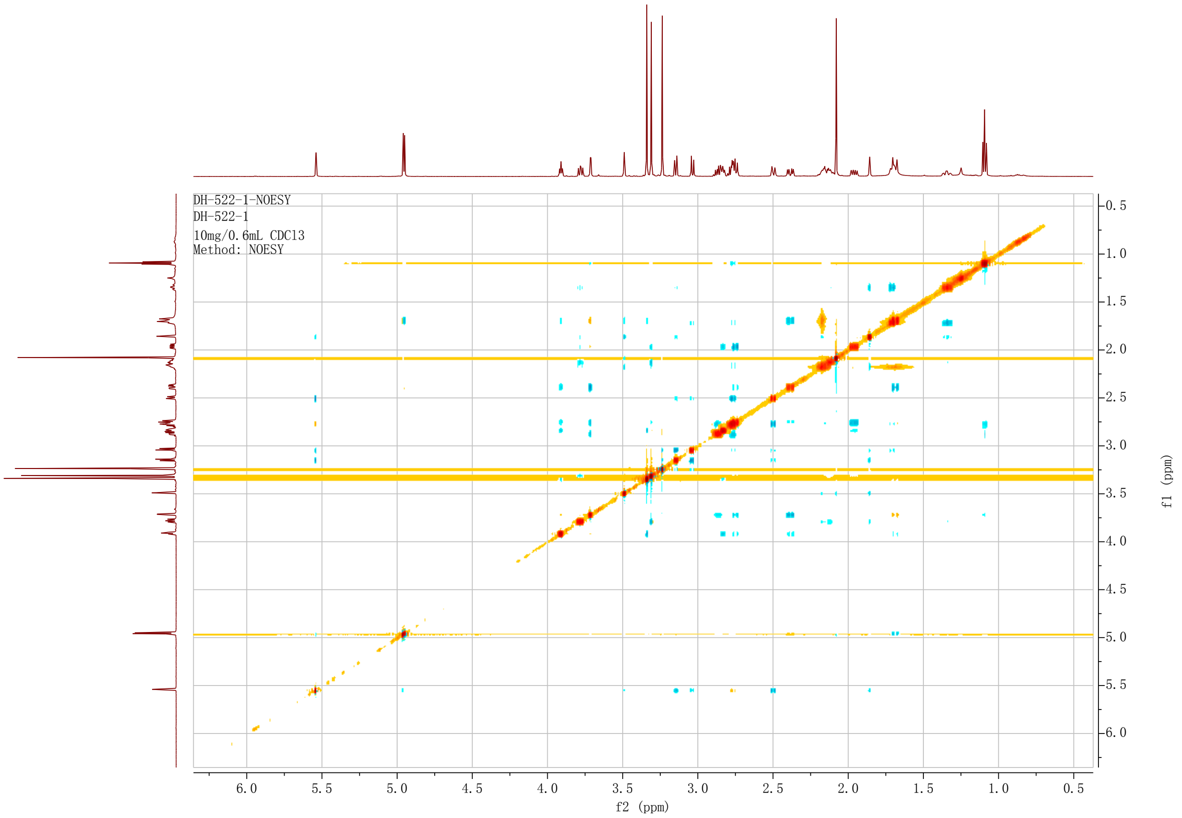

64 HR-ESI-MS spectrum for compound **8**

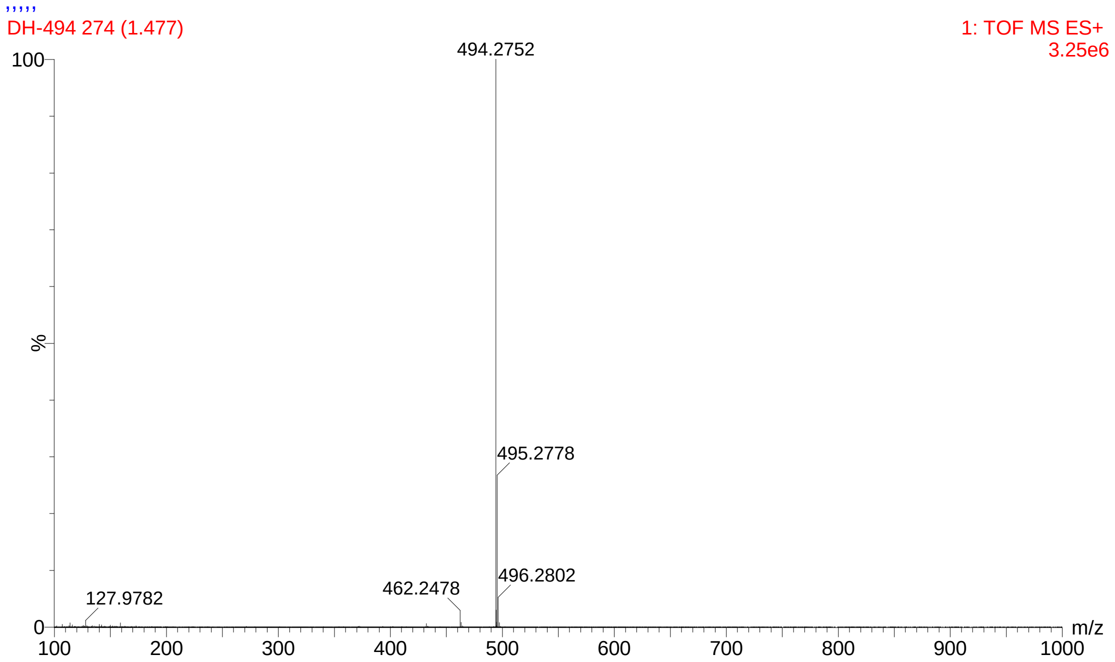

65 IR spectrum for compound **8**

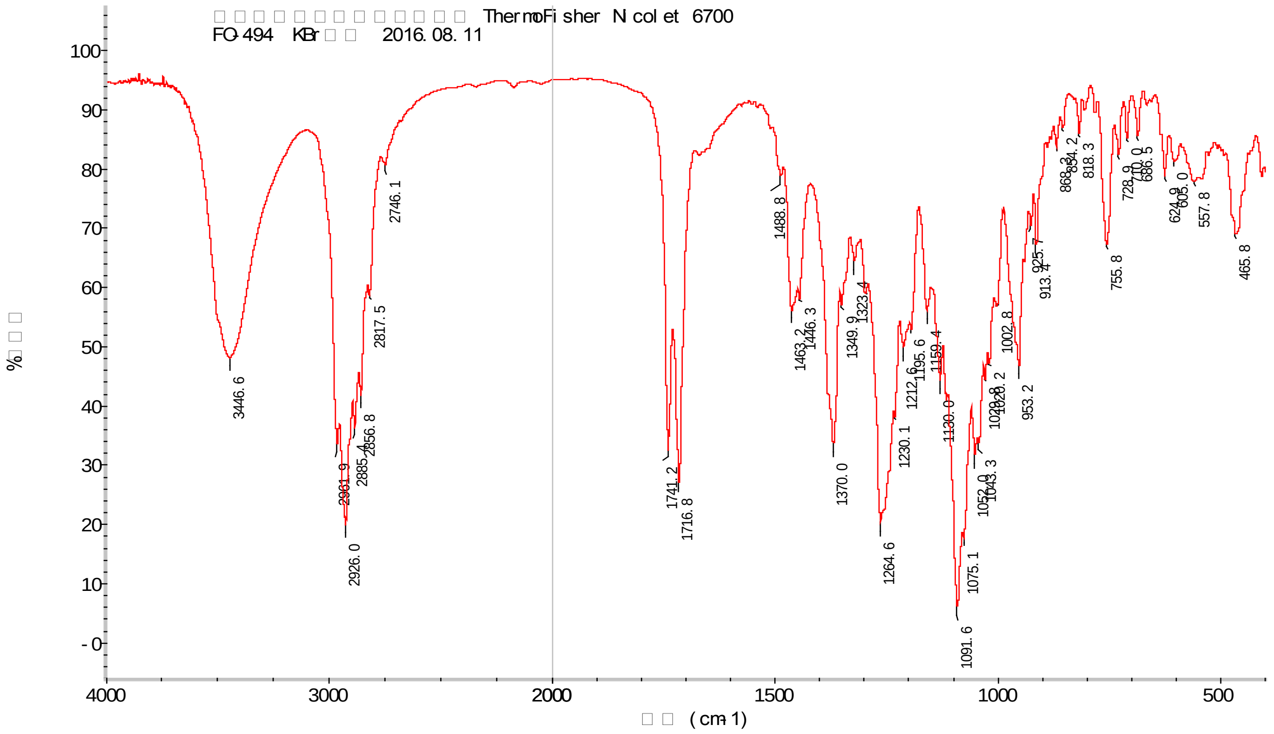

66 ^{1}H NMR spectrum for compound **8**

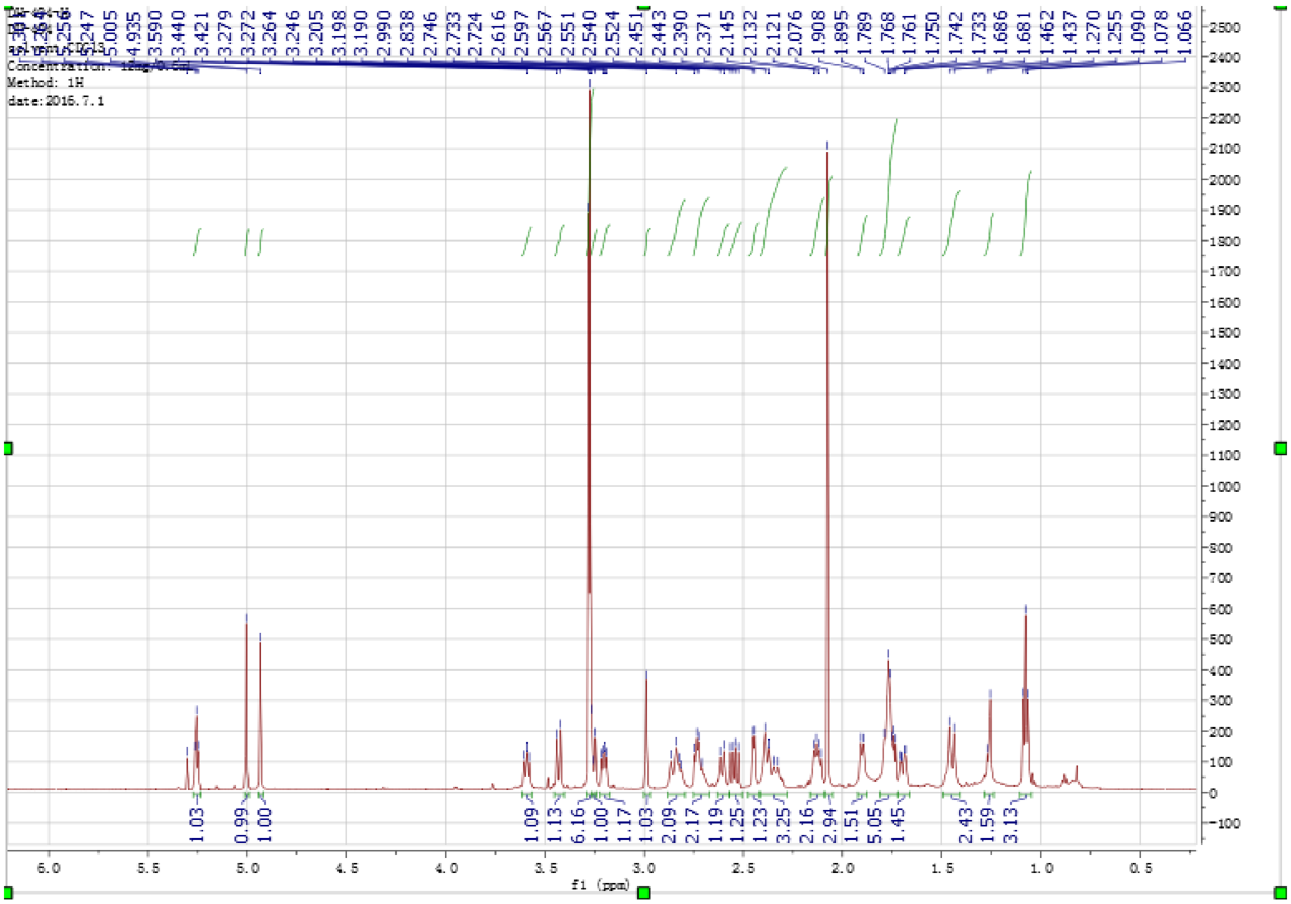

67 ^{13}C NMR spectrum for compound **8**

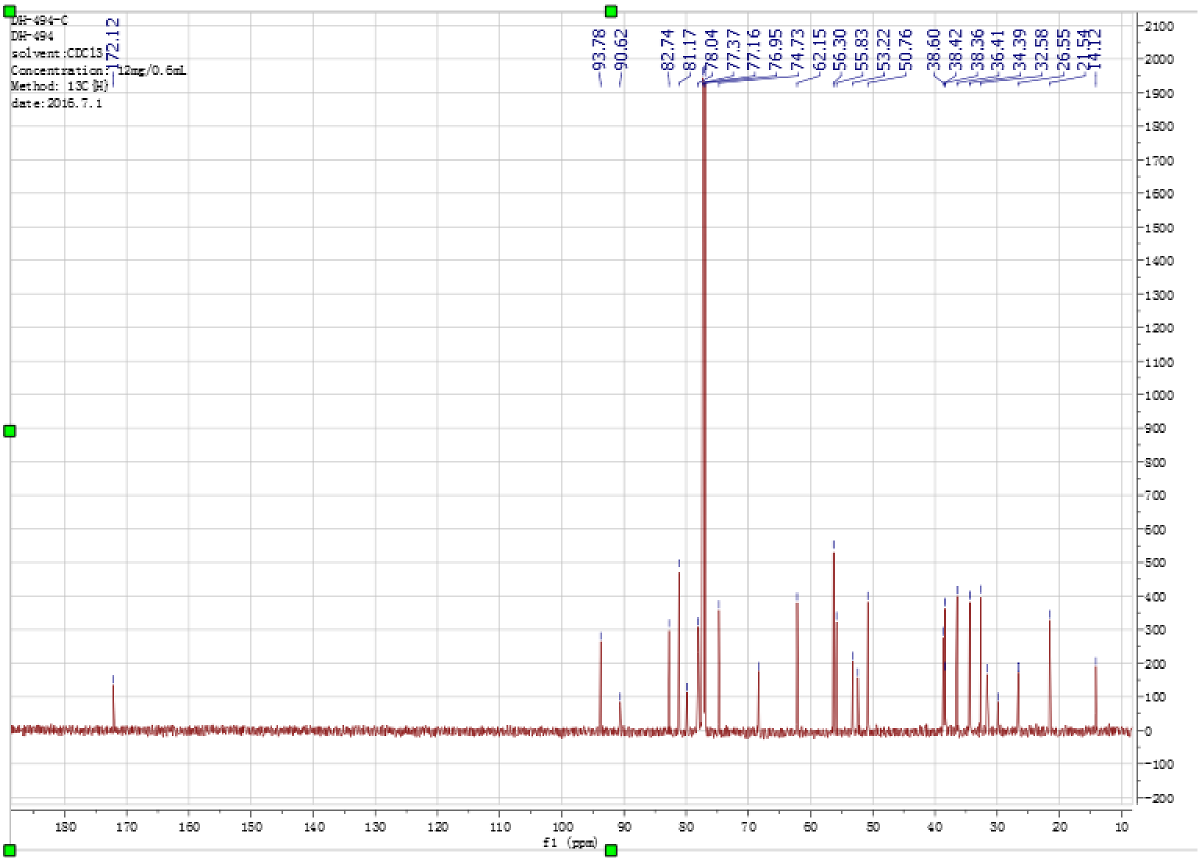

68 DEPT ($\theta = 135^\circ$) spectrum for compound **8**

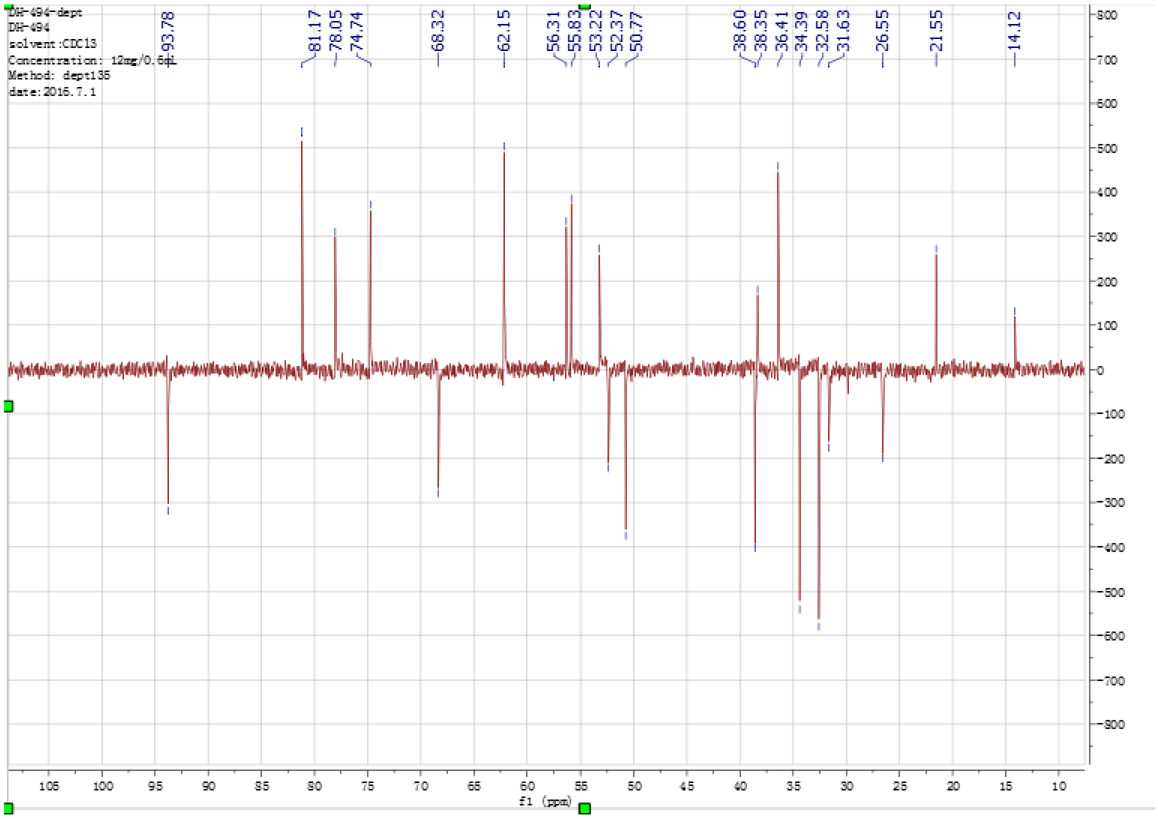

69 ^{1}H-^{1}H COSY spectrum for compound **8**

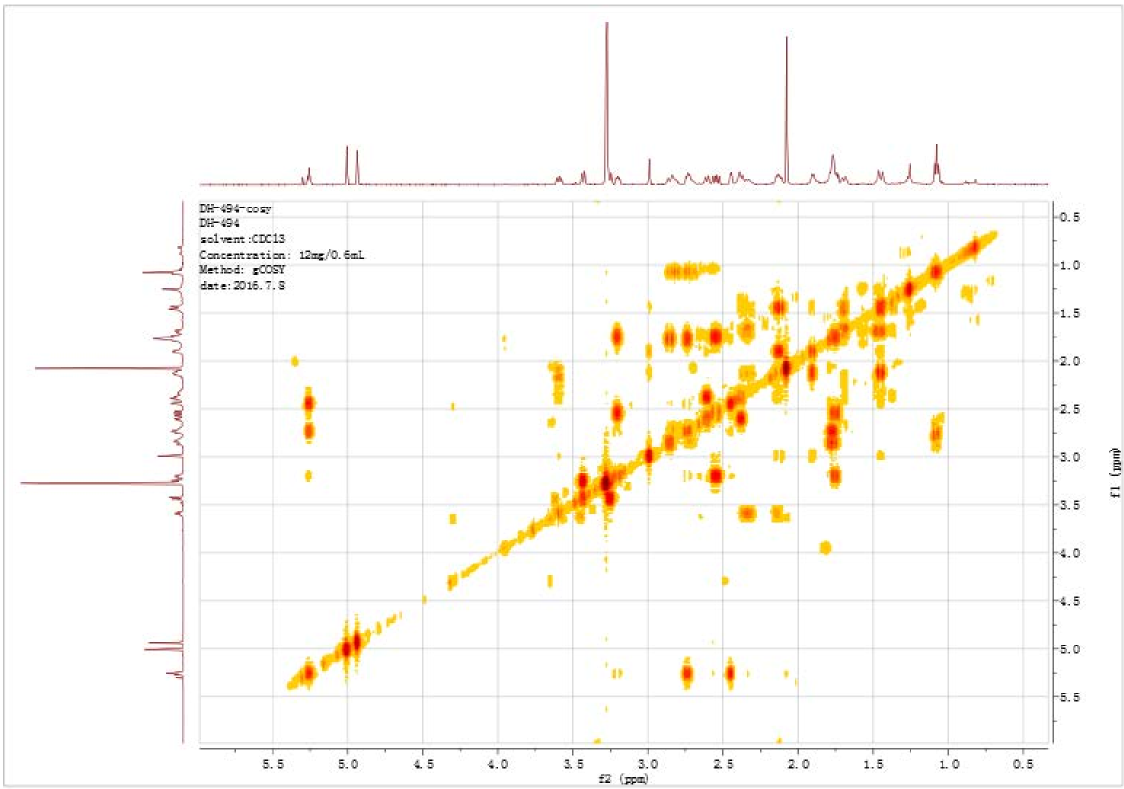

70 HMQC spectrum for compound **8**

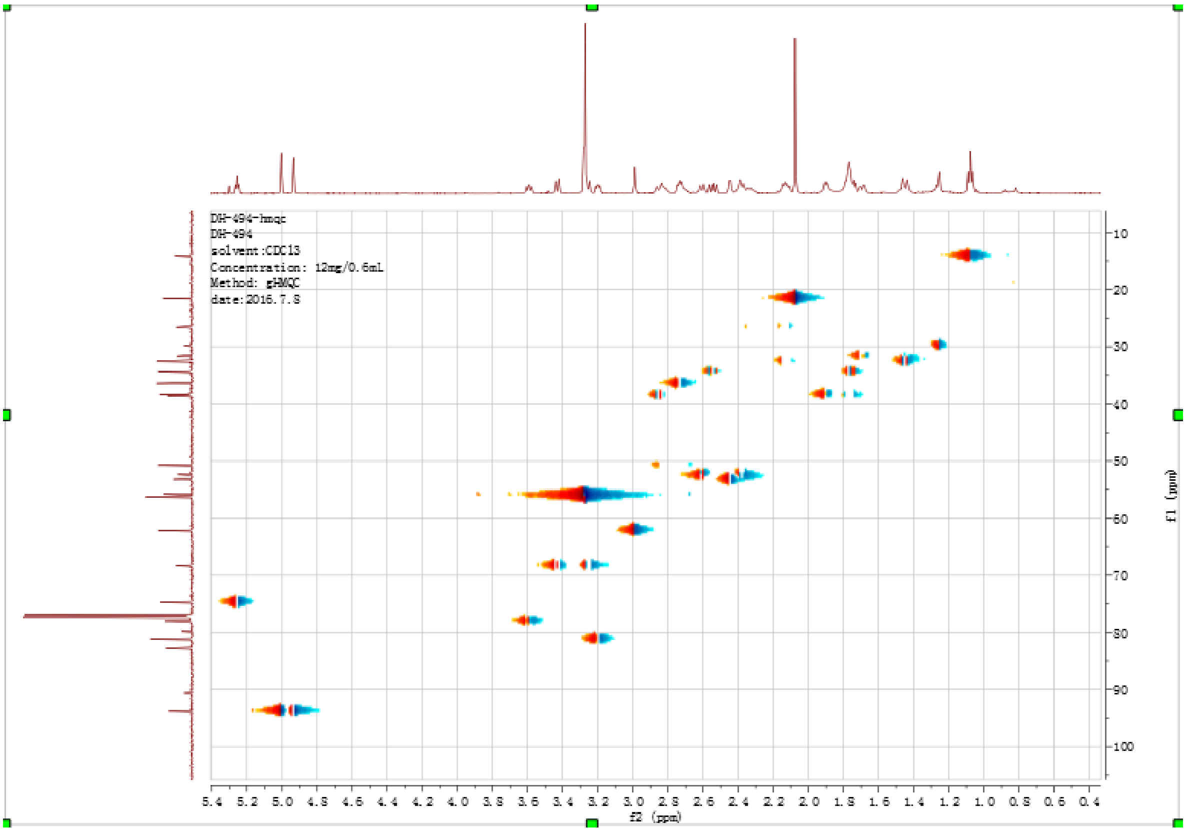

71 HMBC spectrum for compound **8**

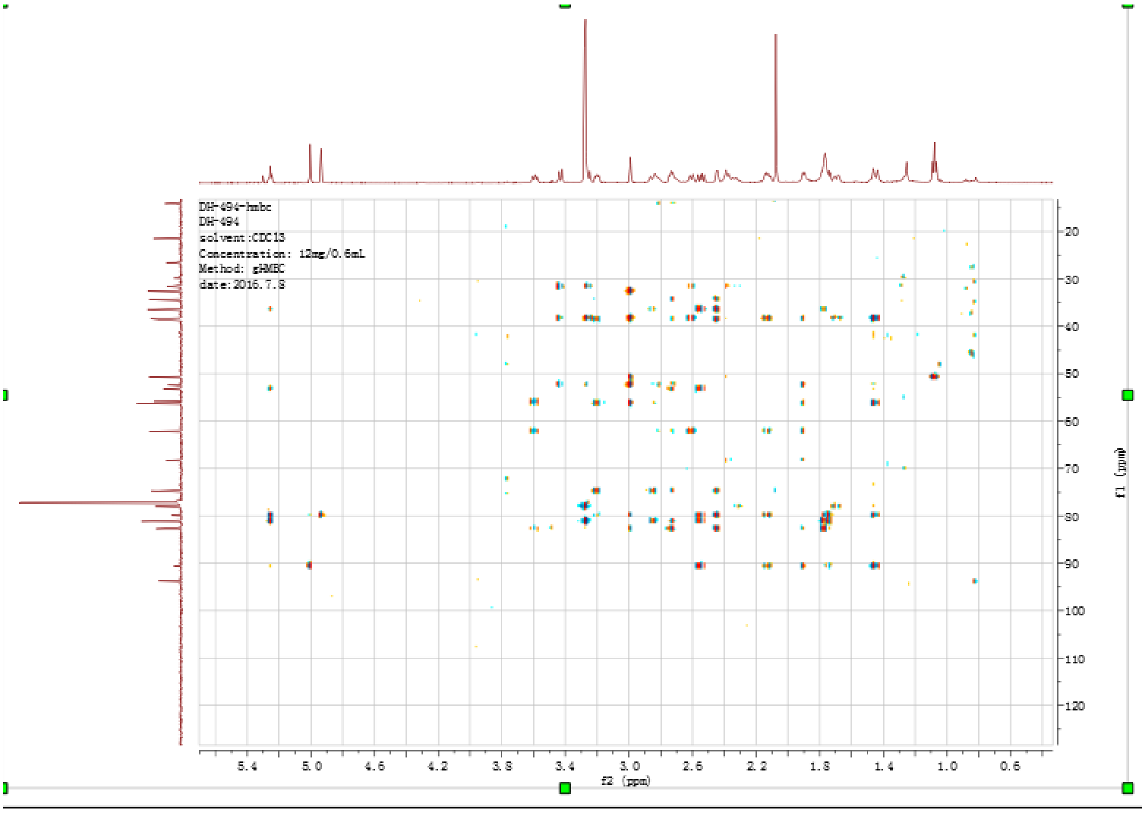

72 Noesy spectrum for compound **8**

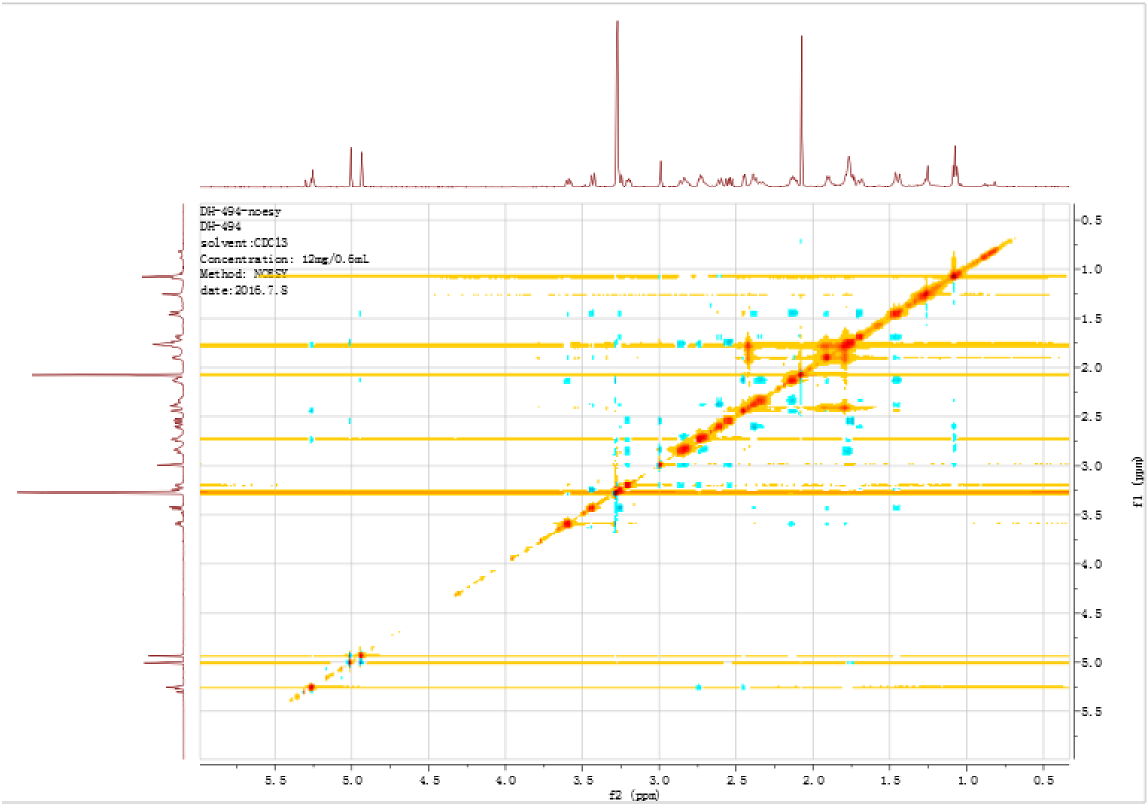

73 HR-ESI-MS spectrum for compound **9**

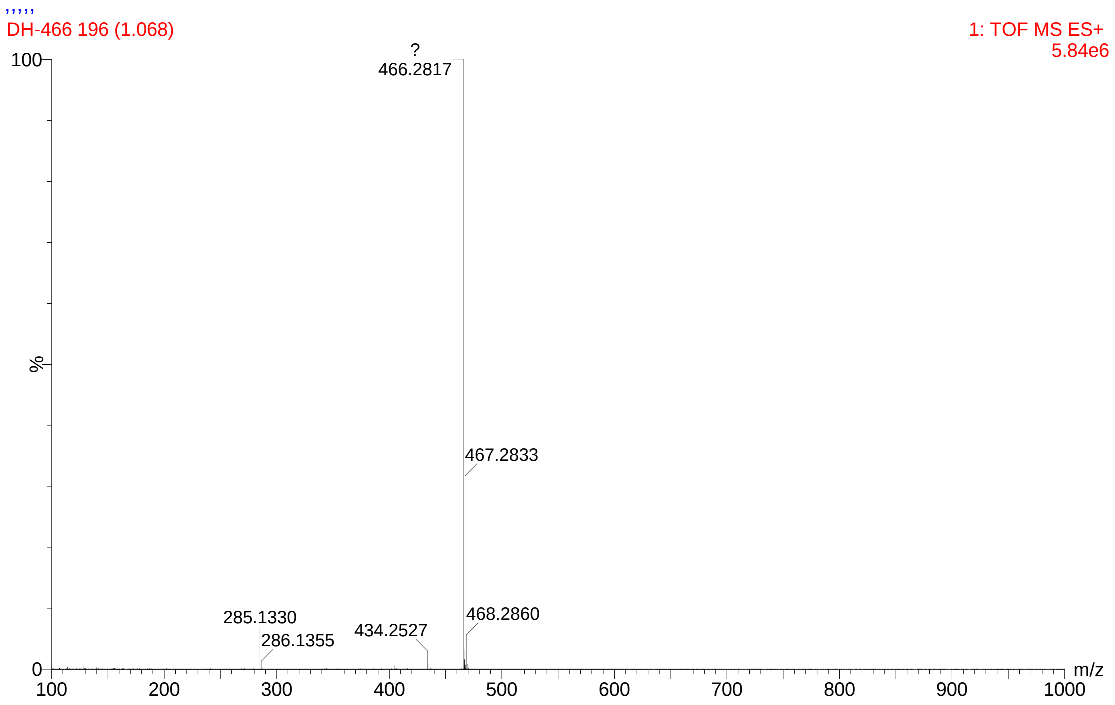

74 IR spectrum for compound **9**

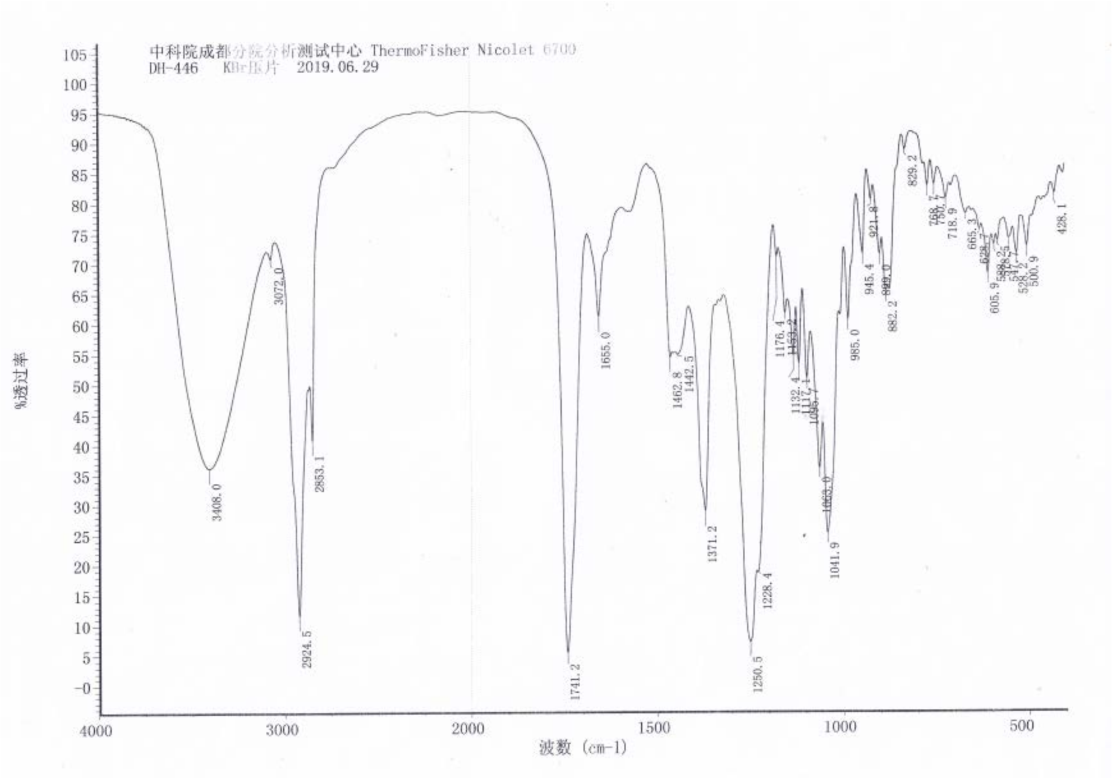

75 ^{1}H NMR spectrum for compound **9**

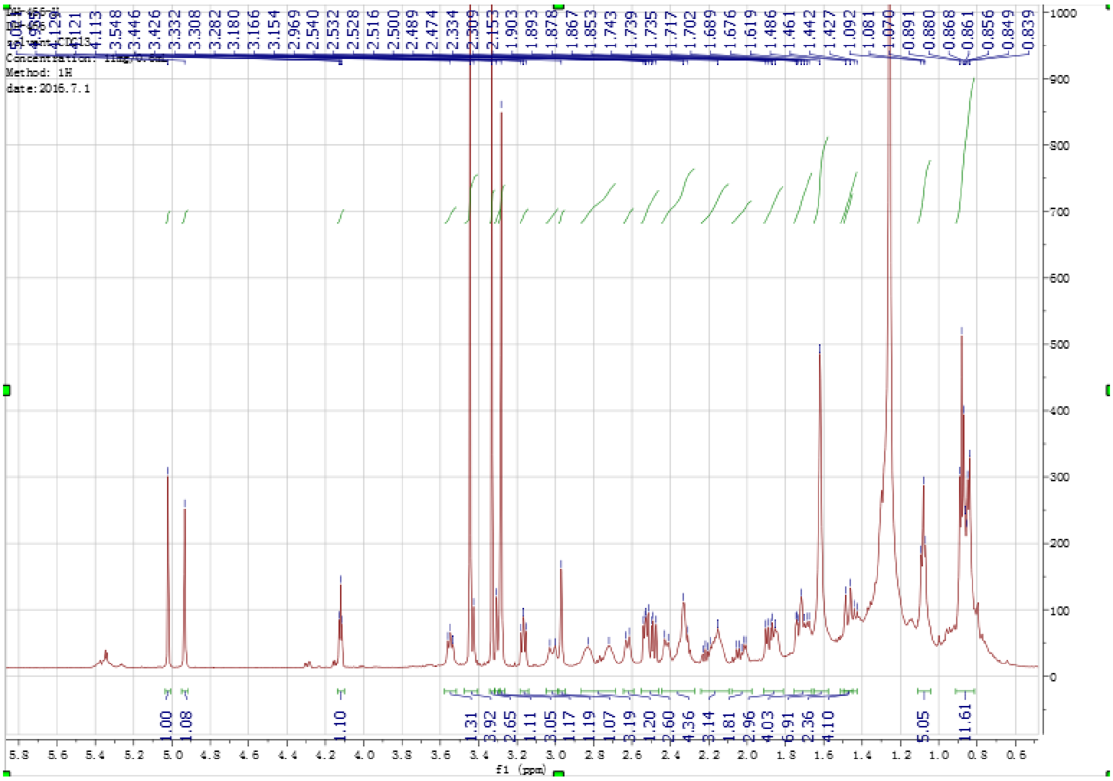

76 ^{13}C NMR spectrum for compound **9**

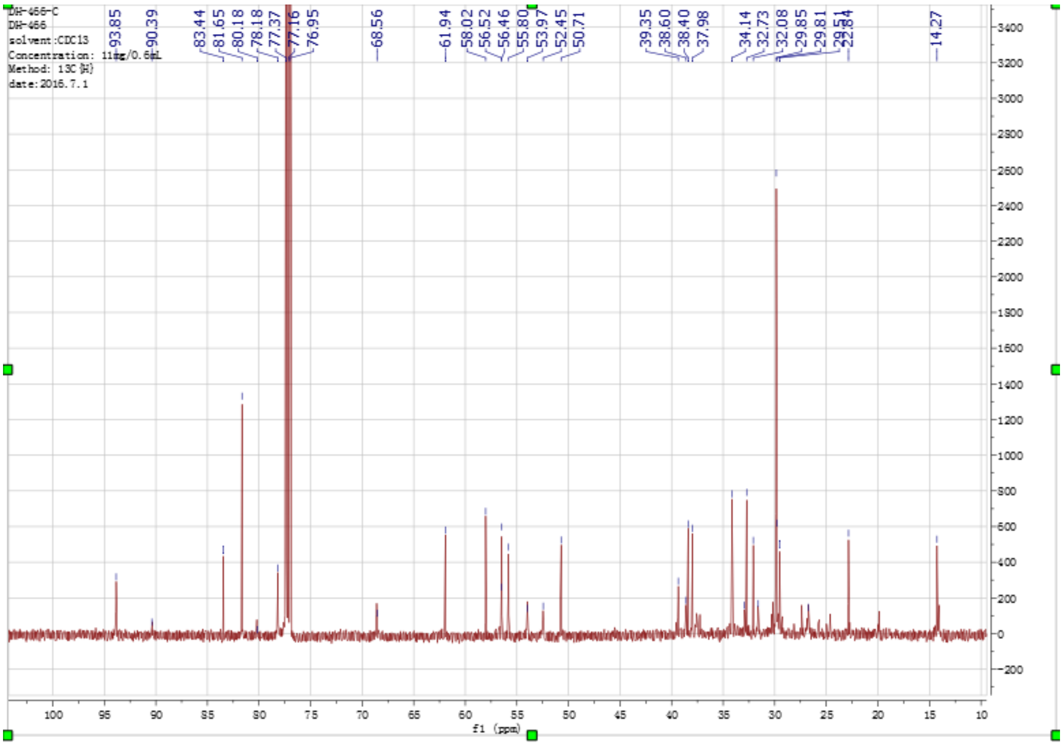

77 DEPT ($\theta = 135^{\circ}$) spectrum for compound **9**

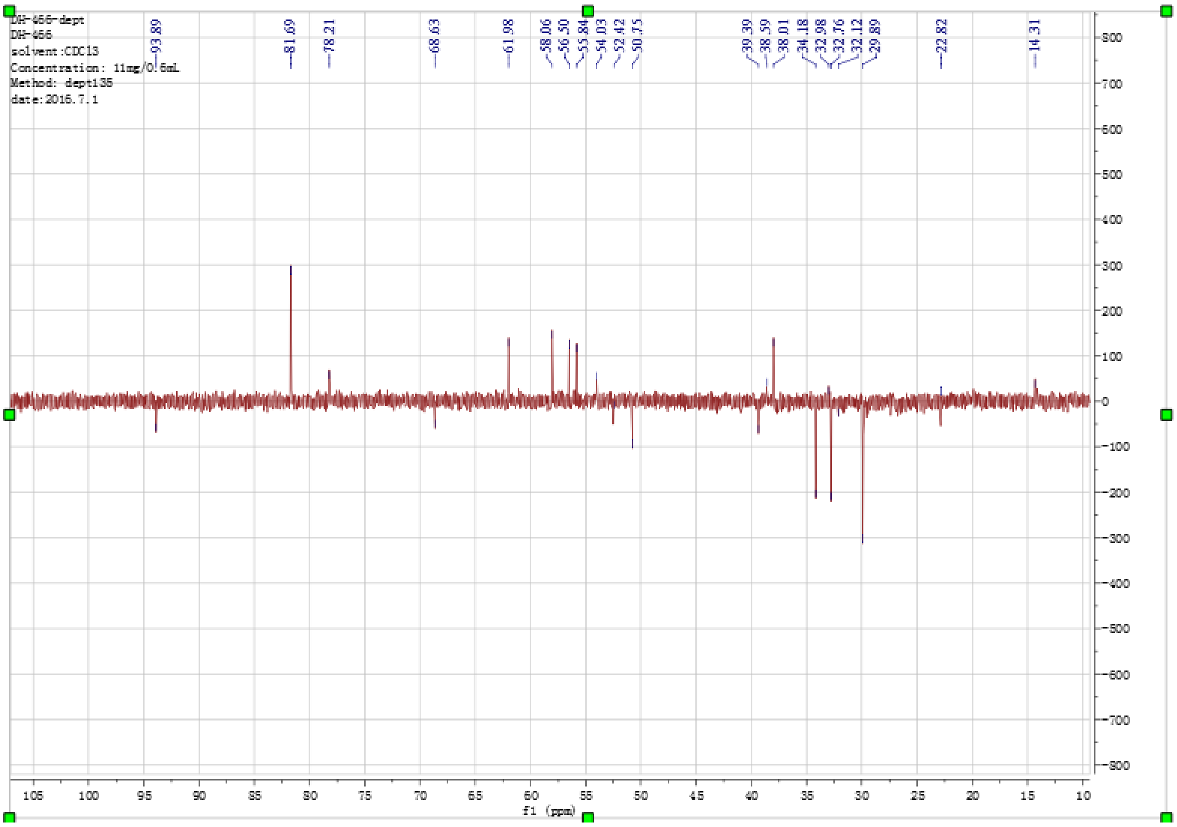

78 ^{1}H-^{1}H COSY spectrum for compound **9**

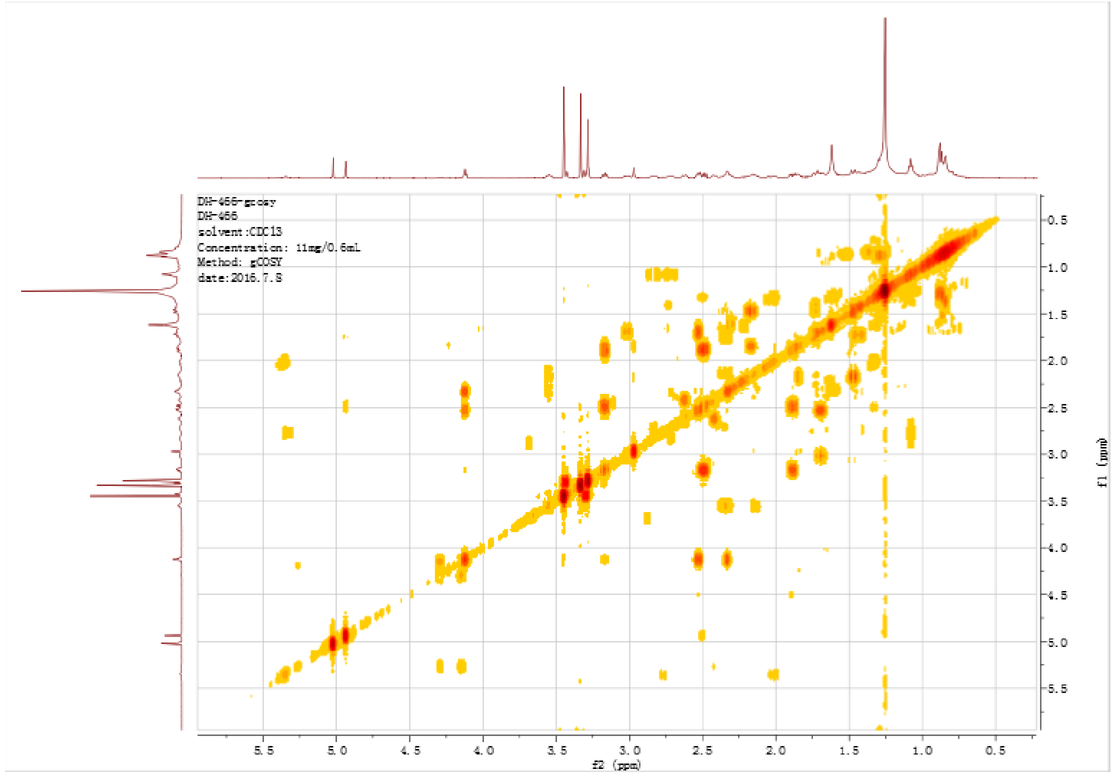

79 HMQC spectrum for compound **9**

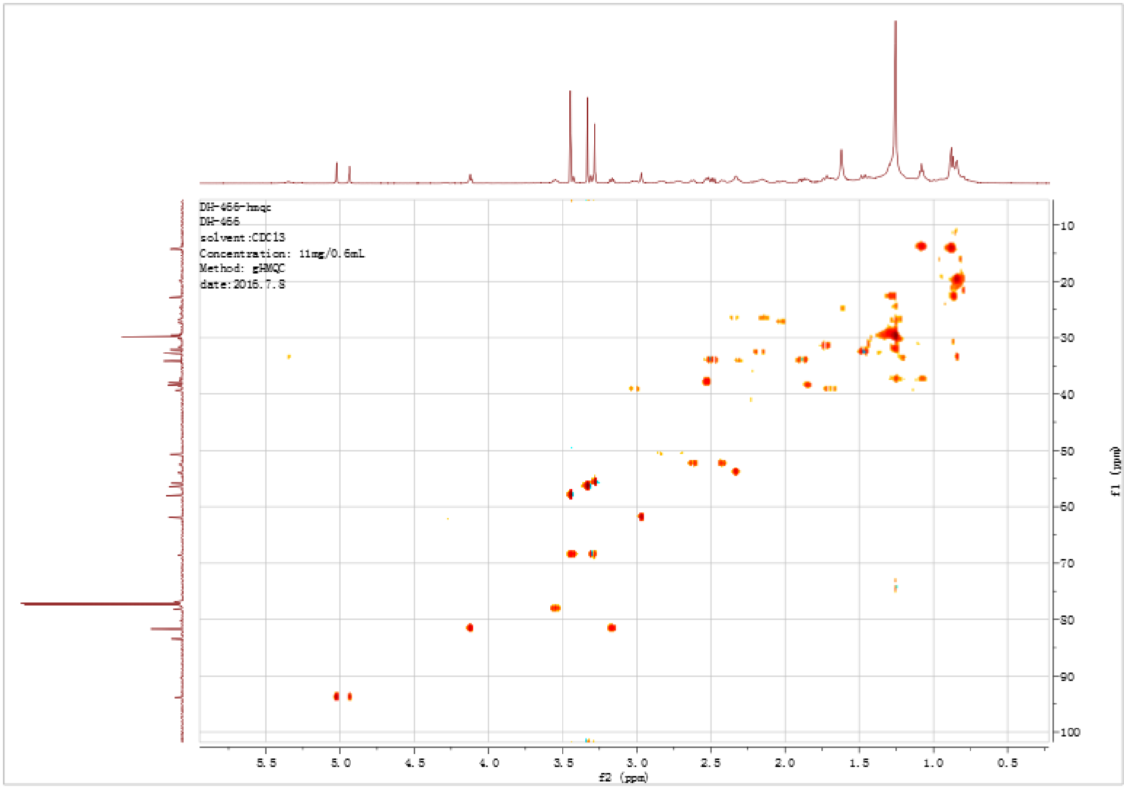

80 HMBC spectrum for compound **9**

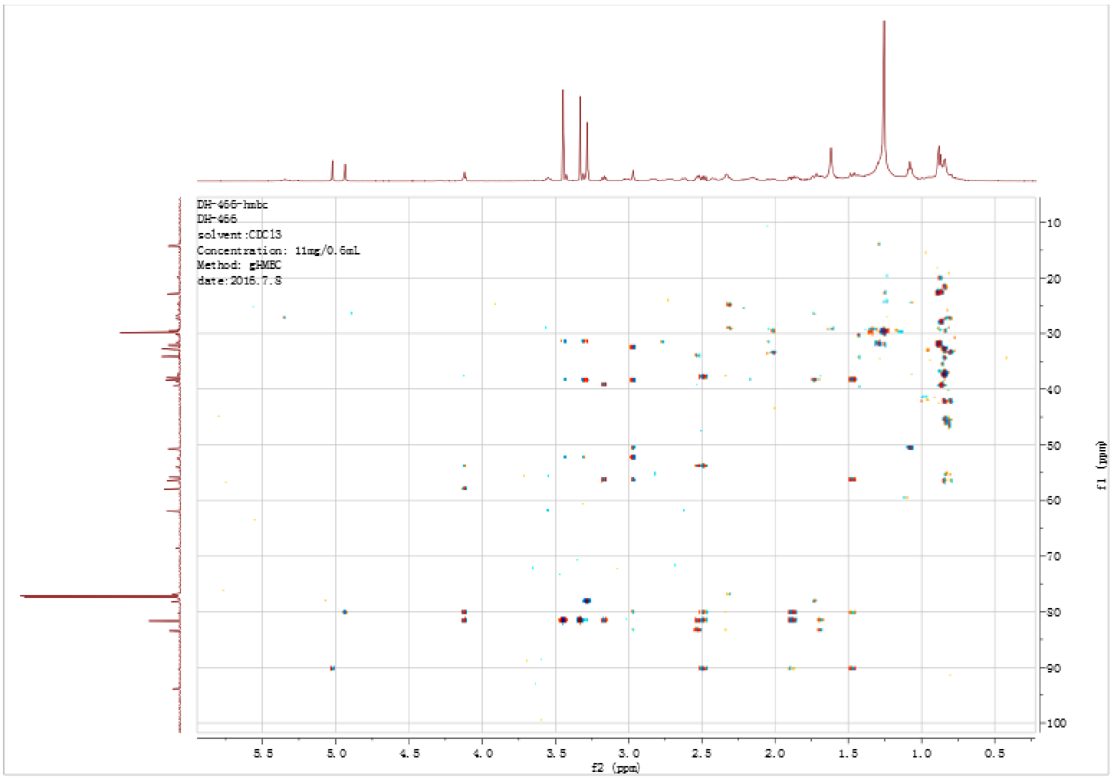

81 Noesy spectrum for compound **9**

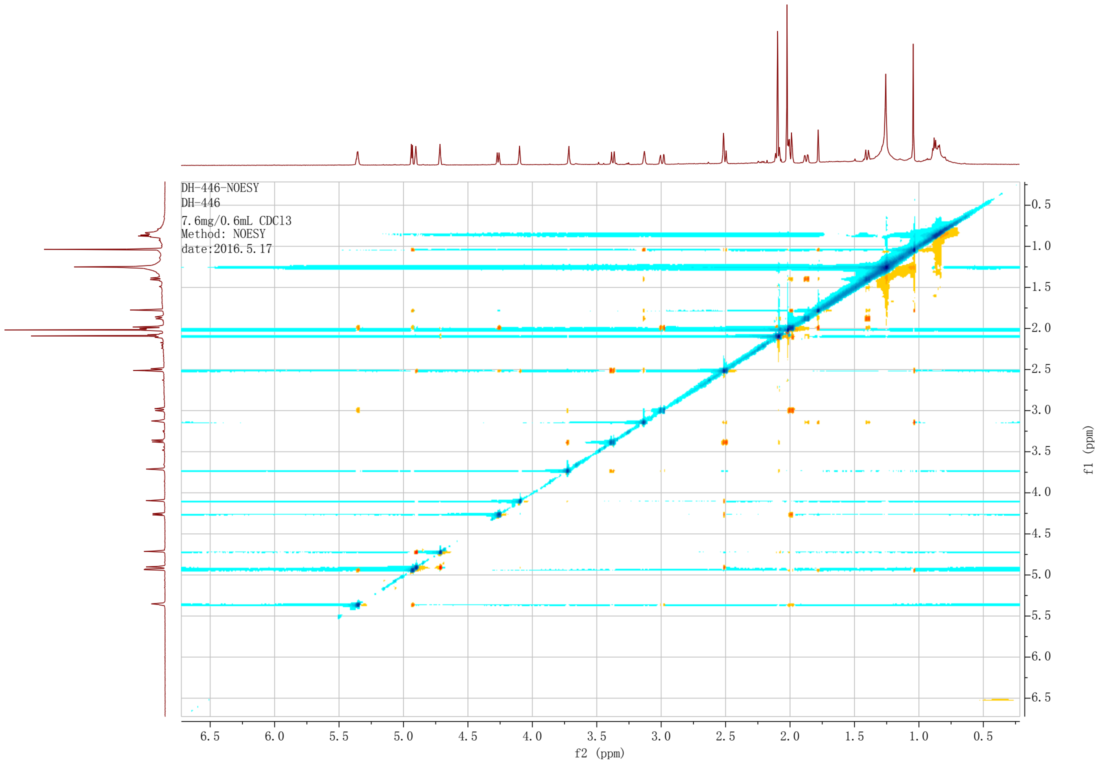

82 In Extraction and Isolation section, the 0.2 M HCl was used in the extraction process of total alkaloids, so it is possible that Anthriscifolsine A (**1**) was transformed from known compound Anthriscifolmine C (**11**) in the acid condition. According to possibility about acid promoted C-ring cleavage of diterpenoid alkaloids, known Anthriscifolmine C (**11**) was treated with 0.2 M HCl at 45 °C. The reaction solution was checked by UPLC-HRESI-MS (Q–TOF micro mass spectrometer, Waters) after 0h, 24h, 48h, 72h and 96h, respectively (**Fig.1 – Fig.5**), but no anthriscifolsine A (**1**) was observed in the reaction mixture. The result suggested that Anthriscifolmine C (**11**) cannot transform to anthriscifolsine A (**1**) in our acidic extraction condition. Of course, it is possible that compound **1** is derived from compound **11** in plant through enzymatic process.

Fig.1 UPLC-HRESI-MS spectrum for the reaction solution at 0 h.

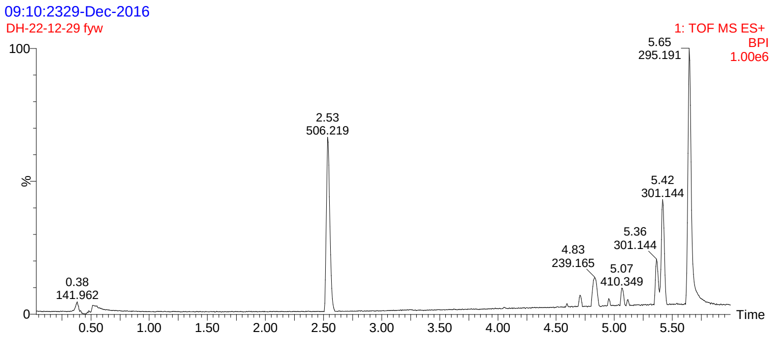

Fig.2 UPLC-HRESI-MS spectrum for the reaction solution at 24 h.

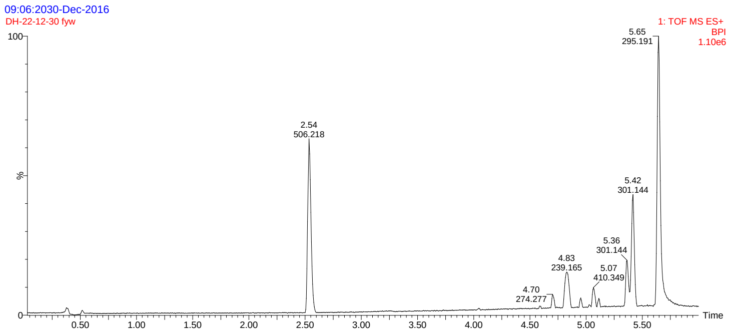

Fig.3 UPLC-HRESI-MS spectrum for the reaction solution at 48 h.

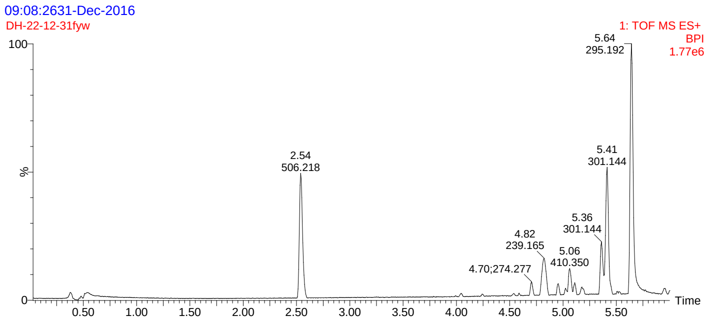

Fig.4 UPLC-HRESI-MS spectrum for the reaction solution at 72 h.

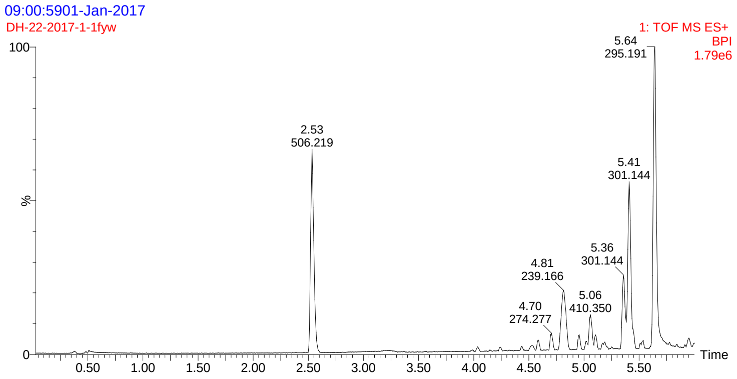

Fig.5 UPLC-HRESI-MS spectrum for the reaction solution at 96 h.

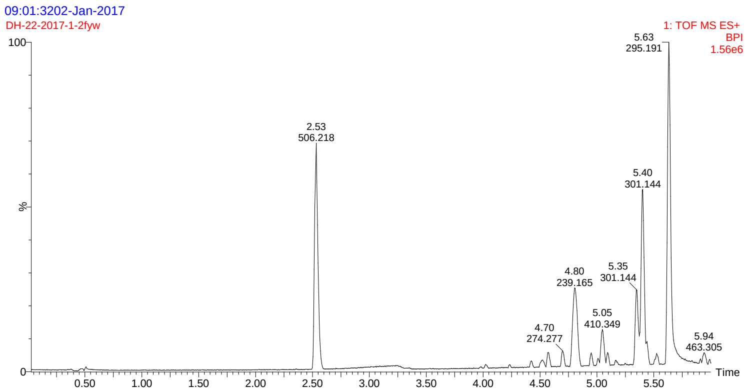

Supplement: Supplementary file 1 — Supplementary Info [file 41598_2017_5372_MOESM1_ESM.pdf]
